# Mechanochemistry for Metal–Organic Frameworks and Covalent–Organic Frameworks (MOFs, COFs): Methods, Materials, and Mechanisms

**DOI:** 10.1002/adma.202418707

**Published:** 2025-07-24

**Authors:** Joseph M. Marrett, Farshid Effaty, Xavier Ottenwaelder, Tomislav Friščić

**Affiliations:** ^1^ School of Chemistry University of Birmingham Edgbaston Birmingham B15 2TT United Kingdom; ^2^ Department of Chemistry and Biochemistry Concordia University 7141 Sherbrooke St. W. Montreal H4B 1R6 Canada

**Keywords:** covalent organic frameworks, green chemistry, mechanisms, mechanochemistry, metal–organic frameworks, solvent‐free

## Abstract

The mechanochemistry of metal‐organic frameworks (MOFs) is a well‐established field whose development has advanced the understanding and the design of both MOF materials and mechanochemical reactions. This review outlines the close and mutually beneficial interplay of these two fields over the past two decades, including the description of mechanochemical strategies to access MOFs as well as the response of these materials to mechanical treatment and/or stress. Furthermore, we highlight how the use of MOFs as model targets for mechanochemical synthesis simultaneously improves the accessibility and understanding of this class of materials and, conversely, advances the experimental and fundamental understanding of mechanochemical reactions. Similarly, we show the reciprocal benefits of comparing the mechanochemistry of organic molecular solids to that of MOFs. Finally, this review also portrays the rapid emergence of mechanochemistry of covalent‐organic frameworks, a young area that promises to deliver new, rapid, efficient, solventless, and room‐temperature access to these materials.

## Graphical abstract

An overview is provided of the mechanochemistry of metal‐organic frameworks (MOFs) and covalent‐organic frameworks (COFs), highlighting opportunities and strategies for discovery, synthesis and reactivity studies of these materials. Mechanistic studies and comparisons to the mechanochemistry of organic solids are outlined, showcasing how advances in mechanochemistry promoted the development of MOF chemistry, and vice versa.

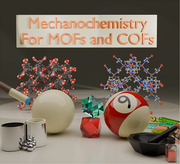

## 1 Introduction

**Figure 1:**
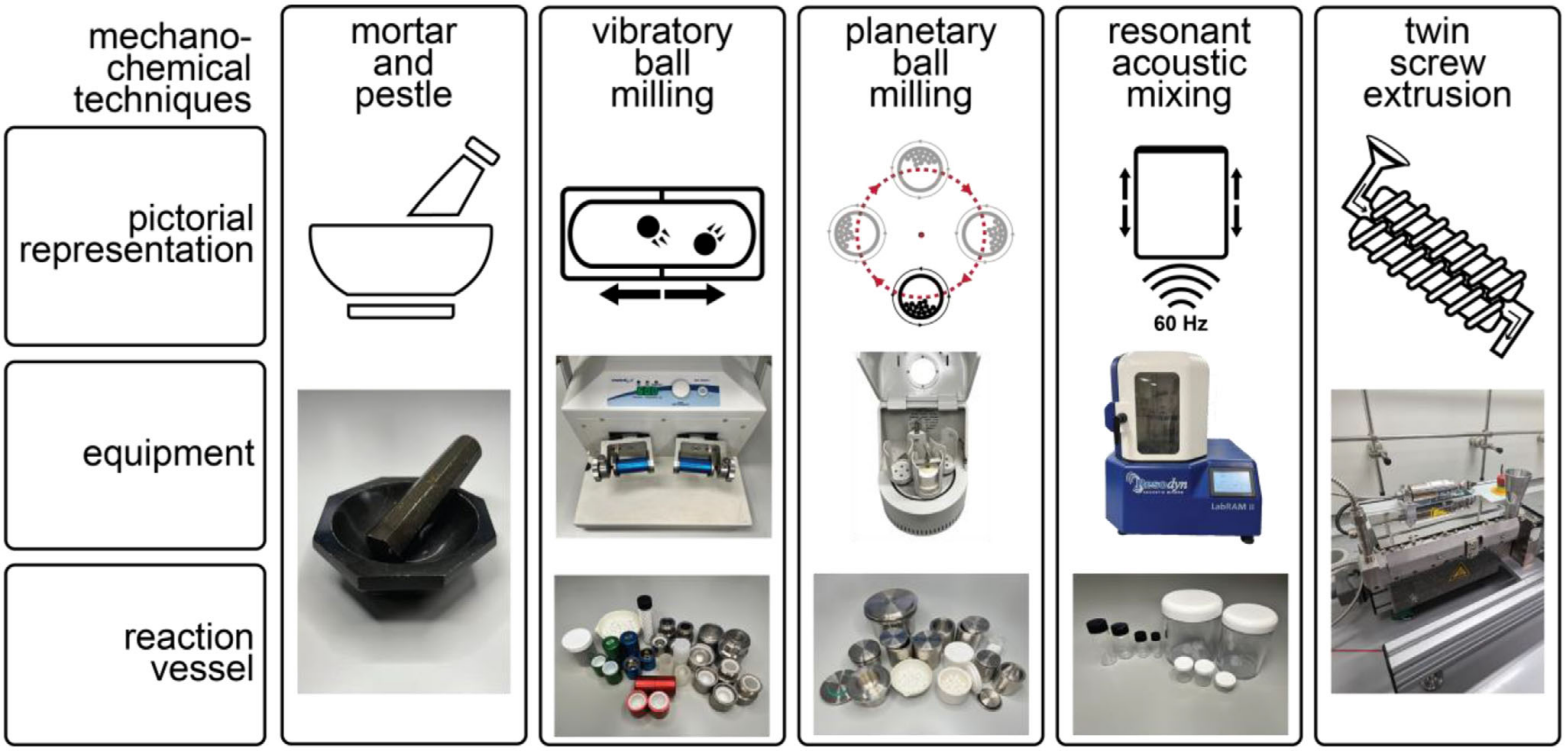
Pictorial representations of some of the popular and emerging mechanochemical methods for the synthesis of MOFs and COFs, along with illustrations of associated equipment. Twin‐screw extruder image courtesy of Dr. D. Crawford, University of Birmingham.

Over the past two decades, mechanochemical synthesis by milling, grinding, extrusion, or other types of mechanical impact and shear (**Figure** **1**) has made the leap from a laboratory curiosity to a highly effective tool applicable to a broad range of molecules and materials.^[^ ^1^ ^]^ Today, mechanochemical techniques are being explored in organic,^[^ ^2^ ^]^ main group^[^ ^3^ ^]^ and organometallic chemistry,^[^ ^4^ ^]^ with a particular emphasis on catalysis^[^ ^5^ ^]^ and the synthesis of pharmaceutically relevant molecules^[^ ^6^ ^]^ and materials (e.g., polymorphs, cocrystals).^[^ ^7^ ^]^ A particularly productive and diverse area of mechanochemistry is the synthesis of coordination polymers and metal–organic frameworks (MOFs),^[^ ^8^ ^]^ which began almost 20 years ago and has evolved to provide many cleaner, greener, more economical approaches to synthesize such materials. This area has also enabled materials discovery and helped expand the fundamental understanding of both MOF design and mechanochemical reactivity. This understanding is now being applied to devise new, effective routes for making other advanced materials, notably covalent–organic frameworks (COFs).

The connection between MOFs and mechanochemistry is reciprocal: focus on MOF synthesis has also led to major advances in mechanochemical synthesis. For example, specific mechanochemical synthetic methodologies were developed with the primary intention of employing poorly soluble metal oxides as MOF precursors. Likewise, the development of methods for direct, real‐time, and in situ monitoring of mechanochemical reaction mechanisms initially relied on MOFs as model systems. Thus, many recent innovations in equipment for mechanochemical synthesis and in the methods for experimental or theoretical mechanistic studies of mechanochemical reactions have engaged MOFs. Therefore, and perhaps somewhat non‐conventionally, the intention of this review is not solely to discuss how mechanochemistry can promote MOF synthesis, but also to present MOF mechanochemistry as a uniquely productive area that benefits innovation and fundamental understanding in both materials chemistry and mechanochemistry. In what follows, our aim is not to provide a comprehensive review but to highlight the advantages and synthetic potential of mechanochemical strategies, outlining how mechanochemistry can aid the fundamental understanding and design of MOFs, for example by supporting experimental and theoretical studies. This review also underscores the continuously emerging similarities between the mechanochemistry of MOFs and that of organic molecular solids, aiming to provide an overview that might be of interest and inspiring to MOF and solid‐state organic chemists alike. Finally, the nascent area of mechanochemical COF synthesis is discussed, with the intention of encouraging the development of new, exciting chemistries and more efficient, cleaner approaches to this kind of materials.

### 1.1 The Appeal of Mechanochemistry

Perhaps the most appealing aspect of mechanosynthesis, especially in the context of commercialization and manufacturing of advanced materials such as MOFs, is the high efficiency of materials and energy use. As these aspects of mechanochemistry have been outlined in recent reviews,^[^ ^9^ ^]^ including comprehensive ones specifically focusing on MOF synthesis,^[^ ^8^, ^10^ ^]^ these topics will not be discussed in detail. It is important to note, however, that the ability of mechanochemical techniques to operate using little or no solvent provides an attractive and powerful opportunity to generate valuable MOF materials directly from readily accessible, mineral‐like feedstocks such as oxides or carbonates. While seen as desirable by industry,^[^ ^11^ ^]^ such feedstocks are, due to their poor solubility, typically not amenable to conventional solution or solvothermal techniques. Circumventing bulk solvents and high temperatures also provides such benefits as safer manufacturing, lower costs, reduced energy consumption, and reduction in toxic solvent waste resulting from MOF synthesis. Importantly, avoiding solvothermal environments opens access to materials that would be sensitive to elevated temperatures, thus expanding the scope of MOF production. The synthesis of MOFs by mechanochemistry is also scalable, either through batch methodologies, including the highly scalable Resonant Acoustic Mixing (RAM),^[^ ^12^ ^]^ or through application of continuous mechanochemical processing in the form of twin‐screw extrusion (TSE)^[^ ^13^ ^]^ – a methodology that was highlighted in 2019 by the International Union for Pure and Applied Chemistry (IUPAC) as one of the ten technologies that can change the world.^[^ ^14^ ^]^ The value of mechanochemistry for MOF synthesis is enhanced by advances in mechanistic understanding obtained through stepwise or real‐time in situ monitoring techniques,^[^ ^15^ ^]^ which shed light on the stability, polymorphism, solid‐form landscapes as well as kinetics and thermodynamics of MOF formation.

## 2 Techniques for Mechanochemical Synthesis and Transformations of MOFs

There exists a wide range of technologies that can promote mechanochemical reactions, from the simplest mortar‐and‐pestle to shaker mills, planetary mills, magnetic mills, attrition mills, single‐ and twin‐screw extrusion systems, and many more.^[^ ^9^, ^16^ ^]^ However, preference has grown over the past two decades for a small number of technologies that can be used on the laboratory bench as well as in specialized facilities, such as synchrotron radiation sources. This section provides an overview of these most encountered technologies, with their schematic representations shown in Figure 1.

### 2.1 Mortar‐and‐Pestle Grinding

The mortar and pestle are most likely the oldest and best known instruments for mechanochemistry. Manual grinding using a mortar and a pestle was used by Musgrave and Mattson in what may have been the first synthesis of an open metal–organic framework material.^[^ ^17^ ^]^ Specifically, grinding a slurry of metal nitrates and 4,4′‐dipyridyl (**bipy**) in anhydrous methanol (MeOH) yielded materials of composition M(**bipy**)_2_(NO_3_)_2_ (M = Co, Ni, Cu). Based on UV–vis and infrared reflectance spectroscopy, the resulting Co^II^ and Ni^II^ complexes were identified as square‐grid sheets of octahedral metal ions bridged by **bipy** molecules. Several other MOFs and coordination polymers have been synthesized by manual grinding,^[^ ^18^ ^]^ and further examples of such syntheses are outlined in the subsequent sections of this review.

### 2.2 Ball‐Milling

#### 2.2.1 Neat Grinding

**Figure 2:**
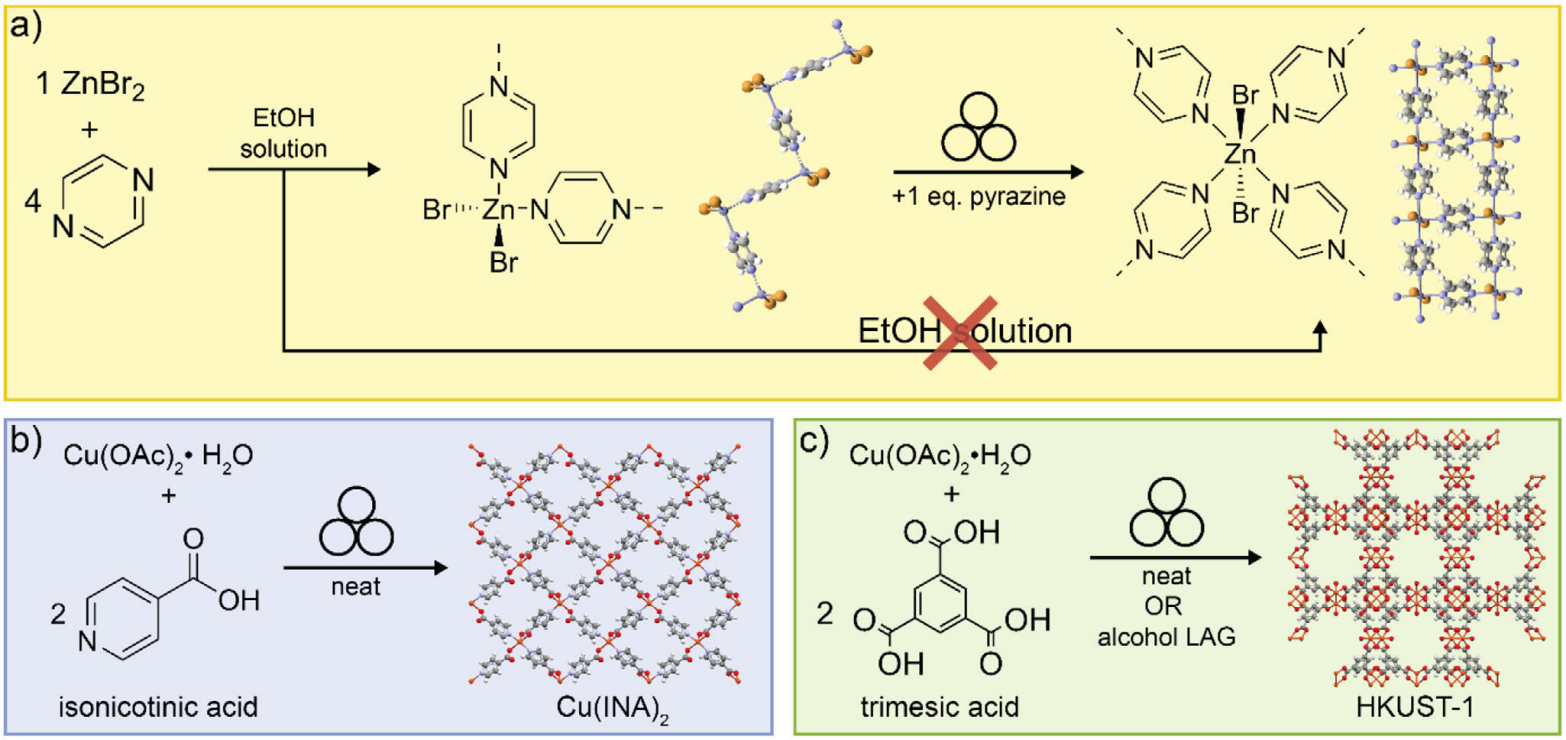
Early examples of the mechanochemical synthesis of open coordination polymers and MOFs. a) The solution reaction of ZnBr_2_ and pyrazine produces a 1D coordination polymer, which can be converted mechanochemically to a 2D square‐grid layered (*sql*) structure.^[^ ^19^ ^]^ b) Mechanochemical synthesis of 3D open Cu(**ina**)_2_ framework by neat milling of Hina and Cu(OAc)_2_·H_2_O.^[^ ^21^ ^]^ c) Mechanochemical synthesis of HKUST‐1 by milling of Cu(OAc)_2_·H_2_O and H_3_ **btc** with or without a liquid additive.^[^ ^23^ ^]^

One of the first examples of an open‐framework material made mechanochemically comes from the Nassimbeni group,^[^ ^19^ ^]^ who in 2001 explored the reaction of ZnBr_2_ with the ditopic heterocyclic ligand pyrazine (**pyz**) (**Figure** **2**a). Whereas the reaction in ethanol solution produced the 1D zig‐zag‐topology coordination polymer ZnBr_2_(**pyz**), grinding a 1:1 mixture of this polymer with pure **pyz** in a vibratory ball mill led to 2D square‐grid material ZnBr_2_(**pyz**)_2_. This work demonstrated how a product of different stoichiometric composition and framework topology could be obtained by switching from solution‐ to milling‐based chemistry. Importantly, the product of mechanochemical reaction exhibited the stoichiometric composition imposed by the amounts of reactants added, illustrating notable stoichiometric precision compared to solution experiments, which produced the 1D‐polymer ZnBr_2_(**pyz**) even when a fourfold excess of **pyz** was used. Such stoichiometric precision is a hallmark of mechanochemical reactions and was observed in, for example, selective synthesis of cadmium coordination polymers with cyanoguanidine and across a wide range of cocrystallization, catalytic, and other reactions.^[^ ^20^ ^]^

The first formal, targeted mechanochemical synthesis of a 3D MOF material was reported in 2006 by the James group,^[^ ^21^ ^]^ whose pioneering report outlined the neat mechanochemical reaction of copper(II) acetate with isonicotinic acid (H**ina**) to obtain the open‐framework copper(II) isonicotinate, Cu(**ina**)_2_ (Figure 2b). Milling of the reactants for 10 min in a vibratory ball‐mill produced the 3D Cu(**ina**)_2_ framework with high crystallinity, as evidenced by sharp reflections in the powder X‐ray diffraction (PXRD) pattern. The synthesized MOF contained reaction byproducts water and acetic acid, which could be removed by heating to yield a MOF with absorption properties on par with those of analogous materials obtained solvothermally.^[^ ^21^ ^]^ This pioneering report should be seen as a major advance, introducing mechanochemistry as a simple, efficient and rapid means to synthesize open‐framework 3D materials. Interestingly, the Cu(**ina**)_2_ framework could also be obtained through a mechanically activated aging process, i.e., by milling of reactants for 1 minute followed by aging.^[^ ^22^ ^]^

This work was followed in 2008 by a broad survey of mechanochemical reactivity for MOF synthesis.^[^ ^23^ ^]^ Through a large systematic investigation of Zn^II^‐, Cu^II^‐, and Ni^II^‐based precursors and bridging organic ligands totaling more than 50 screening reactions, both previously reported and previously unknown coordination polymers were produced. This included the mechanochemical synthesis of the commercially‐relevant HKUST‐1 framework, obtained in quantitative conversion by dry grinding of Cu(OAc)_2_·H_2_O with 1,3,5‐benzenetricarboxylic acid (H_3_ **btc**) (Figure 2c). Importantly, this work began to identify factors that increase the likelihood of reaction between organic linker and metal source: low melting points of reactants, basicity of the metal salt anion, hydration of the starting materials, and release of liquid byproducts during reaction. The synthesis of HKUST‐1 was subsequently investigated in more detail, revealing that materials of high surface area, after washing with ethanol, were obtained readily by milling times as short as 5 min.^[^ ^23^ ^]^

Imidazolate‐based MOFs have also been made under neat ball‐milling conditions, as illustrated by Tanaka et al.^[^ ^24^ ^]^ Milling ZnO with 2‐methylimidazole (H**Meim**) produced core–shell structures comprising a core of unreacted ZnO surrounded by a *ca*. 20 nm‐thickness polycrystalline shell of sodalite‐topology (SOD‐topology) Zn(**Meim**)_2_, also known as ZIF‐8. Similar observations were made by Taheri et al. who investigated ZIF‐8 synthesis by ball‐milling of nanosized ZnO with H**Meim**, followed by high‐temperature treatment of the sample at 180 °C.^[^ ^25^ ^]^ While Lin et al. had previously reported that ZnO and H**Meim** react at high temperatures, with 180 °C being optimal to reach conversion within hours,^[^ ^26^ ^]^ Taheri et al. found that complete conversion of ZnO nanoparticles to ZIF‐8 by neat milling followed by thermal treatment was hindered by ZnO particle agglomeration, and obtained core–shell structures instead.^[^ ^25^ ^]^ To produce phase‐pure ZIF‐8 of high surface area required milling for 8 h in the presence of methanol, followed by treatment at 180 °C.

The formation of ZnO‐containing core–shell structures of ZIF‐8^[^ ^24^ ^]^ reflects a general limitation of neat mechanochemistry: that reactivity can be hindered when the product particles grow around and encapsulate the reactant particle. A similar phenomenon was observed in the mechanochemistry of inorganic materials, where ball‐milling was found to produce nanometer‐scale shells of mechanically‐activated amorphous material on the surface of metal oxides.^[^ ^27^ ^]^ Such mechanically activated amorphous surface shells are expected to be more reactive than the crystalline interior of the particles and might also play a role in the mechanochemical formation of ZIF‐8 on surface of ZnO particles.

Most recently, Xue and co‐workers have reported the synthesis of cadmium‐based ZIF materials by ball‐milling of CdO and imidazole linker precursors in the presence of a catalytic amount (ca. 1 mol%) of Cd(OAc)_2_·2H_2_O.^[^ ^28^ ^]^ Inorganic precursors other than metal oxides, such as metal carbonates or hydroxides can react readily under neat milling conditions.^[^ ^29^ ^]^ Even hydrides have been used efficiently for MOF mechanosynthesis without liquid additives, as illustrated by the Balema group who prepared an yttrium‐based framework solid by milling YH_3_ with H_3_ **btc**.^[^ ^30^ ^]^

#### 2.2.2 Liquid‐Assisted Grinding (LAG): Green Chemistry and Stoichiometric Precision

A highly effective approach to conduct mechanochemical reactions is liquid‐assisted grinding (LAG),^[^ ^31^ ^]^ a methodology that uses amounts of a liquid phase that are typically orders of magnitude smaller than those used in conventional solution‐based synthesis to induce, accelerate or even direct mechanochemical reactions. The amount of liquid phase is conveniently expressed by the parameter *η*,^[^ ^32^ ^]^ defined as the ratio of the volume of liquid additive to the weight of the reactants, usually expressed in µL/mg. An early empirical cocrystallization study placed LAG conditions in the *η*‐range from 0 to ca. 1 µL/mg, followed by slurries and homogeneous solutions at increasing *η* values. The benefits of LAG (also known as solvent‐drop grinding, SDG,^[^ ^33^ ^]^ and related to kneading^[^ ^34^ ^]^) have been demonstrated across a wide range of chemical and materials transformations, from catalysis, organic, organometallic and main group synthesis, to the synthesis of cocrystals, nanoparticles and hydrogen‐bonded organic frameworks. Some of the pioneering reports of using liquid additives in the synthesis of metal–organic materials came from the Braga group, exemplified by the synthesis of a 1D coordination polymer by mortar‐and‐pestle kneading of copper(II) chloride (CuCl_2_) with *trans*‐1,4‐diaminocyclohexane (**dace**) and a small amount of water or *S,S*‐dimethylsulfoxide (DMSO) over 5 to 10 min.^[^ ^35^ ^]^ The resulting 1D polymer was found to be remarkably capable of harboring and releasing small molecular guests in the solid state.

**Figure 3:**
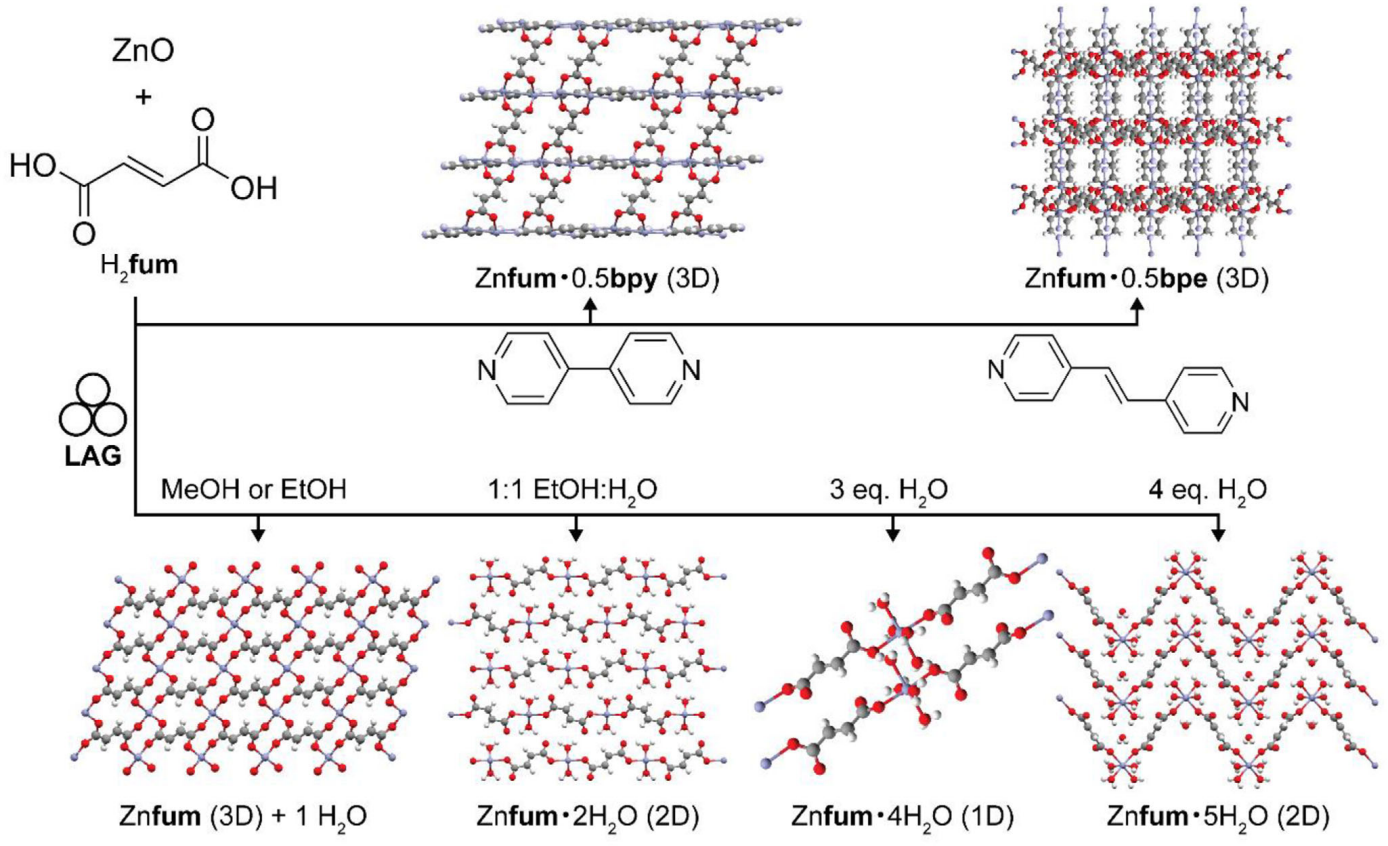
Observed outcomes of the mechanochemical reaction of ZnO with fumaric acid as a function of the liquid additive and the introduction of a pillar‐forming additive.^[^ ^36^, ^40^ ^]^

In 2009, our group used LAG for a MOF synthesis that hinged on the in situ activation of zinc oxide (ZnO).^[^ ^36^ ^]^ Metal oxides are advisable precursors for MOFs due to their accessibility and safety of handling, but their poor solubility makes them ill‐suited for solution chemistry. Conversely, soluble starting materials such as metal nitrates and chlorides are considered problematic by the industry (more toxic, corrosive, and/or unsafe to handle).^[^ ^11^ ^]^ Thus, mechanochemistry was used to explore the synthesis of a simple zinc fumarate model polymer by milling ZnO with fumaric acid (H_2_ **fum**) (**Figure** **3**). Whereas no reaction was observed under neat grinding, LAG in the presence of methanol (MeOH) or ethanol (EtOH) led to the formation of a previously unknown Zn(**fum**) 3D framework, which was then characterized using PXRD data. Moreover, using a 1:1 mixture of water and ethanol produced another novel material, the dihydrate Zn(**fum**)·2H_2_O, while LAG with three or four equivalents of water selectively produced the known tetra‐ and pentahydrates, Zn(**fum**)·4H_2_O and Zn(**fum**)·5H_2_O, respectively (Figure 3). This work highlighted the potential of LAG mechanochemistry in combination with structure analysis by PXRD to discover and characterize new metal–organic phases.^[^ ^36^ ^]^ It also showcased notable atom economy, a characteristic feature^[^ ^37^ ^]^ of mechanochemistry: the Zn(**fum**)·4H_2_O and Zn(**fum**)·5H_2_O product had incorporated all of the liquid additive as well as the water formed as reaction byproduct. Adding a ditopic pyridine‐based ligand such as 4,4’‐bipyridine (**bipy**) or *trans*‐1,2‐bis(4‐pyridyl)ethylene (**bpe**) to the reaction mixture and using organic solvents as liquid additives enabled the one‐pot LAG synthesis of open‐framework pillared 3D MOFs directly from ZnO, including materials not characterized previously. The outlined synthesis of a complex 3D open framework material containing paddlewheel metal nodes and two types of ligands, directly from a metal oxide, is an illustration of the high potential of mechanochemistry for reaction simplification and stoichiometric precision.

The use of a liquid additive in LAG generally helps increase the speed of reaction, providing an opportunity to conduct MOF synthesis within minutes or hours, as opposed to the hours or days required by solvothermal processes. While this aspect of mechanochemical MOF synthesis is outlined in contemporary reviews,^[^ ^38^ ^]^ a recent example is the synthesis of the microporous MOF Mg_2_(*m*‐**dobdc**) (dobdc = 4,6‐dioxidobenzene‐1,3‐dicarboxylate) by Chen et al.^[^ ^39^ ^]^ Whereas the optimized synthesis of the material at 0.5 gram scale required 48 h solvothermal synthesis in toxic *N*,*N*‐dimethylformamide (DMF) solvent, a mechanochemical ball‐milling procedure accomplished the synthesis within 10 min, without requiring DMF. Notably, the material prepared mechanochemically exhibited superior properties to the one resulting from optimized solvothermal synthesis.^[^ ^39^ ^]^

While the exact role of the liquid additive in LAG reactions is not fully understood and is a topic of ongoing research,^[^ ^40^ ^]^ several studies have investigated how the choice and/or amount of liquid phase affects the product of mechanochemical synthesis of MOFs, and coordination polymers in general. For example, the systematic study of the mechanochemical synthesis of Zn(**fum**)‐based coordination polymers by Strobridge et al. indicated that the formation of anhydrous or differently hydrated materials Zn(**fum**), Zn(**fum**)·2H_2_O, Zn(**fum**)·4H_2_O and Zn(**fum**)·5H_2_O by LAG can be correlated to the activity, or the mole fraction, of water in the liquid additive.^[^ ^41^ ^]^ The use of a liquid additive that reduces water activity, such as MeOH, favored the formation of the anhydrous framework material despite water being a reaction byproduct. In contrast, using liquid additives that do not decrease water activity, such as butanol or acetonitrile facilitated the formation of hydrated coordination polymers. Moreover, investigation of the reaction in the presence of a limited amount of water revealed a stepwise reaction mechanism, resulting from the inclusion of the water liquid additive in the crystal structure of the product. At the onset of milling, the reaction of ZnO proceeded rapidly under LAG conditions, with the water additive becoming incorporated in the first reaction product, Zn(**fum**)·5H_2_O. Upon consumption of the entire amount of the water liquid additive, the reaction switched into a slower, neat grinding process that led to amorphization followed by crystallization of the final product, Zn(**fum**)·4H_2_O, thus illustrating how the amount of liquid additive can be important for the reaction progress. The intermediate appearance of an amorphous phase was consistent with the observation that LAG generally yields products of higher crystallinity compared with neat grinding.^[^ ^42^ ^]^ The ability to obtain different hydrated metal–organic phases by varying water activity in the liquid additive was subsequently used to synthesize known, and discover new, magnesium‐based complexes and coordination polymers of the active pharmaceutical ingredient (API) naproxen.^[^ ^43^ ^]^

The liquid additive can affect the formation of a targeted metal–organic product in either a positive or a negative way. For example, Allenbaugh and Shaw reported that the mechanochemical synthesis of Pd^II^ bipyridine complexes by LAG in the presence of DMSO proceeds through the intermediate formation of a reactive DMSO‐containing complex that further reacts to give the final, DMSO‐free solid product.^[^ ^44^ ^]^ The reaction in the absence of DMSO was significantly slower. Even if this example is on a small‐molecule coordination complex rather than a MOF, it is likely that similar effects could play a role in the synthesis of MOFs. Conversely, an adverse effect of the liquid additive in the mechanochemical synthesis of a MOF was reported by Fidelli et al. in the real‐time in situ synchrotron X‐ray diffraction study of the formation of the zirconium‐based carboxylate frameworks NU‐901 and UiO‐67 by ball‐milling in the presence of DMF as a liquid additive. For example, analysis of the real‐time PXRD data revealed the concomitant formation of NU‐901 and a DMF‐containing zirconium carboxylate cluster side‐product that persisted throughout the reaction and, consequently, was a hindrance to the formation of the target NU‐901.^[^ ^45^ ^]^

A large systematic exploration of how the liquid additive affects MOF synthesis by LAG was presented by Stolar and co‐workers,^[^ ^46^ ^]^ who conducted the synthesis of HKUST‐1 in the presence of over 20 solvents and their mixtures. Real‐time monitoring of the process demonstrated that poorly coordinating or non‐coordinating liquids such as hexane, cyclohexane, CHCl_3_ led to little or no reaction acceleration compared with neat grinding. In contrast, the use of polar and protic liquids greatly enhanced HKUST‐1 formation, with methanol being most effective. The reactivity was also dependent on the amount of liquid and, notably, reaction acceleration during LAG with mixtures of protic and non‐protic liquids was dependent only on the amount of the protic liquid added. In addition to impacting the rate of reaction, the amount of liquid additive also can affect the properties of the final HKUST‐1 product, such as the relative contributions of micro‐ and mesopores to the overall porosity of the material. Specifically, Mason and co‐workers^[^ ^47^ ^]^ have shown that the BET surface area and the fraction of micropores contributing to the total sample porosity depend on the *η* value. Increasing the *η* value from 0 to ca. 0.5 µL/mg led to HKUST‐1 samples with increasingly high surface areas, and greater content of micropores. At a higher *η* value of almost 0.8 µL/mg, the reaction mixture became a slurry, with a concomitant drop in surface area and micropore content. The analysis of the sample porosity indicated that lower *η* values (i.e., those below 0.2 µL/mg) correspond to environments in which particle breakage mechanisms are dominant, leading to a product with a more pronounced mesoporous nature. At higher *η* values, however, the LAG process became dominated by particle agglomeration processes, leading to a predominantly microporous material.

**Figure 4:**
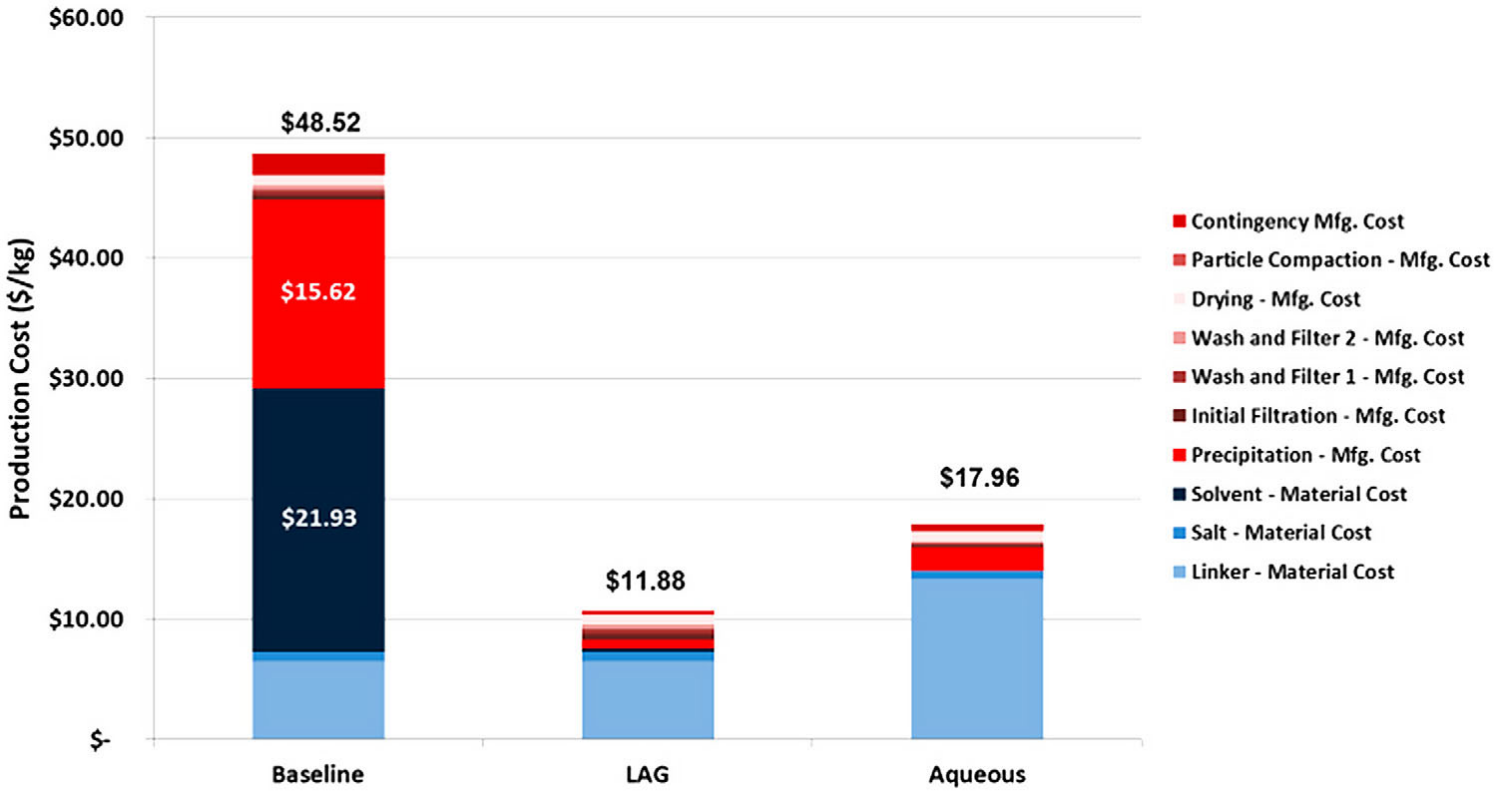
Comparison of the estimated manufacturing costs for Mg‐MOF‐74 based on the technoeconomic analysis for solvothermal (baseline) and mechanochemical (LAG) synthesis, as well as synthesis in aqueous solvent (aqueous). Reproduced with permission.^[^ ^48^ ^]^ Copyright 2017, American Chemical Society.

The effectiveness of LAG in the synthesis of commercially attractive MOF materials was investigated in a 2017 comparative technoeconomic analysis reported by DeSantis and co‐workers (**Figure** **4**).^[^ ^48^ ^]^ The analysis compared the estimated costs for the manufacture of the carboxylate‐based Mg‐MOF‐74^[^ ^49^ ^]^ material, composed of Mg^2+^ ions and anions derived from 2,5‐dihydroxyterephthalic acid (H_4_ **dhta**), via a traditional solvothermal process based on DMF solvent, a synthesis in aqueous solvent, and LAG. With the assumption that each synthetic strategy produces the target material in 92% yield, the solvothermal process performed the worst, with a material manufacturing cost of ca. $49/kg to which the most significant contributors were contingency costs and costs of using a toxic organic solvent. Using a safer solvent in the aqueous synthesis significantly reduced the manufacturing cost down to ca. $18/kg. The application of LAG led to even further cost reduction, below $12/kg, owing to reduced material costs and solvent use.

A technoeconomic analysis of mechanochemical synthesis of the zirconium‐based MOF UiO‐66‐NH_2_^[^ ^50^ ^]^ was recently reported by the D'Alessandro group.^[^ ^51^ ^]^ They developed a simplified laboratory‐scale synthesis of the material and found that a manufacturing cost in the range from $3,393 to $12,495 per kilogram would be realistic. The mid‐range cost assessment, $6,498/kg, was comparable to prices of then commercially available MOFs. Importantly, transitioning from a batch milling to a continuous process, for example by twin‐screw extrusion (TSE), was anticipated to bring further major reductions in the production costs. In that context, a continuous wet ball milling approach for the synthesis of zirconium‐based MOFs was recently reported.^[^ ^52^ ^]^

Finally, it is relevant to note that a particular strength of LAG in the mechanochemical synthesis of MOFs is the introduction of additional parameters for the optimization of the process, for example, the choice and amount of liquid additive. Sometimes, however, the greatest benefits can be accomplished by varying the choice of starting material. For example, Brekalo et al. showed that the LAG synthesis of ZIF‐8 from ZnO and H**Meim** only resulted in partial conversions, and that the effective synthesis of ZIFs by LAG hinged on using a more reactive metal precursor, basic zinc carbonate, 2ZnCO_3_·3Zn(OH)_2_.^[^ ^53^ ^]^ It was subsequently reported that the reaction of 2ZnCO_3_·3Zn(OH)_2_ with H**Meim** proceeded even upon simple contact of the two substances.^[^ ^29^ ^]^

#### 2.2.3 Ion‐ and Liquid‐Assisted Grinding (ILAG): Activation of Oxide Reactant

While the LAG methodology offers a powerful means by which to increase reactivity and to select for different chemical outcomes during MOF synthesis, the addition of simple inorganic salts can provide further reactivity enhancement. This approach, termed ion‐ and liquid‐assisted grinding (ILAG)^[^ ^54^ ^]^ was first deployed for the synthesis of open pillared MOF materials based on terephthalic acid (H_2_ **ta**) and 1,4‐diazabicyclo[2.2.2]octane (**dabco**), as originally reported by the groups of Kim and of Hupp.^[^ ^55^, ^56^ ^]^ Specifically, attempts to synthesize the Zn_2_(**ta**)_2_(**dabco**) MOF by neat milling or LAG of ZnO, **dabco** and H_2_ **ta**, in a manner analogous to previous mechanosynthesis of pillared MOF involving H_2_ **fum** and **bipy**, were unsuccessful, yielding mostly the salt [**dabco**H^+^][H**ta** ^−^]. The addition of small amounts of an alkali metal nitrate or ammonium nitrate, however, readily promoted the formation of the tetragonal *tet*‐Zn_2_(**ta**)_2_(**dabco**) framework, with square grids of zinc terephthalate bridged by **dabco** units. Switching to the corresponding sulfate salts, however, led to the formation of a hexagonal polymorph,^[^ ^57^ ^]^ *hex*‐(Zn_2_(**ta**)_2_(**dabco**), with Kagome‐type layers of zinc terephthalate bridged by **dabco**. Overall, catalytic quantities of salt additives exhibited a twofold effect on the milling reactions, by both increasing conversion to the MOF and affecting the polymorphic outcome of the reaction, possibly by anion templating. Inclusion of the salt additives in the final MOF was confirmed by solid‐state NMR spectroscopy on ^15^N‐labeled salt additives.

**Figure 5:**
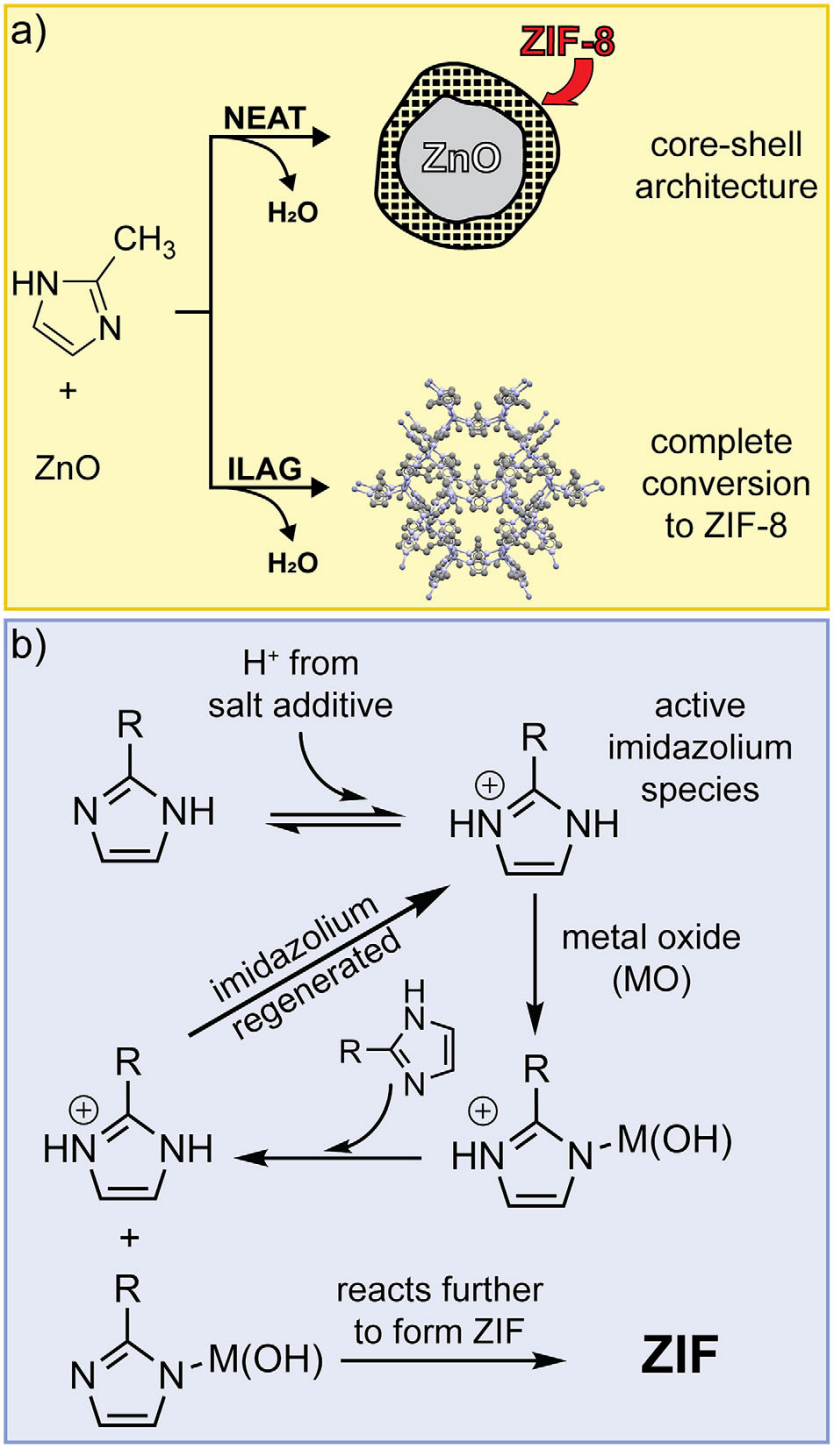
Schematic diagrams of: a) the outcomes of neat^[^ ^24^ ^]^ and ILAG synthesis of ZIF‐8 from ZnO^[^ ^58^ ^]^ and b) the proposed mechanism behind using protonated ammonium salts to facilitate the conversion of ZnO into ZIFs via ILAG.^[^ ^62^ ^]^

The ILAG methodology also enabled facile mechanochemical synthesis of metal azolate frameworks, notably zeolitic imidazolate framework (ZIF) materials directly from ZnO (**Figure** **5**a).^[^ ^58^ ^]^ Whereas brief (30 min) milling of ZnO with H**Meim** under neat or LAG conditions did not lead to complete formation of target ZIF‐8, ILAG with ammonium salt additives NH_4_NO_3_, NH_4_CH_3_SO_3_ or (NH_4_)_2_SO_4_ enabled improved or complete conversions. Replacing H**Meim** with 2‐ethylimidazole (H**Etim**) yielded polymorphic 3D Zn(**Etim**)_2_ frameworks with zeolite‐*ρ* (RHO), analcime (ANA), and quartz (*qtz*) topologies. Investigation of the reaction in a step‐by‐step fashion, as well as in subsequent real‐time studies,^[^ ^59^ ^]^ suggested that the obtention of different MOF polymorphs was not necessarily related to different topologies being stabilized by different salts, but could be related to differences in the reaction kinetics when using different salt additives. The ILAG methodology was subsequently used by Bin Zulkifli and co‐workers for the mechanochemical synthesis of nanoparticulate ZIF‐7 material from ZnO and benzimidazole in the presence or zinc acetate or NH_4_NO_3_,^[^ ^60^ ^]^ while Li and co‐workers^[^ ^61^ ^]^ have used ILAG of ZnO in the presence of zinc acetate to enable the synthesis of amine‐functionalized ZIF‐UC‐6 from ZnO, 2‐aminobenzimidazole and imidazole.

In the case of ZIF synthesis by ILAG from ZnO, the use of ammonium salts is critical: the NH_4_^+^ ions have been proposed to protonate imidazole ligand precursors, initiating an acid‐catalyzed process involving cycling of an active imidazolium species (Figure 5b). The involvement of imidazolium cations was supported by the observation that aging ammonium sulfate and imidazole (H**Im**) in moist air led to the formation of crystalline imidazolium sulfate (**H_2_Im**)_2_SO_4_·2H_2_O.^[^ ^62^ ^]^

Besides activation of zinc oxide, the ILAG methodology was recently used by Rodríguez‐Sánchez and co‐workers for the synthesis of a photocatalytically active Co^II^‐based ZIF material, ZIF‐9. The mechanochemical synthesis, which involved Co(OH)_2_, benzimidazole, and NH_4_Cl as the salt additive generated within 50 min a material that showed higher levels of catalytic activity compared with the material obtained through 48 h of solvothermal synthesis from Co(NO_3_)_2_·6H_2_O and benzimidazole in DMF.^[^ ^63^ ^]^

#### 2.2.4 Salt‐Assisted Grinding

Besides being used in catalytic amounts to activate a metal oxide during ILAG, simple inorganic salts such as NaCl or KCl can also be used in larger quantities as a means to modify the nature of the MOF product. An example of such salt‐assisted grinding was presented by the Yuan group,^[^ ^64^ ^]^ who used NaCl or KCl as a “solid solvent” for dispersing the metal precursor Cu(OAc)_2_·H_2_O and the H_3_ **btc** linker precursors before mechanochemical reaction to form the HKUST‐1 framework. The use of such salt diluent enabled the synthesis of HKUST‐1 samples exhibiting controllable mesoporosity, suitable for the adsorption of iodine‐based species such as CH_3_I. The methodology was further explored by Steenhaut and co‐workers,^[^ ^65^ ^]^ who compared the properties and amounts of defects in HKUST‐1 materials produced by salt‐assisted grinding, by LAG, and made solvothermally. The comparative study revealed that, after synthesis and washing, LAG‐based samples exhibited the lowest number of defects, compared to the MOFs made solvothermally or by salt‐assisted grinding which could contain significant amount of Cu^I^ defect sites.

The use of simple salts can be used to modify other types of solventless MOF syntheses. As an example, Gu and co‐workers have reported that the addition of NaCl to neat mixtures of H_2_ **ta** and ZrOCl_2_·8H_2_O followed by heating to 130 °C produces UiO‐66 materials with improved crystallinity and surface areas after activation compared to reactions conducted without the salt additive.^[^ ^66^ ^]^ In the context of coordination polymers, mechanochemical reactions with simple salts have been reported to lead to post‐synthetic anion exchange.^[^ ^18b^ ^]^

### 2.3 Resonant Acoustic Mixing (RAM) and Other Media‐Free Approaches to Mechanosynthesis

**Figure 6:**
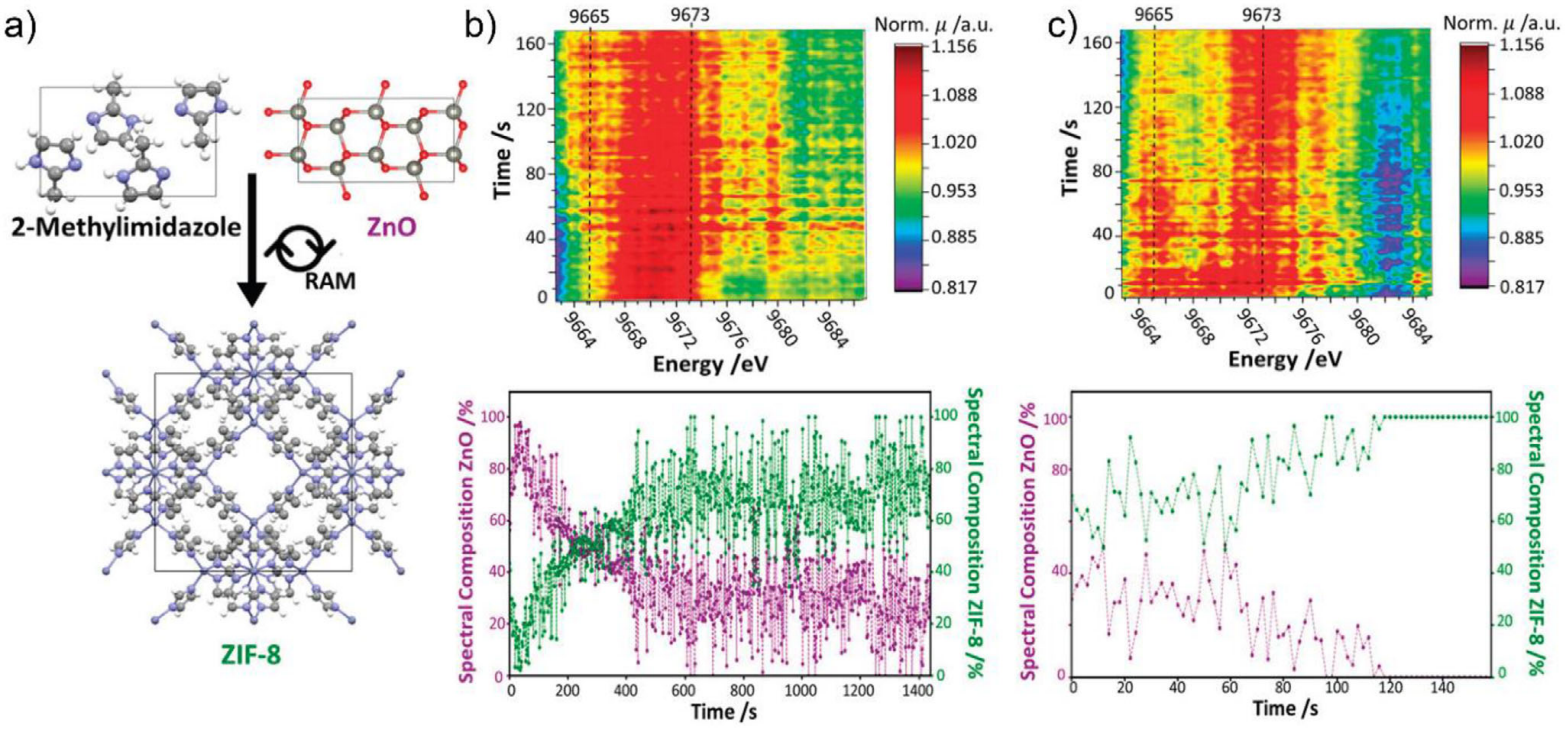
a) Scheme of the RAM reaction of H**Meim** and ZnO to form ZIF‐8, along with time‐resolved XAS data (top) and results of least‐squares analysis (bottom) for the reaction conducted: b) without any milling media and c) in the presence of zirconia balls. Reproduced under the terms of the CC‐BY license.^[^ ^70^ ^]^

Resonant acoustic mixing (RAM)^[^ ^67^ ^]^ is a mixing method that emerged recently as a means to induce mechanochemical transformations through acoustic‐frequency oscillations of the entire reaction mixture. Initial applications of RAM in mechanosynthesis focused on the formation of pharmaceutical and energetic cocrystals, with recent applications also including catalytic organic synthesis and covalent surface modification. In 2020, Titi et al. reported the synthesis of several MOFs, including ZIFs and HKUST‐1, using RAM technology.^[^ ^12^ ^]^ Adapting the ILAG technique, the synthesis of ZIF‐8 was accomplished by exposing a mixture of ZnO, H**Meim**, MeOH as a liquid additive and catalytic amounts of NH_4_NO_3_ (5 mol%) to acoustic oscillations of ca. 60 Hz and a maximum acceleration of 95 *g* (*g* = 9.8 m/s^2^) for 1 h (**Figure** **6**a), and the product displayed a high surface area (after washing and activation). Switching the zinc precursor to hydrated zinc nitrate, Zn(NO_3_)_2_·6H_2_O, in the presence of excess H**Meim** produced the 2D material ZIF‐L,^[^ ^68^ ^]^ a method that was further adapted to the synthesis of the cobalt‐based ZIF‐L‐Co by using the corresponding cobalt salt. Similarly, Cu^II^‐based HKUST‐1 was obtained by RAM of Cu(OAc)_2_·H_2_O and H_3_ **btc**.^[^ ^12^ ^]^ Whereas the synthesis of HKUST‐1 by neat ball milling proceeded within minutes, no reaction occurred by neat RAM. Addition of small amounts of water, however, enabled conversion to HKUST‐1.

The lack of milling media (i.e. milling balls) makes the RAM technology, in principle, more readily scalable compared with media‐based grinding technologies,^[^ ^69^ ^]^ as illustrated by the RAM‐based synthesis of ZIF‐L at 25‐gram scale.^[^ ^12^ ^]^ RAM is also ideally suited for in situ monitoring of reactions. For example, Buzanich et al. studied the mechanism of ZIF‐8 formation under RAM conditions by in situ synchrotron X‐ray absorption spectroscopy (XAS) (Figure 6b,c), which revealed continuous, albeit incomplete, formation of ZIF‐8 at 60 *g* acceleration.^[^ ^70^ ^]^

Another recently presented approach for the mechanochemical preparation of MOFs without milling or grinding media is air‐flow impacting,^[^ ^71^ ^]^ a method where reactant particles are accelerated to supersonic velocities before impinging in a solid target. The impact against the target was proposed to lead to a chemical reaction, as described by Guo et al. who reported the synthesis of HKUST‐1 and a related Cd^II^‐based trimesate framework by AFI of H_3_ **btc** with Cu(OAc)_2_ and Cd(OAc)_2_·2H_2_O, respectively.^[^ ^71^ ^]^

### 2.4 Continuous Mechanosynthesis By Twin‐Screw Extrusion (TSE)

While vibratory ball milling remains the most popular method for the mechanochemical synthesis of MOF materials, other grinding and agitation methods have been explored, particularly in the context of scale‐up. Twin‐screw extrusion (TSE)^[^ ^13^ ^]^ is a mixing technique where solids or pastes are confined between meshed screws and conveyed, compressed, and sheared by their rotation. The method has been used widely in the food, polymer and pharmaceutical industries, the primary attractive feature of TSE for large‐scale and industrial production being that it is a continuous process. The method is also highly tunable since factors like reaction temperature, screw speed, screw length, feed rate, and screw design all have a significant effect on the outcome of the process and are easily controlled. Such fine control makes TSE a compelling choice for continuous and large‐scale synthesis of MOFs.^[^ ^72^ ^]^

The James group pioneered the use of TSE for MOF synthesis, showing that HKUST‐1, ZIF‐8, and the fumarate (**fum^2−^**) based Al(**fum**)(OH) MOFs could be prepared by neat or liquid‐assisted TSE at rates of up to 1 kg/h.^[^ ^72^ ^]^ Importantly, the materials produced by TSE exhibited adsorption properties comparable to literature ones. Specifically, HKUST‐1 was obtained at room temperature by TSE in the presence of MeOH liquid additive, while the synthesis of ZIF‐8 and Al(**fum**)(OH) required higher temperatures. An efficient synthesis of ZIF‐8 was also accomplished at a rate of up to 70 g/min (4 kg/h), at 200 °C, by single‐screw extrusion (SSE), a related technology that relies on a changing the groove diameter of a single conveying screw.^[^ ^73^ ^]^ Another example of scalable, continuous MOF synthesis by TSE was reported by Karadeniz et al. who made UiO‐66‐NH_2_ from a dodecanuclear zirconium acetate cluster in the presence of a small amount of water, producing >100 grams of the material at a rate of ≈1.4 kg/h.^[^ ^74^ ^]^

By comparing ball‐milling and TSE for the synthesis of coordination polymers from the **bipy** linker and hydrated nitrate salts of Co^2+^, Zn^2+^, Ni^2+^, Cu^2+^ and Cd^2+^, the Zaworotko group found that both methods resulted in the same products,^[^ ^75^ ^]^ whereas analogous water slurry‐based experiments were globally ineffective. A different behavior was observed for the synthesis of the chain‐based coordination polymer derived from cobalt(II) thiocyanate, where water slurry was effective in providing the target material, as were ball‐milling and TSE in the presence of water additive.^[^ ^76^ ^]^ The prospect that vibratory ball‐milling protocols for MOF synthesis can be easily adapted to TSE is an attractive one, since the latter could provide the continuous large‐scale synthesis of materials whose preparation is first investigated on the more accessible ball mill. Such a workflow could avoid unnecessary waste associated with materials discovery and reaction optimization by TSE alone, as that particular methodology generally requires larger quantities of reactants for operation. Moreover, TSE has also been applied in the fabrication of hybrid composites of MOFs. For example, Richardson and coworkers reported a sustainable and scalable integration of cellulose nanocrystals (CNCs) and ZIF‐8,^[^ ^77^ ^]^ resulting in composites that combined CNCs' biocompatibility with MOFs' high surface area and tunability. They developed a room‐temperature, one‐step biomineralization process by TSE that enabled the rapid nucleation and growth of MOFs on CNC surfaces. As shown by scanning electron microscopy (SEM), the MOF nucleation occurred directly on the CNC surfaces, embedding them into the porous structure and forming core–shell architectures. This method produced CNC‐MOF filaments with customized geometries and structures without requiring pre‐formed MOFs or high temperatures, making it attractive for the development of functional, versatile, and environmentally friendly hybrid composites and significantly broadening the application scope of these hybrid materials.

TSE can also compound MOF formation and polymer blending, as shown by Quan et al. in a synthesis of MOF‐polymer pellets from zeolitic imidazolate frameworks (ZIF‐8 and ZIF‐67) and polymers such as polypropylene (PP) and polystyrene (PS).^[^ ^78^ ^]^ Compared with traditional solvothermal synthesis, this continuous, solvent‐free process achieved a remarkable production rate of ca. 1.2 × 10⁵ kg/day, significantly reducing the manufacturing time of the MOF‐polymer nanocomposites, while eliminating solvent use and the need for multiple unit operations, such as purification and drying. The resulting nanocomposites exhibited intact MOF structure, improved thermal stability, reduced flammability, and enhanced mechanical properties, including increasing stiffness without compromising tensile strength. Furthermore, the polymer‐ZIF‐67 nanocomposite films showed exceptional catalytic activity in the peroxymonosulfate‐based degradation of methylene blue, achieving over 90% degradation of the dye within 25 min while offering excellent recovery and reusability, making them potentially suitable for wastewater treatment.

### 2.5 Aging Reactions

**Figure 7:**
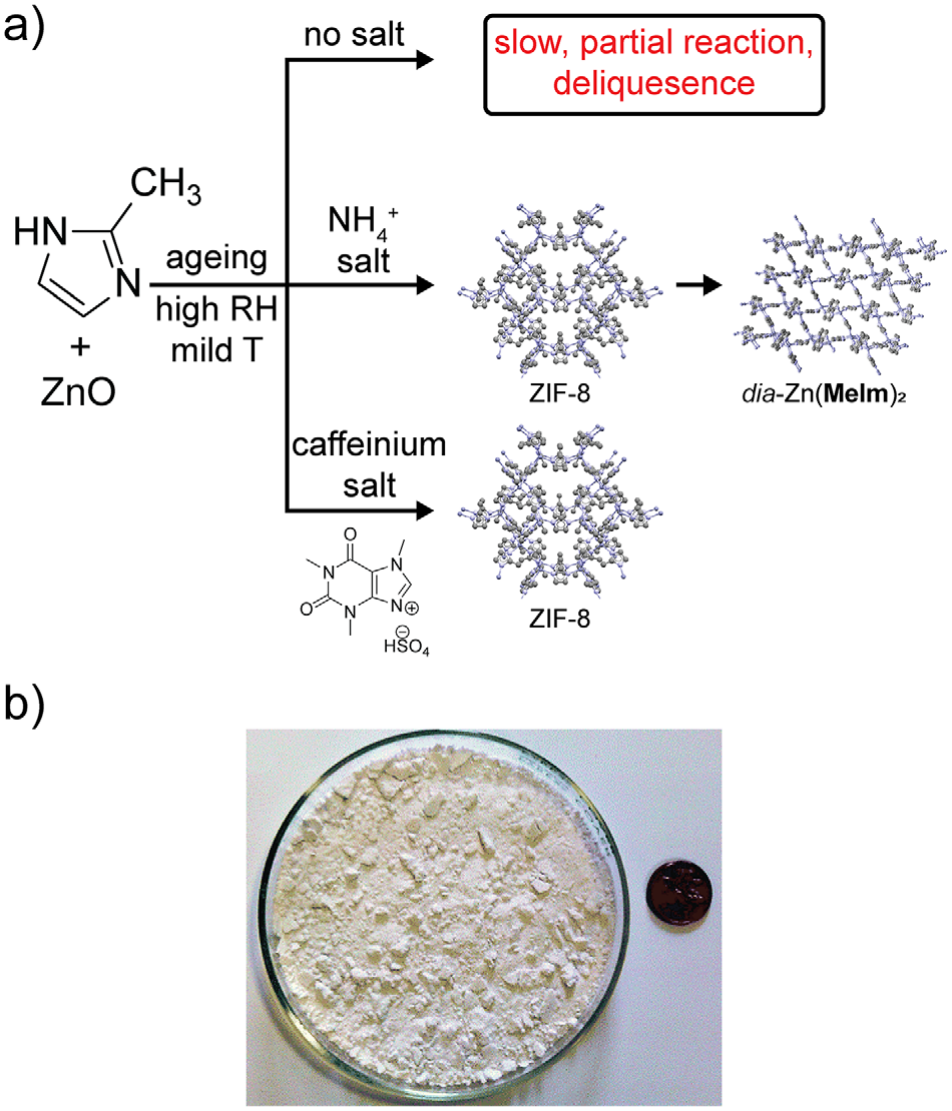
Synthesis of Zn(**Meim**)_2_ materials by aging.^[^ ^62^, ^83^ ^]^ a) Schematic of the mechanical pre‐activation and aging synthesis of ZIF‐8 and *dia*‐Zn(**Meim**)_2_. b) Picture of 5 g of ZIF‐8 synthesized by accelerated aging in the presence of caffeinium sulfate, upon exposure to 100% RH and 45 °C. Panel b) reproduced with permission.^[^ ^62^ ^]^ Copyright 2013, The Royal Society of Chemistry.

Chemical reactions leading to the formation of open framework materials can also be conducted by a brief mechanical mixing or grinding followed by aging of the resulting mixture in water or solvent vapor under mild conditions. These accelerated aging processes,^[^ ^79^ ^]^ whose synthetic advantages have been recognized by the Braga group in the context of organometallic cocrystal formation,^[^ ^80^ ^]^ provide access to metal–organic materials directly from small organic molecules and mineral‐like feedstocks such as metal oxides. In that way, accelerated aging mimics geological processes of mineral neogenesis, known to lead to the formation of 1D coordination polymer‐based minerals such as oxalate‐based Mooloite or Lindbergite in nature.^[^ ^81^ ^]^ Directing such aging processes toward the synthesis of more complex 2D or 3D frameworks was made possible by using organoammonium salts to template the formation of Co^II^‐, Ni^II^‐ and Zn^II^‐based oxalate frameworks from the corresponding oxides. This effect also suggests a possible templated route for the formation of minerals such as stepanovite and zhemchuzhnikovite that exhibit 2D MOF structures.^[^ ^81^, ^82^ ^]^ The accelerated aging protocols were readily adapted to the synthesis of functional MOF materials, such as ZIFs.^[^ ^62^, ^83^ ^]^ Synthesis of ZIFs by accelerated aging can be promoted by protic catalysts, similarly to ILAG reactions: while ground mixtures of ZnO with H**Im**, H**Meim**, or H**Etim** reacted slowly upon exposure to high relative humidity (RH) at 45 °C, aging in the presence of simple ammonium (NH_4_^+^) salts led to complete, or nearly complete, formation of dense‐framework ZIF materials via the intermediacy of open‐framework MOFs such as ZIF‐8. The formation of open ZIFs, such as SOD‐topology Zn(**Meim**)_2_ and Co(**Meim**)_2_ (ZIF‐8, ZIF‐67, respectively) or RHO‐Zn(**Etim**)_2_, through accelerated aging from corresponding metal oxides (i.e., ZnO, CoO) required other types of protic catalysts, notably KHSO_4_ or protonated imidazolium salts (**Figure** **7**). Caffeinium hydrogen sulfate [H**caf** ^+^][HSO_4_^−^] was found to be a particularly effective catalytic additive for the synthesis of ZIF‐8 and Zn(**Etim**)_2_, enabling quantitative syntheses over a period of 2–4 days depending on the reaction scale. Whereas the accelerated aging conversion of CoO to ZIF‐67 did not reach more than ca. 80% after 3 days, such a result compared well against the lower conversions obtained by heating over similar periods of time. Aging also provides effective access to MOFs with complex nodes, as illustrated by the synthesis of zirconium‐based UiO‐66 through exposing a mixture of terephthalic acid (H_2_ **ta**) and a pre‐assembled carboxylate‐capped hexanuclear zirconium cluster over three days to MeOH vapor at 45 °C.^[^ ^84^ ^]^

Despite longer reaction times, the aging protocols provide an attractive alternative to mechanochemical or solution protocols, as they enable conversion of highly stable metal oxide precursors while minimizing energy consumption. This was illustrated by Yuan et al.^[^ ^85^ ^]^ for the synthesis of HKUST‐1 directly from CuO, as well as for the synthesis of diverse trimesate‐based lanthanide(III) MOFs directly from corresponding sesquioxides. None of these processes appeared to take place by simple milling.

Accelerated aging also shows potential for MOF discovery, as illustrated by Brekalo et al. who obtained the previously unreported RHO‐topology Zn(**Im**)_2_ framework by accelerated aging of ZnO and H**Im** in the presence of a Cram's macrocyclic cavitand as template.^[^ ^86^ ^]^ The formation of RHO‐Zn(**Im**)_2_ was rationalized by an excellent shape match and C‐H···O recognition between the double 8‐rings of the RHO‐framework and the cavitand. Notably, attempts to use the macrocycle as a template in solution synthesis were generally low yielding, required large excess of the macrocycle, and produced only MOFs with the known dense TSC‐topology. Further information on aging reactions for the synthesis of MOFs, as well as other types of transformations, can be found in relevant reviews.^[^ ^79^ ^]^

## 3 Overview of Mechanochemically Prepared MOF Materials

### 3.1 Carboxylate‐Based MOFs

Among the earliest entries of mechanochemistry for the synthesis of archetypal open‐framework carboxylate MOFs have been the syntheses of the copper‐based HKUST‐1 material by the Emmerling and the James groups. The synthesis of HKUST‐1 was performed by LAG of copper(II) acetate with H_3_ **btc** in the presence of a small amount of EtOH^[^ ^87^ ^]^ or MeOH^[^ ^22^ ^]^ in 15–25 min. Similarly, MOF‐14 was synthesized by substituting benzenetribenzoic acid (H_3_ **btb**) for H_3_ **btc**.^[^ ^87^ ^]^ For HKUST‐1, washing the material with EtOH^[^ ^22^ ^]^ prior to activation was essential to obtain surface areas comparable with those reported in the literature for materials synthesized through solvothermal routes.

The Materiaux de l'Institut Lavoisier (MIL)^[^ ^88^ ^]^ class of MOFs constitutes one of the archetypal examples of microporous metal–organic materials that can be accessed mechanochemically, although their direct mechanochemical syntheses have not been explored as much as for other popular materials such as ZIFs. A number of reports outline the synthesis of MIL‐type materials through a combination of grinding and high‐temperature treatment.^[^ ^8^ ^]^ For example, chromium‐based MIL‐53‐Cr was synthesized by grinding a Cr^3+^ salt and H_2_ **ta** in neat conditions, followed by high‐temperature treatment (>200 °C).^[^ ^89^ ^]^ A fully mechanochemical synthesis of MIL‐53‐Al was recently reported by Salvador and co‐workers, by LAG with DMF of stoichiometric amounts of Al_2_(SO_4_)_3_·18H_2_O, H_2_ **ta,** and NaOH.^[^ ^90^ ^]^ Remarkably, the use of other Al^3+^ sources, such as Al(OH)_3_, Al(OH)(OAc)_2_, or Al(NO_3_)_3_·9H_2_O did not yield the targeted product. When the MOFs were synthesized by milling all reagents in a one‐pot strategy, their microporous surface areas were lower than those of solvothermally made materials. To obtain congruent microporous surface areas, the synthesis needed to be conducted in a stepwise fashion, by milling H_2_ **ta** and NaOH first, followed by the addition of Al_2_(SO_4_)_3_·18H_2_O. The strategy was readily applicable to other isoreticular analogs of MIL‐53‐Al, based on fumaric (H_2_ **fa**), methylfumaric acid (H_2_ **Mefa**), 2‐aminoterephthalic acid (H_2_ **ata**) and bis‐2,5‐trifluoromethylterephthalic acid (H_2_ **btta**), as well as to Ga‐ and In‐based analogs of MIL‐53‐Al.

### 3.2 MOFs Based on Metal Oxo‐Clusters

**Figure 8:**
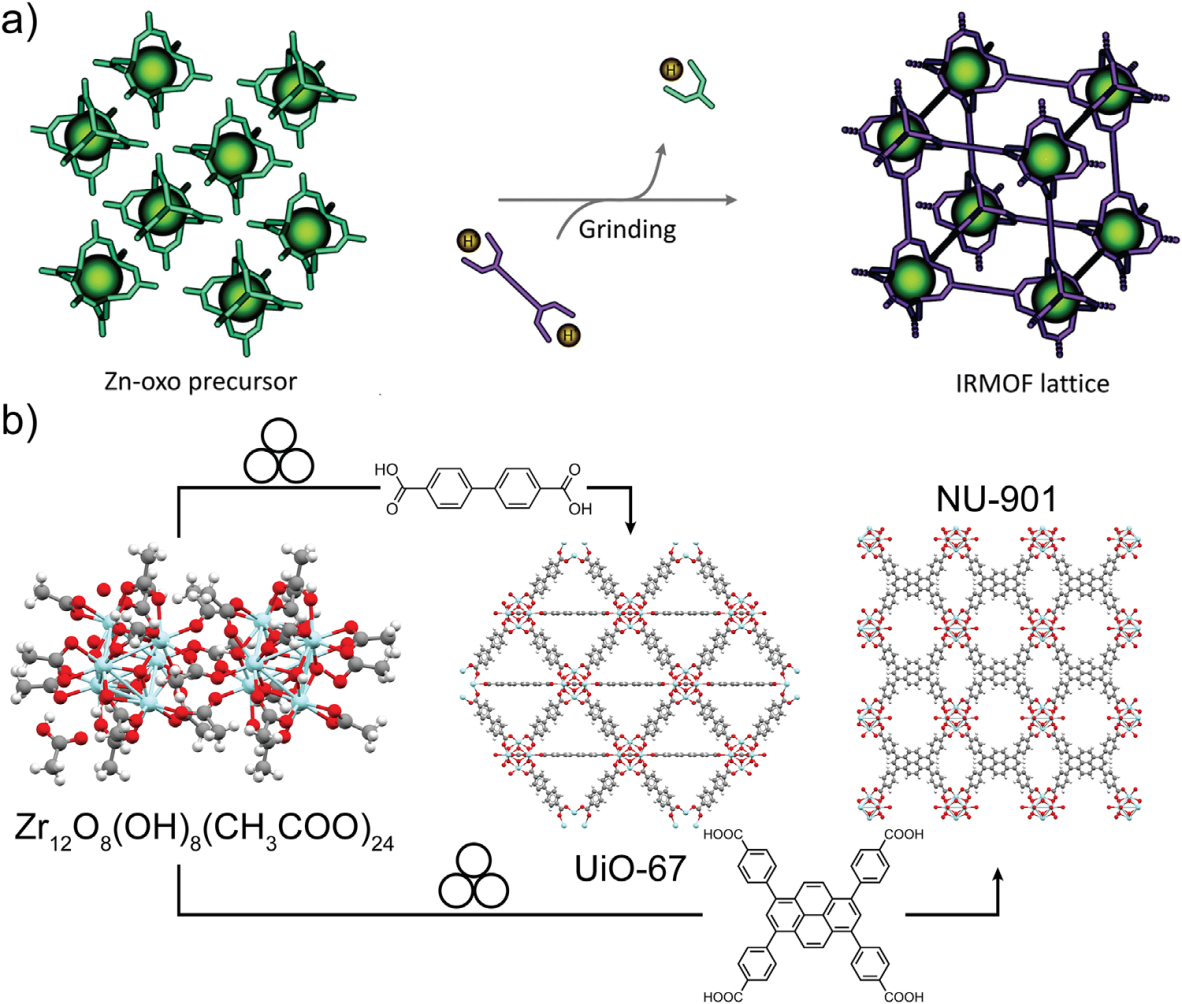
Schematic representation of the mechanochemical syntheses of MOFs based on zirconium‐oxo clusters. a) The “SMART” approach reported by the Lewiński group, where metal‐oxo clusters are pre‐synthesized before their mechanochemical reaction with organic linkers to form a MOF. b) The synthesis of the UiO‐67 and NU‐901 MOFs by ball‐milling a pre‐synthesized Zr_12_ acetate oxo‐cluster with H_2_bpa and H_4_tbapy, respectively. Panel b) reproduced with permission.^[^ ^92^ ^]^ Copyright 2015, The Royal Society of Chemistry.

One of the most iconic materials in the development of MOFs is MOF‐5,^[^ ^91^ ^]^ which comprises oxide‐centered [Zn_4_O]^6+^ octahedral nodes (Zn_4_O clusters) bridged by terephthalate anions. Attempts to synthesize MOF‐5 by ball‐milling the oxide‐containing ZnO and H_2_ **ta** under various conditions generally led to zinc terephthalate coordination polymers, highlighting the difficulty to mechanochemically generate the underlying [Zn_4_O]^6+^ node. A means to overcome this challenge was presented by the Lewiński group,^[^ ^92^ ^]^ who generated MOF‐5 and related isoreticular MOFs by employing a pre‐assembled Zn_4_O‐cluster core unit (**Figure** **8**a). The strategy, termed SBU‐based Mechanochemical Approach for pRecursor Transformation (SMART, with SBU meaning Secondary Building Unit),^[^ ^92^ ^]^ was successful at generating MOF‐5 when H_2_ **ta** and a pre‐assembled discrete carboxylate‐capped Zn_4_O‐cluster are milled with *N,N*‐diethylformamide (DEF) as liquid additive, albeit with incomplete conversion. Replacing the carboxylate‐based cluster with the corresponding amidate‐based cluster, however, enabled a rapid synthesis of a MOF‐5 material with a high surface area, a success ascribed to the higher basicity of the amidate capping ligand. Following the synthesis of MOF‐5, the amidate cluster‐based SMART approach was also applied to the synthesis of other MOFs based on the Zn_4_O unit and longer, more complex linkers, including biphenyl‐based MOF‐10 and benzene‐1,3,5‐tribenzoic acid‐based MOF‐177.^[^ ^93^ ^]^ Finally, two routes to MOF‐5 without using a pre‐assembled Zn_4_O‐cluster were reported:^[^ ^94^ ^]^ the Morsali group described the synthesis of MOF‐5 and several related isoreticular frameworks by manual grinding in air of zinc acetate dihydrate, Zn(OAc)_2_·2H_2_O, with H_2_ **ta** in the presence of DMF,^[^ ^94a^ ^]^ while Lv et al.^[^ ^94b^ ^]^ obtained MOF‐5 by milling an excess of Zn(OAc)_2_·2H_2_O with H_2_ **ta**, followed by suspending the product in DMF. In the latter, the Zn:H_2_ **ta** ratio as well as immersion in DMF were critical to obtain the crystalline MOF‐5 structure, as evidenced by PXRD analyses.

A variation of the SMART strategy, based on using pre‐assembled oxo‐clusters as starting materials, was subsequently applied to synthesize zirconium‐based MOFs containing the 12‐coordinated Zr_6_O_4_(OH)_4_^12+^ clusters (Zr_6_‐clusters) as nodes.^[^ ^84^ ^]^ Such MOFs, as illustrated by the terephthalate‐based UiO‐66, are recognized as particularly robust materials with high surface areas and have therefore been an attractive target for mechanosynthesis. A strategy for the synthesis of UiO‐66 and its derivative based on 2‐aminoterephtalic acid (UiO‐66‐NH_2_) was reported by Užarevic et al. by ball‐milling of the pre‐synthesized methacrylate‐capped oxo‐zirconium cluster Zr_6_O_4_(OH)_4_(C_2_H_3_CO_2_)_12_ in the presence of MeOH, producing MOFs of high crystallinity and surface area.^[^ ^84^ ^]^ In contrast, using DMF as a liquid additive or using the analogous benzoate‐capped cluster precursor gave MOFs of lower crystallinity and poor surface areas, highlighting the importance of a judicious selection of cluster‐capping ligands and liquid additives when planning the mechanosynthesis of UiO‐type MOFs. The approach was readily scaled up to multi‐gram amounts. The catalytic activity of mechanochemically produced UiO‐66‐NH_2_ toward the degradation of a nerve agent simulant was reported to be comparable to that of analogous materials prepared solvothermally, demonstrating that mechanochemically‐synthesized materials can be as reactive as their solution‐made counterparts.

The mechanochemical synthesis of Zr_6_‐based MOFs was expanded by Fidelli et al. to materials involving large carboxylate‐based linkers, pyrene‐1,3,6,8‐tetrakis(*p*‐benzoic acid) (H_4_ **tbapy**) and biphenyl‐4,4′‐bis(carboxylic acid) (H_2_ **bpa**), yielding NU‐901 and UiO‐67, respectively (Figure 8b).^[^ ^45^ ^]^ Whereas milling of H_4_ **tbapy** with the benzoate‐capped Zr_6_‐cluster did not produce a MOF, using the methacrylate‐capped cluster yielded a sticky material with a moderate Brunauer‐Emmett‐Teller (BET) surface area (450‐900 m^2^g^−1^) and a PXRD pattern consistent with NU‐901. The hydrogen‐bonded Zr_12_‐cluster Zr_12_O_8_(OH)_8_(OAc)_24_, a self‐assembled supramolecular dimer of Zr_6_‐clusters, proved to be a much more versatile precursor for the synthesis of zirconium‐based MOFs. The use of this acetate‐based precursor together with H_4_ **tbapy** produced a free‐flowing powder of NU‐901, with a BET surface area after activation exceeding 1,600 m^2^/g, while in combination with H_2_ **bpa** the acetate‐based precursor afforded a highly crystalline UiO‐67 material with a BET surface area of 2,250 m^2^g^−1^. In contrast, using the methacrylate‐capped Zr_6_‐cluster as a precursor yielded a UiO‐67 material with significantly lower surface areas (750 m^2^g^−1^; 380–1165 m^2^ g^−1^).^[^ ^45^ ^]^

An analogous strategy was subsequently applied by Salvador et al. for the synthesis of Hf‐based analogs of UiO‐66, UiO‐66‐NH_2_, and UiO‐67 by utilizing a dodecanuclear acetate‐based hafnium cluster, Hf_12_O_8_(OH)_8_(OAc)_24_, analogous to the aforementioned Zr_12_‐cluster.^[^ ^95^ ^]^ Moreover, using mixtures of the Zr_12_‐ and Hf_12_ ‐clusters enabled syntheses of mixed‐metal UiO‐66 materials, in which the Zr:Hf content closely matched the stoichiometric ratio of the two reacting clusters.

Mechanosynthesis of UiO‐67 was also reported by the Bantreil group,^[^ ^96^ ^]^ who exploited LAG of H_2_ **bpa** with methacrylate‐ or benzoate‐capped Zr_6_‐cluster precursors, with the former in combination with DMF as liquid additive providing the material in highest purity. The optimized protocol was subsequently applied to 2,2‐dipyridyl‐5,5’‐dicarboxylic acid, producing a MOF isostructural to UiO‐66, but with accessible nitrogen‐based chelating sites on the linkers. The MOF was subsequently milled with CuBr_2_ leading to incorporation of Cu^2+^ at the chelating sites, in that way providing an example of a two‐step mechanochemical MOF synthesis of a bimetallic system made via mechanochemical post‐synthetic modification.

### 3.3 Mechanochemical In Situ Assembly of Complex Nodes

**Figure 9:**
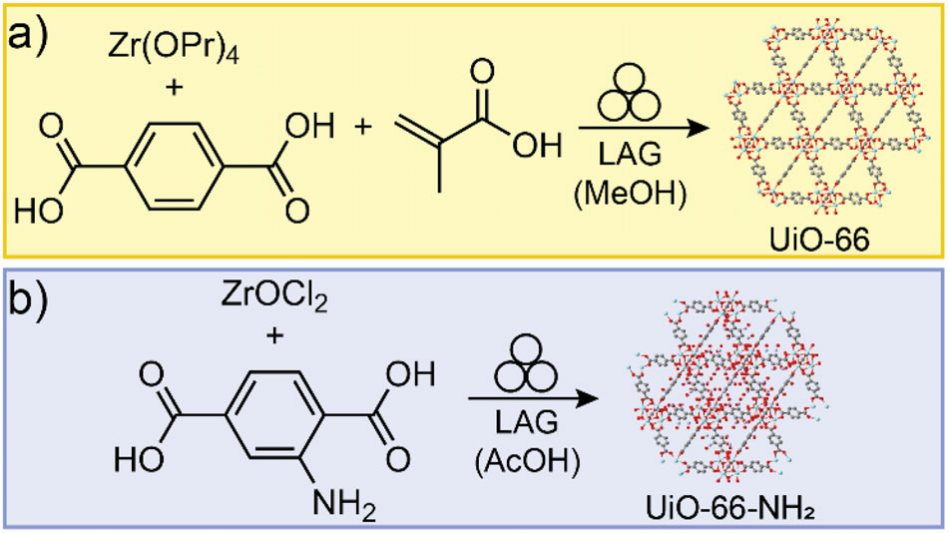
Mechanochemical synthesis of MOFs based on metal‐oxo clusters directly from simple zirconium sources. a) Synthesis of UiO‐66 from Zr(OPr)_4_ and H_2_ **ta** with methacrylic acid and MeOH additives.^[^ ^84^ ^]^ b) Synthesis of UiO‐66‐NH_2_ from ZrOCl_2_ and H_2_ **ata** with acetic acid as a liquid additive.^[^ ^51^ ^]^

In a departure from the SMART strategy, which requires the use of pre‐assembled oxo‐clusters as starting materials, Užarević et al. outlined that the synthesis of UiO‐66 was also possible by ball‐milling directly from commercially available zirconium(IV) isopropoxide, demonstrating one‐pot assembly of the required Zr_6_‐clusters, as well as their assembly into a 3D open MOF, mediated by the presence of methacrylic acid (**Figure** **9**a).^[^ ^84^ ^]^ The possibility to assemble the Zr_6_‐clusters in situ for mechanochemical MOF formation was also demonstrated by the D'Alessandro group (Figure 9b).^[^ ^51^ ^]^ Specifically, ball‐milling of ZrOCl_2_·8H_2_O with 2‐aminoterephthalic acid (H_2_ **ata**) in the presence of glacial acetic acid as liquid additive (*η* ∼ 0.17 µL/mg) at a ca. 3.5‐gram total scale led to the formation of UiO‐66‐NH_2_ within 90 min.

The ability to assemble the required complex metal nodes in situ is a highly attractive opportunity for large‐scale manufacturing and achieving the competitive placement of MOF materials on the market. In this context, an interesting approach for the production of MOFs was outlined by He et al. as a two‐step methodology for mechanochemically converting terephthalic acid from waste polyethylene terephthalate (PET) into MOFs.^[^ ^97^ ^]^ The first step converts the PET polymer into a mixture of sodium terephtalate (Na_2_ **ta**) and ethylene glycol upon milling with sodium hydroxide (NaOH). In the second step, milling of the reaction mixture with the addition of metal nitrate or chloride salts led to the formation of carboxylate‐based MOFs of Mn^II^, Co^II^, Ni^II^, Ca^II^ and La^III^. Notably, using ZrCl_4_ as metal source yielded a material with a PXRD pattern consistent with UiO‐66 (albeit with poor crystallinity), indicating that mechanochemical treatment can both depolymerize PET and assemble the Zr_6_‐cluster node. The potential for scale‐up was demonstrated with a Ni^II^‐based MOF as a model, providing 60 grams of material via planetary milling process that involved four sample milling stations.^[^ ^97^ ^]^

### 3.4 Mechanochemical In Situ Assembly of Linkers

**Figure 10:**
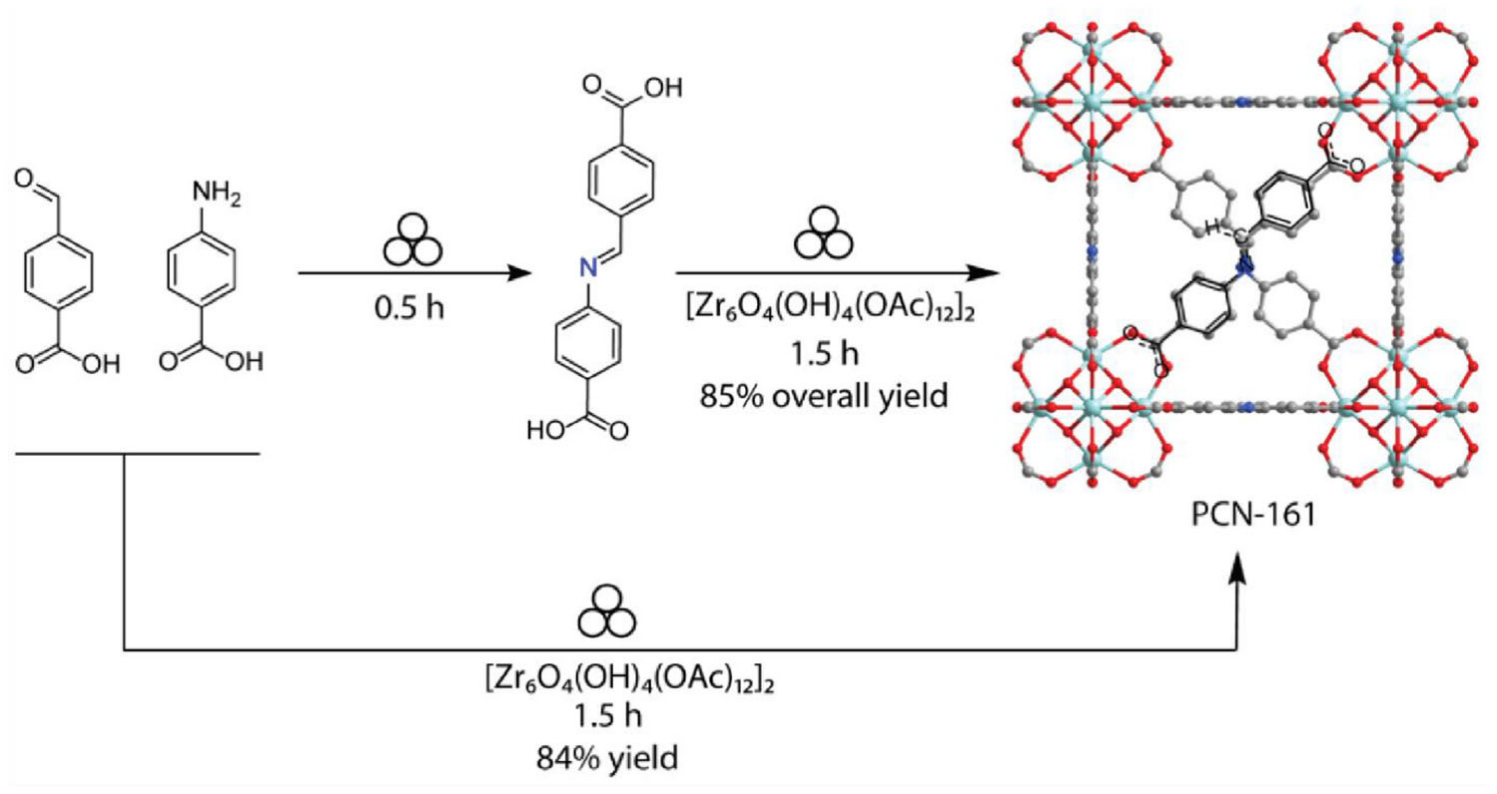
Mechanochemical synthesis of the zirconium MOF PCN‐161 based on imine linkers, through either a multi‐component one‐pot, or a two‐step one‐pot ball‐milling strategy, reported by Tegudeer et al.^[^ ^98^ ^]^ Reproduced with permission.^[^ ^98^ ^]^ Copyright 2025, American Chemical Society.

A mechanochemical methodology to synthesize MOF based on linkers that are too sensitive to permit effective solvothermal synthesis was recently demonstrated by Tegudeer and co‐workers.^[^ ^98^ ^]^ Specifically, the synthesis of zirconium‐based MOFs based on Schiff base (imine) linkers is generally found to be a challenge due to the sensitivity of such linkers to harsh solvothermal reaction environments, requiring complex procedures such as linker exchange on pre‐synthesized MOF materials. A means to overcome this challenge was offered by ball‐milling mechanochemistry which permitted the synthesis of the target imine‐based zirconium MOF PCN‐161,^[^ ^98^ ^]^ either through direct one‐pot synthesis from the zirconium cluster precursor and a combination of amine and aldehyde reactants that condense to form the imine‐based linker, or through a two‐step one‐pot approach in which the imine linker is produced through ball milling in the first step, followed by LAG with the zirconium cluster precursor (**Figure** **10**). The overall yields in both cases were similar, around 85%. This work further highlights the effectiveness of mechanochemistry for the one‐pot multi‐component synthesis of complex products from simpler precursor, previously demonstrated for the synthesis of small‐molecule complexes.^[^ ^99^ ^]^

### 3.5 Zeolitic Imidazolate Frameworks (ZIFs)

In 2006, Fernández‐Bertrán et al. reported a systematic investigation of mechanochemical reactions by manual grinding of metal oxides with imidazole (H**Im**).^[^ ^100^ ^]^ Diverse metal precursors, including ZnO, HgO, Ag_2_O, and Cu_2_O, readily underwent reactions, whereas CdO, Ga_2_O_3_, and In_2_O_3_ reacted more slowly, and the highly basic oxides MgO, CaO, BaO, and Al_2_O_3_ did not react at all. Although the products of these manual grinding reactions remained largely unidentified, it is likely that at least some were ZIF‐like materials. An early approach to metal‐imidazolate frameworks by grinding was outlined by Adams et al. who employed grinding of preformed imidazole complexes of metal chlorides, M(H**Im**)_2_Cl_2_, where M = Co^II^, Ni^II^, Cu^II^, Zn^II^, with an external base such as KOH to generate non‐porous M(**Im**)_2_ framework materials alongside the KCl byproduct. This strategy produced *zni*‐topology Zn(**Im**)_2_ and Co(**Im**)_2_, as well as the *α*‐polymorph of Ni(**Im**)_2_ and the green polymorph of Cu(**Im**)_2_.^[^ ^101^ ^]^

**Figure 11:**
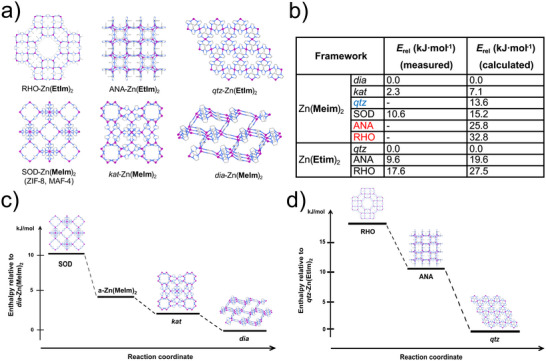
Thermodynamics of ZIF polymorphs.^[^ ^103^ ^]^ a) Mechanochemically obtained polymorphs of Zn(**Etim**)_2_ and Zn(**Meim**)_2_ frameworks and b) their associated measured and calculated energies, expressed relative to the most dense phase for each system. The *qtz*‐Zn(**Meim**)_2_ phase was anticipated to exist based on calculations and was recently discovered through amorphous phase crystallization. The experimentally determined stabilities show that the sequence of phases observed ex situ and in situ during mechanochemical synthesis of: c) Zn(**Meim**)_2_ and d) Zn(**Etim**)_2_ follows Ostwald's rule of stages. Reproduced with permission.^[^ ^103^ ^]^ Copyright 2017, American Chemical Society.

A targeted mechanochemical synthesis of ZIF materials, including open framework ones, was outlined in 2010 by Beldon et al.,^[^ ^58^ ^]^ who investigated the ball‐milling reactions of ZnO with H**Im**, H**Meim** and H**Etim** by neat grinding, LAG and ILAG, with the latter enabling rapid, simple synthesis of ZIFs within 30–60 min, using as a liquid additive DMF or the significantly greener MeOH or EtOH. The ability to selectively synthesize different polymorphs of Zn(**Meim**)_2_ or Zn(**Etim**)_2_ by varying the reaction time or the nature of liquid and ILAG salt additives was exploited to advance the understanding of thermodynamic stabilities of ZIFs and their polymorphs. Whereas significant attention is generally paid to understanding and controlling the kinetic behavior of MOF formation, little attention has been paid to the fundamentally important thermodynamic stability, which is also the driving force behind kinetic behaviors such as sensitivity to different gases or moisture.^[^ ^102^ ^]^ An assessment of the relative thermodynamic stabilities of ZIF polymorphs was reported by Akimbekov et al.,^[^ ^103^ ^]^ who used mechanochemistry to generate samples of RHO‐, ANA‐ and *qtz*‐Zn(**Etim**)_2_, SOD‐Zn(**Meim**)_2_ (i.e., ZIF‐8), *dia*‐Zn(**Meim**)_2_, an impure sample of the sensitive *kat*‐Zn(**Meim**)_2_ phase,^[^ ^104^ ^]^ and a sample of amorphous material a‐Zn(**Meim**)_2_, all suitable for dissolution calorimetry studies (**Figure** **11**a). The measured enthalpy of formation for samples of ZIF‐8 made solvothermally, by ball‐milling and by an accelerated aging procedure were all very similar, indicating that the structure and composition of the material is what dictates its thermodynamic stability, regardless of the synthesis method used. Moreover, the order of experimentally measured enthalpies coincides with the order of appearance of ZIF phases observed through real‐time monitoring of the mechanochemical reactions of ZnO with H**Meim** or H**Etim**. This indicated that these mechanochemical reactions follow the Ostwald's rule of stages,^[^ ^105^ ^]^ i.e., the transformation of the initially formed low‐stability product into increasingly more stable forms (Figure 11b–d). These experimental formation enthalpies have subsequently enabled the validation of computational approaches that calculate the relative energies of ZIF polymorphs. The experimental order of enthalpic stabilities of topologically different polymorphs of Zn(**Etim**)_2_ and Zn(**Meim**)_2_ was reproduced readily by periodic DFT calculations that include semi‐empirical dispersion correction (DFT‐SEDC calculations, Figure 11b).^[^ ^103^ ^]^ The dispersion correction was shown to be critical to obtain realistic energy differences.^[^ ^106^ ^]^ Although the periodic DFT‐SEDC calculations over‐estimated the relative energy gap between the close‐packed and open topologies, e.g. between *dia*‐Zn(**Meim**)_2_ and *kat*‐Zn(**Meim**)_2_, it accurately reflected the energy differences between polymorphs with more open topologies, *e.g*, between *kat*‐Zn(**Meim**)_2_ and SOD‐Zn(**Meim**)_2_.

The exploration of the mechanochemical synthesis of a Hg^II^‐based ZIF analogue by Speight and co‐workers has highlighted the importance of weak, non‐covalent interactions in determining the stability of MOF polymorphs.^[^ ^107^ ^]^ In particular, whereas mercury(II) imidazolate, Hg(**Im**)_2_, was reported to form a non‐porous interpenetrated *dia*‐topology framework with no other polymorphs observed,^[^ ^108^ ^]^ later attempts by our group to synthesize this material by mechanochemical ball‐milling of HgO and H**Im** produced a previously not reported 2D polymorph comprising stacked *sql*‐topology sheets. The same *sql*‐Hg(**Im**)_2_ phase was obtained by milling in the presence of different liquid additives, by accelerated aging synthesis at 45 °C and 100% RH over 18 h, as well as through solution synthesis. Nevertheless, a careful investigation of the milling and aging reactions at short reaction times, i.e. after 30 s for milling reactions or 90 min for aging reactions, revealed that the elusive *dia*‐Hg(**Im**)_2_ appears as a short‐lived intermediate in the formation of *sql*‐Hg(**Im**)_2_. The formation of a 2D material via a 3D connected intermediate in Hg(**Im**)_2_^[^ ^107^ ^]^ runs opposite to the conventional wisdom regarding framework stability, where dense 3D structures are anticipated to be the more stable ones. Periodic DFT‐SEDC calculations indicated that *sql*‐Hg(**Im**)_2_ was ca. 10 kJ/mol more stable than the interpenetrated *dia*‐polymorph, consistent with experimental observations. Calculations without SEDC, however, gave the opposite relationship, with *dia*‐Hg(**Im**)_2_ being almost 8 kJ/mol more stable than *sql*‐Hg(**Im**)_2_. These calculations emphasize the importance of weak, dispersion‐type interactions during the framework assembly process. Notably, the *sql*‐Hg(**Im**)_2_ structure exhibits short C‐H···Hg interactions, which are not present in *dia*‐Hg(**Im**)_2_, with contact distances ≈10% lower than the sum of the Van der Waals radii of H and Hg. This interaction, potentially of agostic character, highlights the complex interplay of the phenomena that determine the structures and properties of MOFs based on heavy metals. The difficulty in reproducing the originally reported, metastable *dia*‐Hg(**Im**)_2_ phase draws parallels to the disappearing polymorphs phenomenon of organic solid‐state chemistry,^[^ ^109^, ^110^ ^]^ and reinforces the need for careful tailoring of (mechano)synthetic conditions when designing large‐scale manufacturing of ZIFs and other MOFs capable of exhibiting polymorphism or supramolecular isomerism.^[^ ^111^, ^112^ ^]^

### 3.6 Database‐Driven Materials Discovery

**Figure 12:**
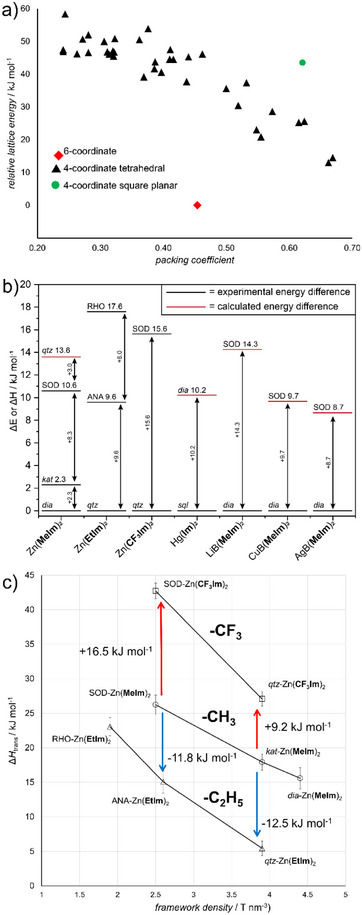
a) Calculated energy‐density landscape for putative zinc pentazolate frameworks generated using the database‐driven screening, showing the possibility of an open framework with a six‐coordinate node being more stable than denser material based on four‐coordinate nodes. b) Overview of the measured and/or calculated relative stabilities for different ZIF polymorphs, shown relative to the most stable dense phase. c) Relative stabilities of polymorphs of Zn(**Etim**)_2_, Zn(**Meim**)_2_ and Zn(**CF_3_Im**)_2_ systems shown as transition enthalpy with respect to framework density. Panel a) reproduced under the terms of the CC‐BY 3.0 license.^[^ ^113^ ^]^ Panel c) reproduced with permission.^[^ ^114^ ^]^ Copyright 2019, American Chemical Society.

The match of experimental formation enthalpies and DFT‐derived differences in stability of ZIF polymorphs was used to develop a database‐driven approach to ZIF discovery. The approach consisted of searching the Cambridge Structural Database (CSD) for all metal azolate structures, then theoretically evaluating the structures and relative stabilities of corresponding putative frameworks based on a given chemical composition. It was first applied to explore the structural landscape of ZIF materials that would be challenging to synthesize, notably framework materials based on the sensitive pentazolate anion (**pnz** ^−^), the all‐nitrogen analog of imidazolate.^[^ ^113^ ^]^ The CSD/DFT‐based investigation of the phase landscapes for Zn(**pnz**)_2_ and Cd(**pnz**)_2_ materials led to the discovery of two framework topologies previously not described and, importantly, revealed the possibility of open‐framework materials that occupy an energy minimum compared to other close‐packed variants (**Figure** **12**a).

The CSD‐driven ZIF discovery approach was subsequently validated experimentally by exploring the framework topology landscape for a previously not synthesized MOF, zinc(2‐trifluoromethylimidazolate), Zn(**CF_3_Im**)_2_, a trifluoromethylated analog of ZIF‐8 (Figure 12).^[^ ^114^ ^]^ The CSD‐driven structure prediction strategy yielded a landscape of topologically‐distinct structures that were ranked by energy, which indicated that the *qtz*‐topology structure should be the most stable one, followed by structures with *dia*‐ (at +13.5 kJ/mol), SOD‐ (at +15.9 kJ/mol) and *kat*‐ (at +27 kJ/mol) topologies, as well as other structures that were >30 kJ/mol higher in energy. The outcomes of the CSD‐driven prediction procedure were followed by attempted synthesis of Zn(**CF_3_Im**)_2_ by ball‐milling. Previous solution‐based attempts of SOD‐Zn(**CF_3_Im**)_2_ synthesis were reported to yield oxo‐bridged coordination polymers, and a SOD‐topology framework was only obtained in a mixed‐linker system with H**Meim**. In contrast, LAG of ZnO with 2‐trifluoromethylimidazole (H**CF_3_Im**) readily produced SOD‐Zn(**CF_3_Im**)_2_ which, upon further milling, transformed into the close‐packed *qtz*‐Zn(**CF_3_Im**)_2_. Both materials were accessed individually through optimization of reaction conditions, and, after PXRD structure analysis, were identified as the first and third lowest‐energy ranked structures in the CSD‐driven prediction. Calorimetric measurements revealed an excellent match between experimentally determined difference in stability of the *qtz*‐ and SOD‐forms (15.6 kJ mol^−1^) and the values calculated using the computation PBE+MBD* approach (15.9 kJ mol^−1^), with a somewhat poorer agreement when using the PBE+D2 approach (19.0 kJ mol^−1^) (Figure 11b). The observed initial formation of the SOD‐topology polymorph followed by transformation into the denser *qtz*‐topology phase is consistent with the behavior observed upon monitoring the synthesis of Zn(**Meim**)_2_ and Zn(**Etim**)_2_ materials.^[^ ^59^, ^104^, ^114^ ^]^

The potential of the CSD‐driven approach to predict novel ZIFs was further validated by the recent discovery of *qtz*‐Zn(**Meim**)_2_ by Thorne et al.,^[^ ^115^ ^]^ representing the fourth polymorph (not including the amorphous phase) in the ZIF‐8 family. Calculation of relative stabilities of putative Zn(**Meim**)_2_ and Zn(**Etim**)_2_ framework structures based on CSD data indicated that *dia*, *qtz*, *kat* and SOD topologies should all be accessible for the Zn(**Meim**)_2_ system (Figure 12b). The novel *qtz*‐Zn(**Meim**)_2_ material was obtained by recrystallization of mechanochemically prepared amorphous material at temperatures close to 300 °C, whereas wet‐milling recrystallization yielded *dia* and/or *kat* phases.

Overall, the ability of DFT‐SEDC calculations to accurately appraise the stability of ZIF polymorphs will undoubtedly afford systematic insight into the relationship between choice of metal node and linker structure and the thermodynamic stability of framework materials. This method is, however, inherently limited to framework topologies already reported in structural databases such as the CSD. Thus, new topologies, such as that of *kat*‐Zn(**Meim**)_2_,^[^ ^104^ ^]^ could not have been discovered by the database‐driven approach. This limitation is overcome by the first‐principles (ab initio) Crystal Structure Prediction (CSP) of MOFs, recently introduced by Darby et al.^[^ ^116^ ^]^ CSP^[^ ^117^ ^]^ is a well‐established methodology for investigating organic molecular solids, including hydrogen‐bonded frameworks,^[^ ^118^ ^]^ but until recently was not readily applicable to materials comprising extended covalent bonding. The breakthrough came by combining the ab initio random structure search (AIRSS) algorithm, capable of producing random arrangements of metal node and organic linker fragments in unit cells of varying size, with the Wyckoff alignment of molecules (WAM) approach, which significantly lowers the number of necessary trial calculations by analyzing the compatibility between crystallographic symmetry and point‐group symmetry of putative MOF nodes/linkers assemblies. This powerful computational methodology for MOF discovery was validated on well‐known MOF systems, assisted by mechanochemical synthesis. The combination of ab initio CSP and mechanochemical screening for MOF systems promises to be a valuable tool for the discovery of new MOF structures.^[^ ^116^ ^]^

### 3.7 Understanding the Influence of Linker Substitution on MOF Thermodynamics

**Figure 13:**
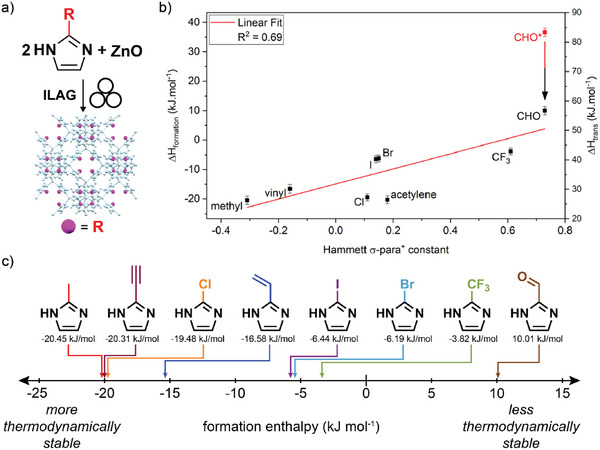
Synthetic details and thermodynamic parameters for SOD‐topology ZIFs with varying 2‐substitutions.^[^ ^119^ ^]^ a) Scheme of the mechanosynthesis of model SOD‐topology ZIFs from ZnO and 2‐substituted imidazoles. b) Plot showing the linear relationship between the enthalpy of formation for a ZIF and the Hammet σ_p_^+^ constant^[^ ^120^ ^]^ of its 2‐substituent. c) Formation enthalpies for each ZIFs displayed with the imidazole used in its synthesis. Panel b) reproduced with permission.^[^ ^119^ ^]^ Copyright 2020, American Chemical Society.

The stability of framework materials is generally expected to increase (more exothermic formation) with increased framework density, i.e. the number of framework *nodes* per unit volume (not to be confused with the actual density of the material). A comparison of the measured thermodynamic stability of polymorphs of Zn(**Etim**)_2_, Zn(**Meim**)_2_ and Zn(**CF_3_Im**)_2_, however, revealed a significant impact of the *linker* on stability, by virtue of the substituent in its 2‐position.^[^ ^114^ ^]^ Varying the substituent from ‐C_2_H_5_ to ‐CH_3_ to ‐CF_3_ led to a consistent drop in thermodynamic stability, independently of framework density (Figure 12c). This relationship between linker and MOF thermodynamic stability was subsequently explored by Novendra et al.^[^ ^119^ ^]^ who used mechanochemical reactions of ZnO with appropriate solid imidazoles to generate eight isostructural ZIFs of SOD‐topology, systematically differing in the choice of the 2‐substituent on the linker (**Figure** **13**a). Dissolution calorimetry experiments on the resulting ZIFs, after washing and evacuation, confirmed that thermodynamic stability was higher for the materials of greater framework densities, which is consistent with observations typically made for other types of microporous materials, such as zeolites. Furthermore, the enthalpy of formation of the ZIFs from ZnO and the corresponding linker exhibited a linear correlation with the Hammett parameters of the 2‐substituent, especially with σ_p_^+^,^[^ ^120^ ^]^ which also considers the ability of the substituent to delocalize the positive charge created by coordination of the linker to the Zn^2+^ cations (Figure 13b).^[^ ^119^ ^]^ By establishing a linear correlation between the enthalpy of formation and a parameter related to chemical composition, this study provided the first empirical guidelines to controlling the thermodynamic stability of MOFs. The observed variation of up to 30 kJ/mol in the thermodynamic stability of the MOFs, corroborated by periodic DFT calculations (Figure 13c), showed that peripheral chemical moieties on the ZIF backbone not only affect the chemical functionality of the framework, but also the thermodynamic stability of the material in a predictable way.

### 3.8 Boron Imidazolate Frameworks (BIFs)

**Figure 14:**
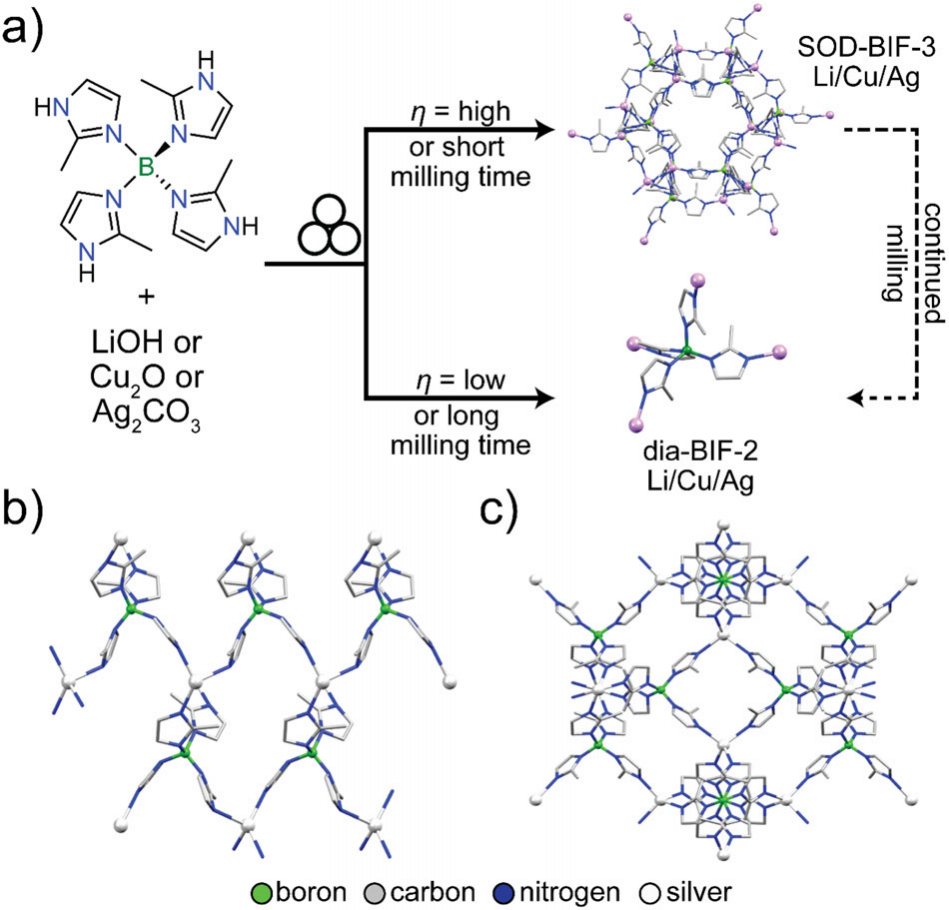
Mechanochemical synthesis of BIF materials.^[^ ^122^ ^]^ a) Mechanochemical approaches for the synthesis of Li ^I^‐, Cu^I^‐ and Ag^I^‐based BIF materials involving **Meim** ^−^ linkers. The dashed line indicates the conversion of SOD to dia framework upon longer milling. b,c) Crystal structures of the mechanochemically prepared polymorphs of the novel AgB(**Meim**)_2_ BIF material with (b) close‐packed *dia*‐topology and (c) open‐framework SOD‐topology. Reproduced under the terms of the CC‐BY 3.0 license.^[^ ^122^ ^]^

Mechanochemistry has proven highly successful at rendering accessible materials that had previously been obtained only in small amounts or through arduous synthetic procedures. An excellent illustration are BIFs, bimetallic MOFs consisting of equimolar amounts of mono‐ and trivalent tetrahedral nodes. The materials were introduced in 2009 by the Bu group,^[^ ^121^ ^]^ who combined a trivalent boron node with Cu^I^ or Li nodes to make structural analogues of ZIFs. The use of lithium ions is especially attractive to make materials of low formula weight and, consequently, high gravimetric capacities for gas storage. These BIF materials were, however, accessed by solvothermal methods, often in high‐boiling‐point solvents and by using aggressive reagents such as *n*‐butyllithium. In contrast, Lennox et al. showed that the synthetic methodology used to access ZIFs, i.e., mechanochemical milling starting from simple inorganic precursors such as oxides, can be used for room‐temperature, rapid synthesis of BIF materials.^[^ ^122^ ^]^ Mechanochemical procedures successfully yielded Cu^I^‐ and Li‐based BIFs, and the use of Ag^I^‐based precursors led to the previously not reported Ag‐BIFs, structurally characterized from PXRD data, in which tetrahedral boron nodes are combined with Ag^I^ ones (**Figure** **14**).

In an analogous manner to ZIFs, different polymorphs of BIFs can be obtained by varying the reaction conditions. A step‐by‐step time‐resolved investigation of the reactions revealed that the higher‐porosity SOD‐topology polymorph of the Cu^I^‐based BIF formed initially but rearranged into the low‐porosity *dia*‐polymorph upon extended milling. A similar behavior was also observed for Ag^I^‐based systems.^[^ ^122^ ^]^ A theoretical investigation of the effect of metal node on BIF thermodynamic stability, made possible by the availability of the crystal structures for *dia*‐ and SOD‐topology polymorphs of BIFs containing Li^I^, Cu^I^ and Ag^I^ nodes, revealed that using metal ions of higher atomic number increased the stabilization of the less dense SOD‐topology relatively to the denser *dia* topology (Figure 12b).

### 3.9 Edible MOFs

A class of microporous MOF materials that emerged recently consists of alkali metal nodes, such as Na^+^, K^+^, Cs^+^ and linkers based on β‐cyclodextrin (β‐CD) macrocycles.^[^ ^123^ ^]^ This class of materials known as cyclodextrin MOFs (CD‐MOFs), and popularly termed “edible MOFs”, have attracted attention due to the low toxicity and biocompatibility of both nodes and linkers. In 2021, Kang et al. reported a mechanochemical synthesis of edible MOFs by using a laboratory blender to perform the reaction of KOH and β‐CD on a 6‐gram scale over 60 min.^[^ ^124^ ^]^ Subsequently, Fujita et al. established a more general approach to edible MOFs with ball milling protocols on β‐CD and a range of metal salts, including KCl, potassium acetate, KOH, K_2_CO_3_ and KHCO_3_, in the presence of EtOH as a liquid additive.^[^ ^125^ ^]^

## 4 Mechanistic Studies

### 4.1 Step‐by‐Step, Ex Situ Monitoring

**Figure 15:**
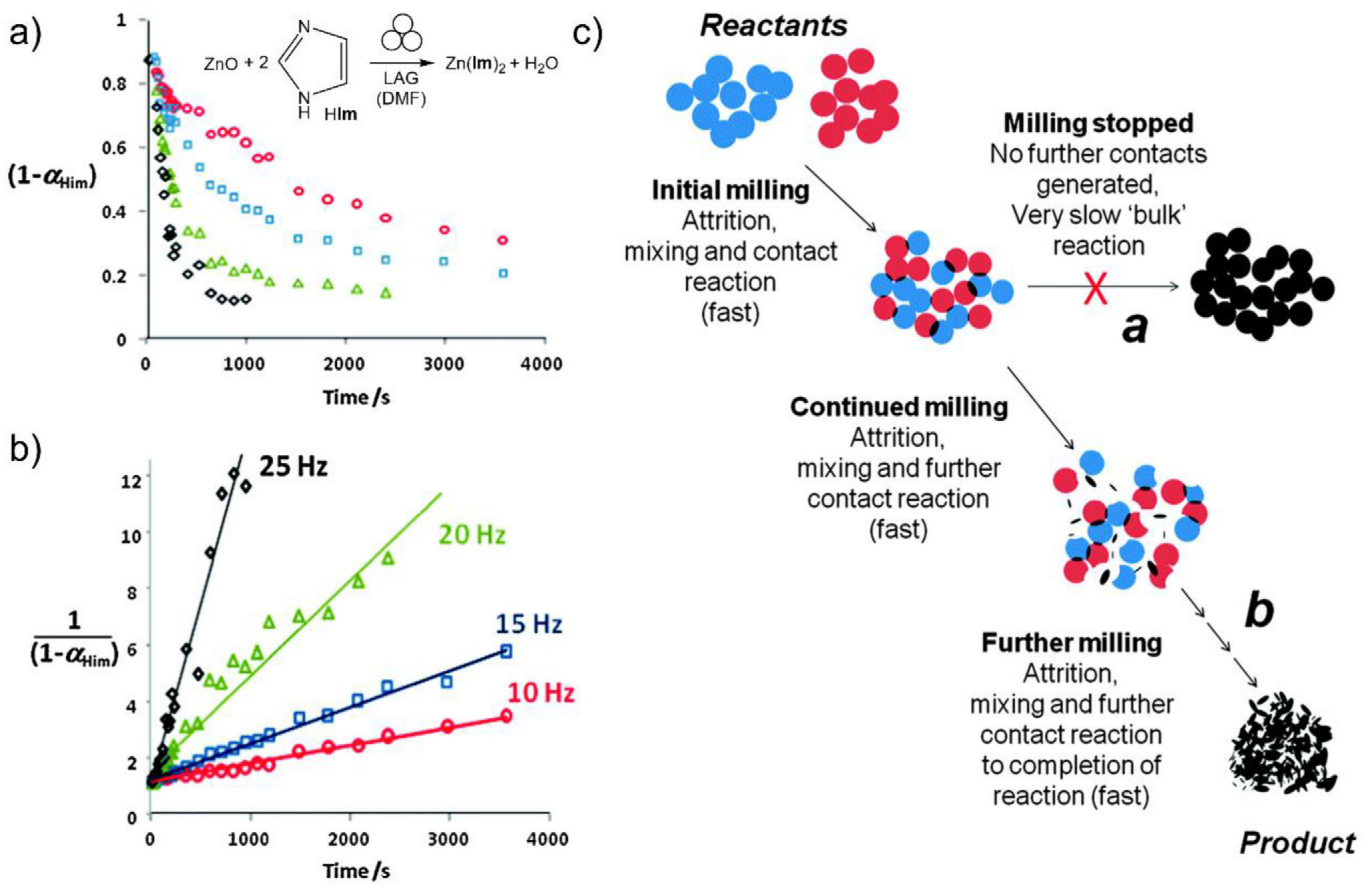
Raman spectroscopy investigation of the LAG reaction of ZnO and H**Im** to yield Zn(**Im**)_2_ framework.^[^ ^126^ ^]^ a) Kinetic data shown as conversion of H**Im** (given as 1‐α) at different milling frequencies, with the reaction equation shown. b) Pseudo 2^nd^‐order plot of the kinetic data. c) Scheme of the proposed pseudo‐fluid model of reactivity. Reproduced with permission.^[^ ^126^ ^]^ Copyright 2014, The Royal Society of Chemistry.

Early studies on the mechanism of mechanochemical MOF formation were based on stepwise, ex situ analysis, by periodically interrupting the milling process and analyzing an extracted aliquot of the milled sample by spectroscopy, diffraction, or other methods. The James group used Raman spectroscopy to follow the course of ball‐milling reactions of ZnO and H**Im** to produce Zn(**Im**)_2_·*n*H_2_O (also known as ZIF‐6). The reactions followed an apparent second‐order kinetics, with the rate constant dependent only on frequency of the milling process (**Figure** **15**a,b).^[^ ^126^ ^]^ The increase in temperature during milling, sometimes by up to 30 °C, and the fact that the reaction releases water, which should facilitate diffusion of reactants, were secondary to the milling frequency in controlling the reaction kinetics. This was explained through a pseudo‐fluid model of mechanochemical reactivity, wherein the reaction rate depends on the many reactive contacts created between particles rather than the actual rate of the reaction at the particle interface (Figure 15c).^[^ ^126^ ^]^

Another interesting example of step‐by‐step reaction monitoring was presented by the Emmerling group who used rapid ex situ sampling to follow the course of the mechanochemical reaction of Bi(NO_3_)_3·_5H_2_O with H**Im** and H_2_ **ta**.^[^ ^127^ ^]^ With sampling times as low as 10 s, this tour de force of real‐time monitoring revealed that the formation of the anionic MOF product, [H_2_ **Im**][Bi(**ta**)_2_], progressed first through the formation of basic bismuth nitrate, which indicated reorganization of the bismuth coordination sphere and protonation of H**Im** before reaction with terephthalic acid.

### 4.2 Step‐by‐Step, In Situ Monitoring

**Figure 16:**
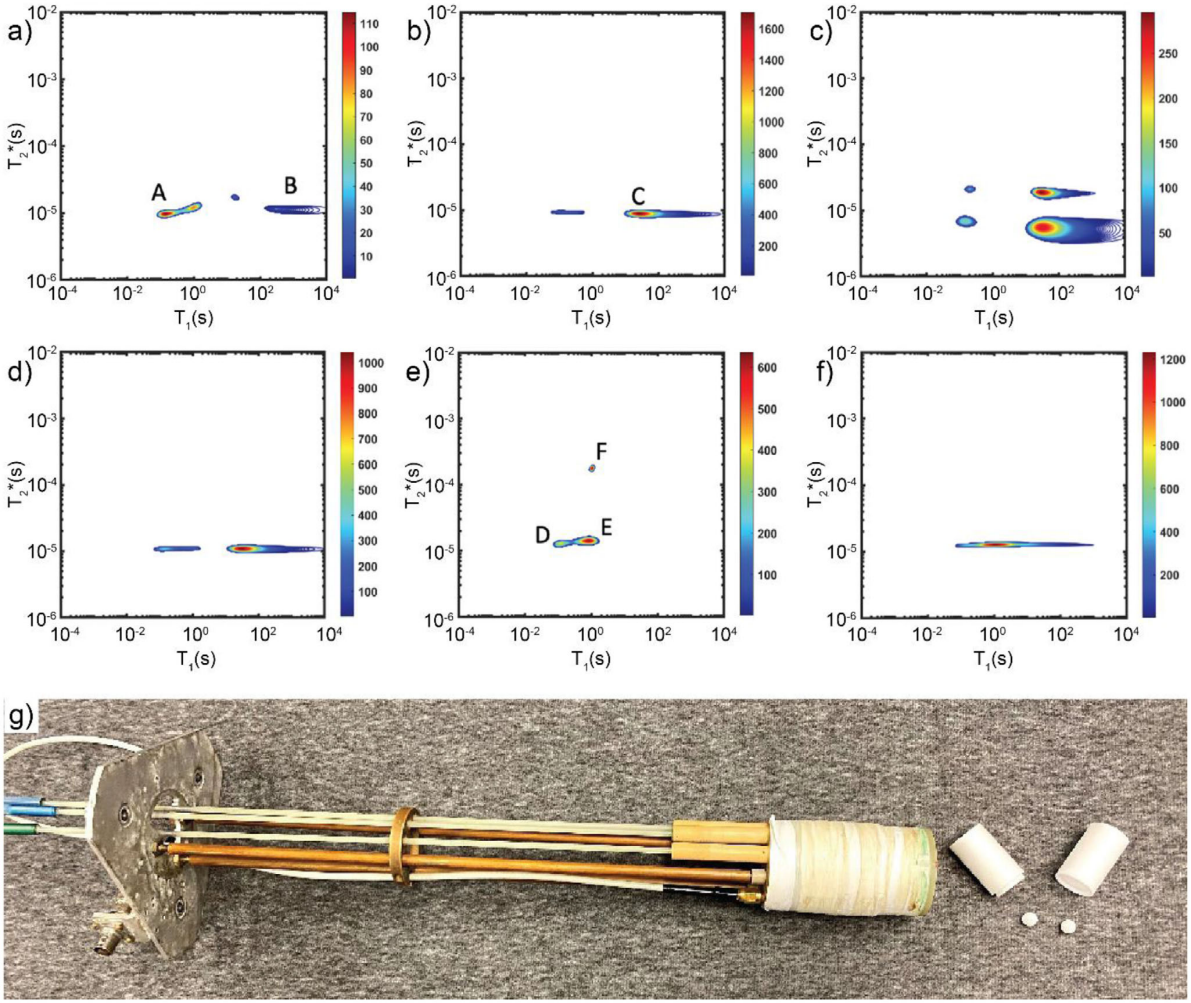
Monitoring the formation of ZIF‐8 using ^1^H‐NMR.^[^ ^128^ ^]^ a)‐f) Relaxation time correlation maps for reactants, reactant mixture, as well as the crude and purified product form ball‐milling ZIF‐8 synthesis. g) The probe used for NMR measurements, along with the milling jar and balls used for MOF synthesis. Reproduced with permission.^[^ ^128^ ^]^ Copyright 2024, The Royal Society of Chemistry.

An attractive methodology has been outlined by the Blight and Balcom groups to investigate the progress of mechanochemical reactions by nuclear magnetic resonance (NMR), specifically by looking at T1‐T2^*^ relaxation correlation maps and by focusing on spin relaxation properties of protons in liquid and solid samples (**Figure** **16**).^[^ ^128^ ^]^ Performed with a benchtop NMR instrument, the methodology enabled reaction monitoring by analyzing the entire sample in the reaction container, without having to extract the sample and prepare it for measurement. While the measurements were not performed during ball‐mill operation, they are nevertheless considered to be in situ, as the whole reaction mixture remained in the reaction vessel throughout the experiment.

### 4.3 Real‐Time, In Situ Monitoring

The development of methodologies for real‐time monitoring^[^ ^15^ ^]^ of mechanochemical reactions has enabled unprecedented insight into the course of mechanochemical MOF synthesis, involving the detection of reaction intermediates and advances in the understanding of underlying thermodynamics. Real‐time monitoring of MOF mechanosynthesis can be accomplished indirectly, through monitoring of the pressure or temperature of the reaction mixture as the process advances, or directly, through real‐time detection of structural and chemical changes taking place in the sample by means of X‐ray diffraction, spectroscopy, or a combination of both. Combining synchrotron X‐ray diffraction and Raman spectroscopy^[^ ^129^ ^]^ provides the advantage of furnishing molecular‐level information about the crystalline, amorphous, liquid, or even gaseous components of the reaction mixture via Raman scattering, concomitantly with providing information about crystalline, long‐range order via PXRD, as illustrated by real‐time monitoring of the formation of ZIF‐8 and [H_2_ **Im**][Bi(**ta**)_2_].^[^ ^130^ ^]^

An example of an indirect approach to monitoring the course of MOF mechanosynthesis was reported by Brekalo et al., who followed pressure changes during the planetary milling of basic zinc carbonate with solid H**Im** or H**Meim**, to form *zni*‐Zn(**Im**)_2_ or ZIF‐8, respectively.^[^ ^53^ ^]^ The reaction produces carbon dioxide (CO_2_) gas as a byproduct, providing a suitable handle for manometric monitoring of the process. Whereas the synthesis of *zni*‐Zn(**Im**)_2_ by neat milling or LAG was characterized by a monotonic increase in the pressure within the reaction vessel, the formation of ZIF‐8 exhibited a pressure maximum. PXRD analysis revealed the occurence of a parasitic reaction of the initially formed ZIF‐8 with the CO_2_ and water byproducts to form a metal–organic carbonate phase, Zn_2_(**Meim**)_2_(CO_3_). The same carbonate phase was obtained upon exposure of ZIF‐8 to moist CO_2_^[^ ^131^ ^]^ in a highly thermodynamically‐driven process.^[^ ^132^ ^]^ This side reaction could be alleviated by using an excess of H**Meim**, enabling multi‐gram mechanosynthesis of ZIF‐8 without liquid additives.

**Figure 17:**
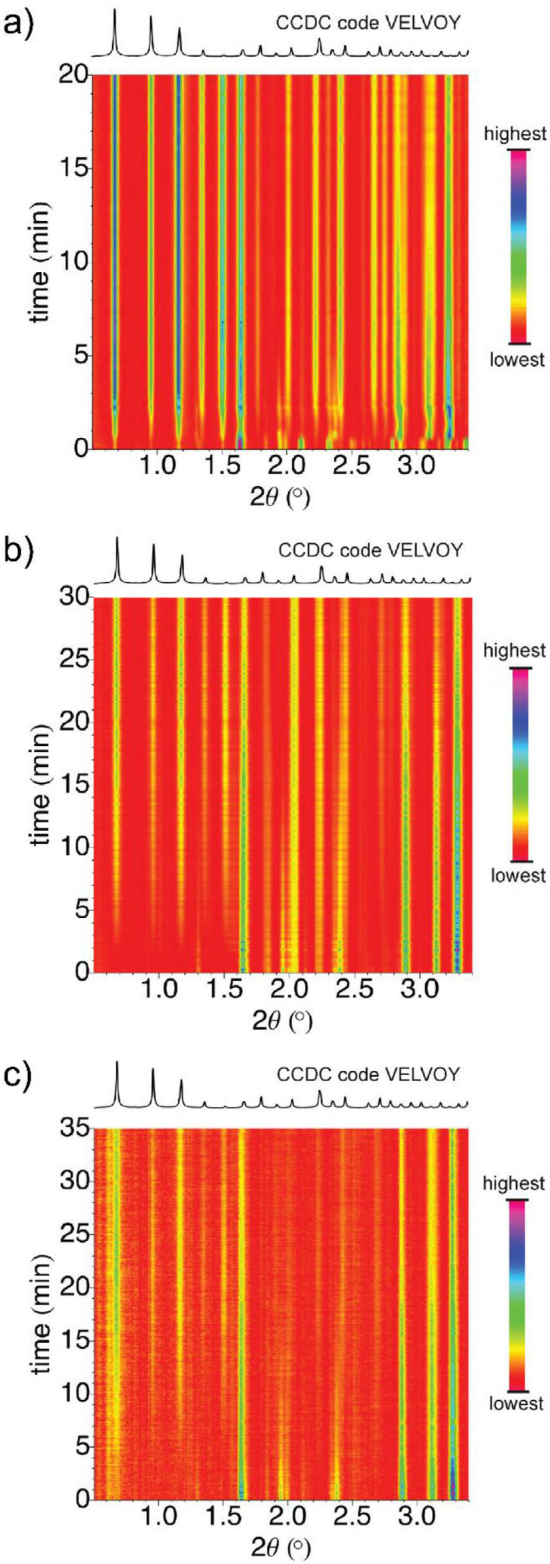
Time‐resolved diffractograms for in situ, real‐time monitoring^[^ ^59^ ^]^ of the mechanochemical synthesis of ZIF‐8 from ZnO and H**Meim**, performed in milling jars made of: a) PMMA plastic, ILAG reaction; b) aluminum, neat reaction in the presence of NH_4_NO_3_, and c) steel, neat reaction in the presence of NH_4_NO_3_, demonstrating the ability to observe the course of the reaction through different materials. Reproduced with permission.^[^ ^59^ ^]^ Copyright 2012, the authors.

A methodology for the real‐time monitoring of the progress of ball‐milling reactions was reported in 2013,^[^ ^59^ ^]^ which involved the use of synchrotron X‐rays to follow the course of ZIF formation from ZnO. The technique, which was readily applicable to reactions conducted in steel, aluminum and plastic (polymethylmetacrylate, PMMA) vessels provided unprecedented insight into the kinetics and intermediates during mechanochemical MOF synthesis (**Figure** **17**).

**Figure 18:**
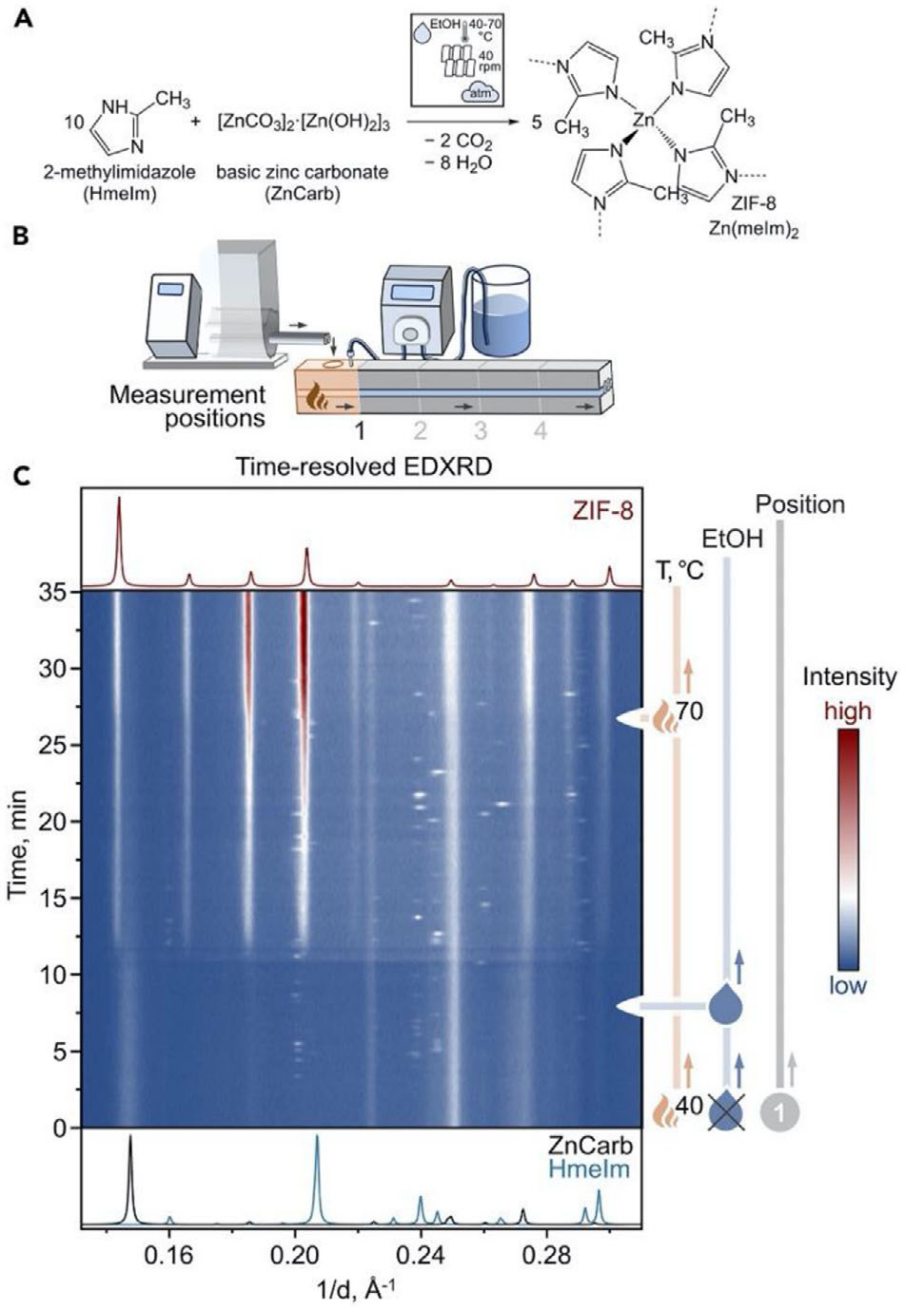
Real‐time EDXRD monitoring of ZIF‐8 synthesis by liquid‐assisted TSE of basic zinc carbonate and H**Meim**.^[^ ^134^ ^]^ a) Scheme of the reaction. b) Scheme of the experimental design. c) Time‐ and space‐resolved X‐ray diffractogram. Reproduced with permission.^[^ ^134^ ^]^ Copyright 2024, Elsevier.

The use of synchrotron PXRD to monitor the mechanochemical formation and transformations of MOFs, as well as other types of materials such as cocrystals, organic molecules, or inorganic systems such as CsCl and KI, has been adapted by Lampronti et al. to accommodate work with small amounts of sample (ca. 10–100 mg).^[^ ^133^ ^]^ Very recently, Gugin et al. have adapted and expanded the technology for real‐time synchrotron X‐ray diffraction monitoring of mechanochemical reactions by using energy‐dispersive X‐ray ray diffraction (EDXRD) to follow the progress of continuous manufacturing transformations in a twin‐screw extruder (**Figure** **18**).^[^ ^134^ ^]^

The ability to observe reaction progress in real time has provided deeper insight into the mechanistic aspects and kinetic profiles of the mechanochemical reactions. Whereas ZIF‐8 synthesis by LAG or ILAG of ZnO with H**Meim** proceeded without detectable crystalline intermediates, the analogous reaction with H**Etim** involved the intermediate formation of increasingly dense Zn(**Etim**)_2_ frameworks with RHO‐, ANA‐, and *qtz*‐topology (Figure 11d).^[^ ^59^ ^]^ Real‐time monitoring also enabled the detection of changes induced by varying the amount of liquid additive: smaller amounts of DMF (lower *η*‐values) generally delayed the initial formation of the RHO‐framework and shortened the lifetime of the ANA‐topology intermediate, presumably by preventing the stabilization of open frameworks by inclusion of DMF. To enable quantitative analysis of the reaction kinetics, the reaction of ZnO and H**Im** was subsequently conducted by LAG or ILAG in the presence of crystalline silicon (Si) powder as an X‐ray diffraction standard.^[^ ^135^ ^]^ Quantitative Rietveld analysis of in situ data exposed a behavior resembling first‐order reaction kinetics. Whereas this observation is different from the second‐order reaction kinetics obtained by the James group via ex situ Raman spectroscopy data collected in a stepwise fashion, it remains consistent with the proposed pseudofluid model of mechanochemical reactivity.

While real‐time synchrotron PXRD observation of the mechanochemical formation of ZIF‐8 upon LAG or ILAG of ZnO and H**Meim** in the presence of organic liquid additives did not reveal the appearance of other Zn(**Meim**)_2_ polymorphs, milling in the presence of water‐containing liquid phases revealed a richer topological landscape. In particular, the milling reaction in the presence of dilute aqueous acetic acid proceeded through rapid initial formation of ZIF‐8, followed by amorphization and subsequent crystallization to form a previously not known polymorph of Zn(**Meim**)_2_ exhibiting a novel katsenite (*kat*) topology.^[^ ^104^ ^]^ The *kat*‐Zn(**Meim**)_2_ phase subsequently converted to the known *dia*‐Zn(**Meim**)_2_. The synthesis was performed 17 times, with the *kat*‐Zn(**Meim**)_2_ phase being observed in 9 cases. The near‐50% chance of observing the *kat* phase, even when conducting nominally identical experiments, was interpreted as being indicative of stochastic nucleation effects, highlighting that conventional ball‐milling conditions are not necessarily structure‐disrupting, but can support the nucleation and formation of new crystalline phases.^[^ ^104^ ^]^ The multi‐step sequences of topologically distinct phases observed by in situ and ex situ monitoring of the mechanosynthesis of Zn(**Meim**)_2_ and Zn(**Etim**)_2_ were subsequently found to reflect the relative thermodynamic stabilities, expressed as formation enthalpies, of the observed phases. This observation indicates that the mechanochemical synthesis of Zn(**Meim**)_2_ and Zn(**MEtim**)_2_ follows Ostwald's rule of stages, i.e. the initial formation of metastable phase which transforms into the thermodynamically most stable product through a series of increasingly stable intermediates.

**Figure 19:**
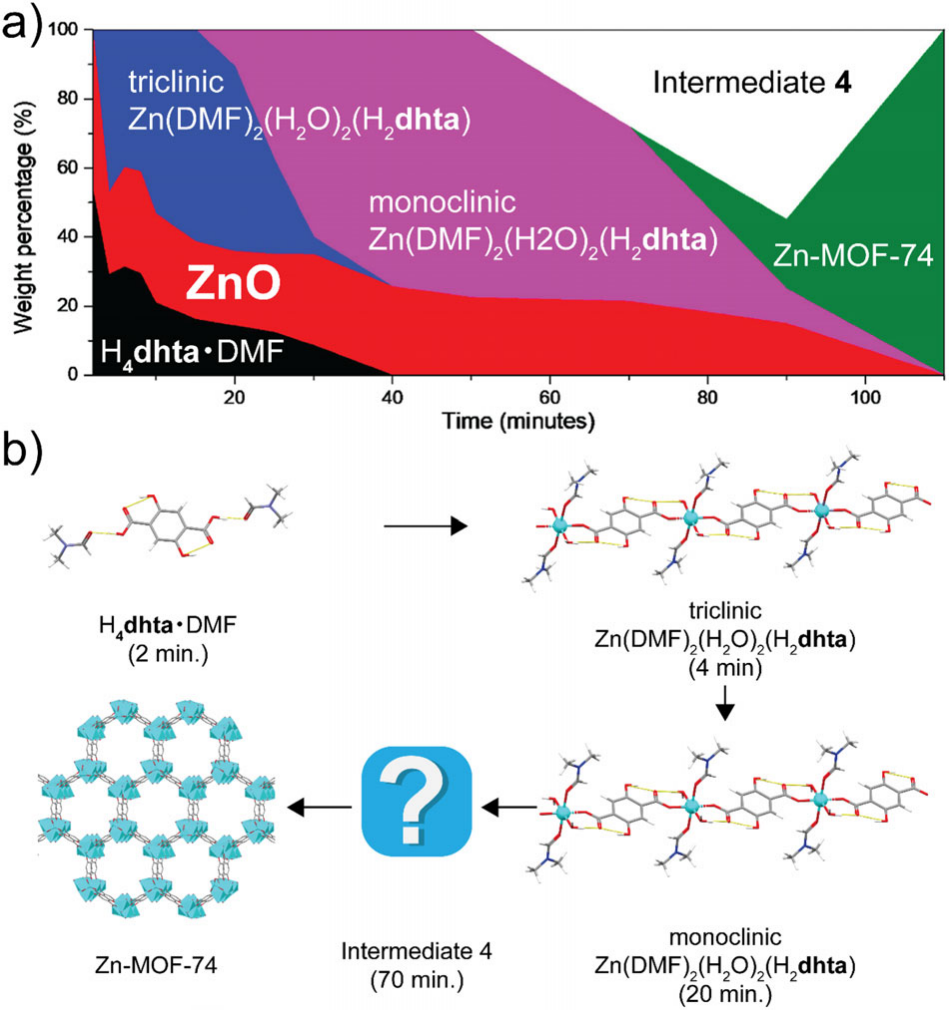
The stepwise mechanism of the mechanochemical synthesis of Zn‐MOF‐74 by LAG from ZnO and H_4_ **dhta** in the presence of DMF.^[^ ^137^ ^]^ a) Composition of reaction mixture with time, based on real‐time in situ PXRD analysis. b) Schematic representation of the reaction. Adapted under the terms of the CC‐BY 4.0 license.^[^ ^137^ ^]^

The time‐resolved in situ analysis of mechanochemical MOF formation has also been expanded to other systems, including the synthesis of Zn‐MOF‐74 by LAG of ZnO and 2,5‐dihydroxyterephthalic acid (H_4_ **dhta**).^[^ ^136^, ^137^ ^]^ Generally, the observation of crystalline intermediates was found to be dependent on the choice of LAG additive. Milling in the presence of water or aqueous DMF first generated a coordination polymer of composition Zn(H_2_O)_4_(H_2_ **dhta**), indicating the selective deprotonation of the most acidic sites of H_4_ **dhta** (carboxylic acid functions). Further milling led to deprotonation of the hydroxy moieties of H_4_ **dhta** by unreacted ZnO, resulting in the formation of the Zn‐MOF‐74 framework of composition Zn_2_(**dhta**).^[^ ^136^ ^]^ A more complex mechanism, involving four intermediate crystalline phases, has been reported by Beamish‐Cook et al. based on real‐time in situ monitoring of the mechanochemical synthesis of Zn‐MOF‐74 from ZnO and H_4_ **dhta** in the presence of DMF as the sole liquid additive (**Figure** **19**a,b).^[^ ^137^ ^]^ In this case, the reaction proceeded with an almost immediate appearance of the crystalline solvate of H_4_ **dhta** and DMF, followed by the formation of a triclinic polymorph of the 1D coordination polymer of composition Zn(H_2_O)_2_(DMF)_2_(H_2_ **dhta**), that can be seen as analogous to the 1D polymer Zn(H_2_O)_4_(H_2_ **dhta**) intermediate observed when using water as the liquid additive. Further milling led to another monoclinic polymorph of Zn(H_2_O)_2_(DMF)_2_(H_2_ **dhta**), which subsequently transformed into a not yet identified crystalline phase, which was ultimately replaced by the target product Zn‐MOF‐74. It is important to note that the effect of liquid additive on MOF synthesis by LAG has not been frequently studied by in situ methods. A comparison of the reports of Julien et al. and Beamish‐Cook et al. highlights important mechanistic differences, including the appearance of qualitatively different types of intermediates, that can occur when investigating the optimization of mechanochemical MOF synthesis by changes to the liquid additive.

### 4.4 Monitoring Changes in Particle Size and Material Structure

**Figure 20:**
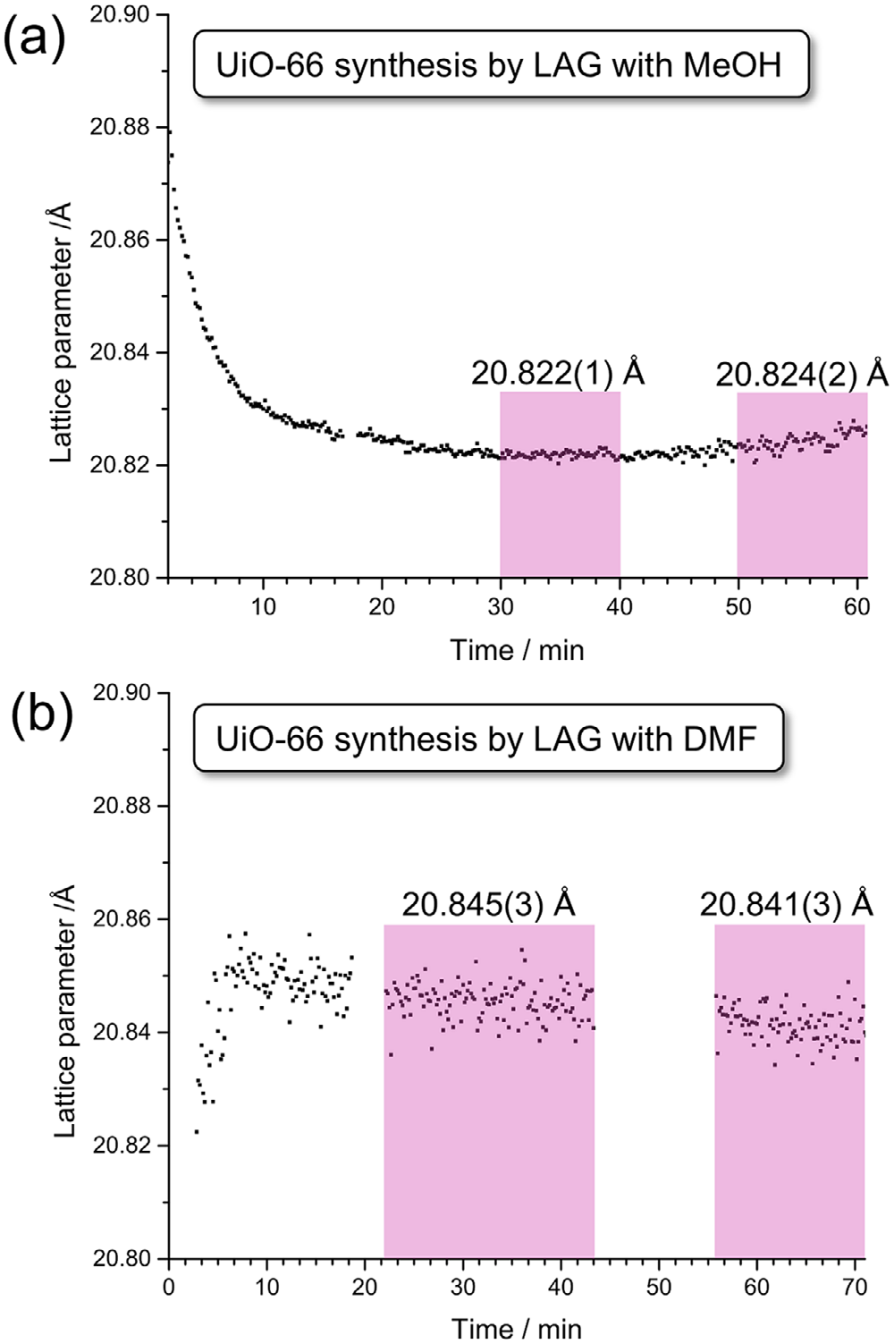
Different trends in the evolution of the product crystal structure, characterized by changes in the crystallographic unit‐cell dimension *a*, observed during LAG synthesis of UiO‐66 using:^[^ ^138^ ^]^ a) MeOH and b) DMF as the liquid additive. The most significant differences between two experiments are seen within the first 10 minute of milling. Reproduced under the terms of the CC‐BY 4.0 license.^[^ ^138^ ^]^

In addition to allowing the detection of reaction intermediates and the discovery of new phases, real‐time X‐ray diffraction monitoring can be used to track particle size and structure evolution in the early stages of mechanochemical reactions. This was explored by Germann et al. with the mechanochemical formation of UiO‐66 from the methacrylate Zr_6_‐cluster.^[^ ^138^ ^]^ Whereas the reaction yielded the target MOF directly, without any crystalline intermediates, application of the Scherrer analysis to in situ measured Bragg maxima revealed trends in the evolution of particle size and structure during the first 10 min of the reaction. In particular, the unit‐cell parameter *a* of the cubic UiO‐66 product varied significantly in the early stages of the reaction, with a dependence on the choice of liquid additive. In the presence of MeOH, the *a* lattice parameter decreased monotonically throughout the reaction, while the use of DMF resulted in a sequence where *a* first rapidly increased, then decreased (**Figure** **20**). This type of real‐time X‐ray diffraction‐based structural analysis, that focuses on changes in lattice parameters and crystallite sizes with time, represents an exciting and, to date, mostly overlooked area of study for mechanochemical synthesis.^[^ ^138^ ^]^

### 4.5 Real‐Time NMR Monitoring

**Figure 21:**
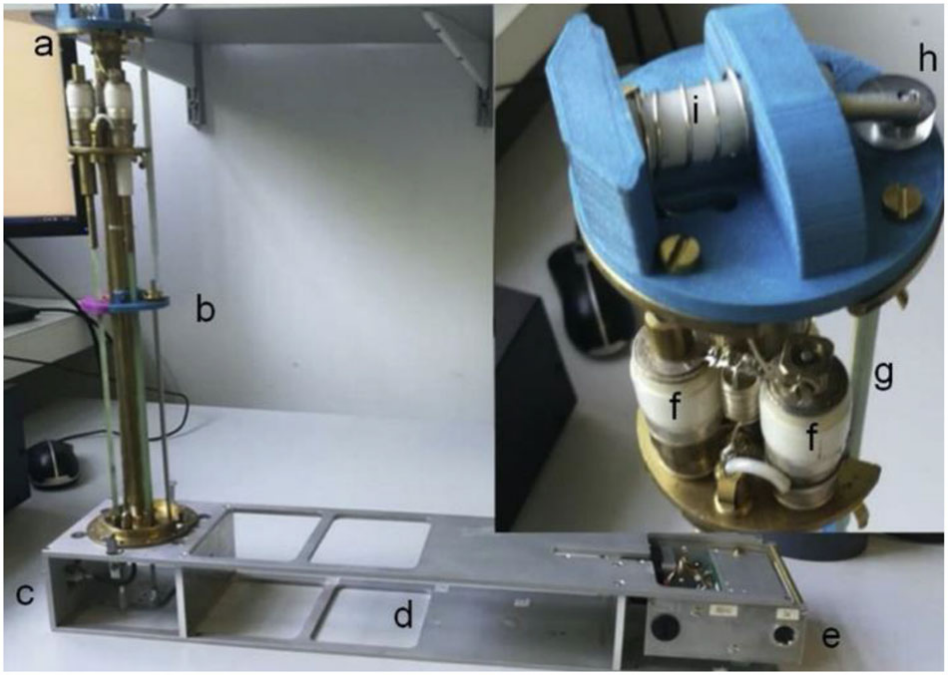
The experimental setup used for real‐time monitoring of a mechanochemical reaction by solid‐state NMR spectroscopy, as reported by the Van Wüllen group.^[^ ^139^ ^]^ Reproduced with permission.^[^ ^139^ ^]^ Copyright 2020, Elsevier.

Whereas most applications of NMR spectroscopy in the context of monitoring mechanochemical reactions have focused on observing the transformations of mechanically‐activated materials or reaction mixtures within a solid‐state NMR rotor, a methodology for real‐time spectroscopic analysis of a material during mechanical treatment has been reported by the Van Wüllen group.^[^ ^139^ ^]^ The technique featured a miniaturized version of a vibratory ball mill that was incorporated in the measuring coil of a home‐built solid‐state NMR probe (**Figure** **21**) and was applied to the mechanochemical synthesis of a metal–organic product from ZnO and phenylphosphonic acid. This methodology allows static solid‐state NMR measurements during the mechanochemical reaction and opens new opportunities for mechanistic studies of MOF formation.

## 5 Advanced Approaches to MOF Design and Discovery

### 5.1 Mechanochemistry and NMR‐Enabled Crystallography

**Figure 22:**
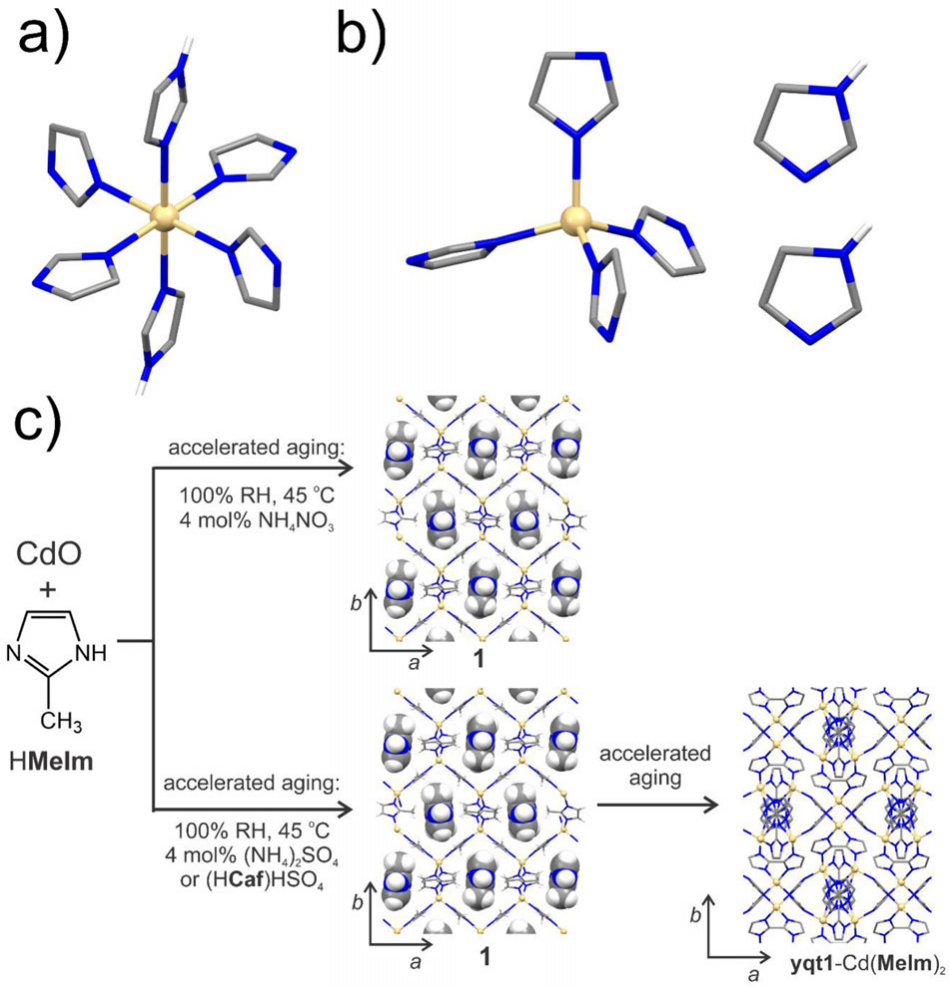
Different potential models for the structure of the framework node for the product of reaction of CdO and H**Meim** in the respective 1:3 stoichiometric ratio.^[^ ^140^ ^]^ a) Octahedrally coordinated node. b) Tetrahedrally coordinated node with adjacent non‐coordinated imidazoles. Substituents on H**Meim** are not shown for clarity. c) Schematic overview of the reaction facilitated by different protic salts. Reproduced with permission.^[^ ^140^ ^]^ Copyright 2020, American Chemical Society.

Although mechanochemical synthesis is known to generate new materials, even in previously well‐studied systems, the characterization of such materials requires the use of sophisticated tools suitable for analysis of micro‐ or even nano‐crystalline samples. In this context, solid‐state nuclear magnetic resonance (NMR) spectroscopy offers structural information that is not accessible through PXRD alone. An example of such NMR‐enhanced crystallography approach is demonstrated by O'Keefe et al.,^[^ ^140^ ^]^ who established that mechanically activated aging of a mixture of cadmium oxide (CdO) and H**Meim** in the presence of a small amount of protic salt catalyst initially produced a novel crystalline phase which subsequently transformed into the known *yqt1*‐topology Cd(**Meim**)_2_ (**Figure** **22**). The new phase was found to have the composition Cd(**Meim**)_2_·H**Meim**, and could be obtained quantitatively by conducting the reaction using CdO and H**Meim** in a 1:3 stoichiometric ratio. Two potential structural models for Cd(**Meim**)_2_·H**Meim** were envisaged, one based on tetrahedrally coordinated Cd^2+^ nodes with H**Meim** molecules included as guests in framework cavities, and another with the H**Meim** molecule acting as an additional neutral ligand on the cadmium center (Figure 22). The use of a range of NMR‐based approaches, including magic‐angle spinning (MAS) ^111^Cd chemical shift anisotropy measurement, ^1^H‐^111^Cd correlations, as well as ^111^Cd‐^14^N spin‐spin coupling provided strong indication that the product was indeed based on tetrahedral nodes, which facilitated analysis of the PXRD structural data.^[^ ^140^ ^]^

### 5.2 Mechanochemistry and Electron Diffraction

Since the pioneering work of Jones, Ramdas and Thomas,^[^ ^141^ ^]^ electron diffraction (ED) on microcrystals has been recognized as an attractive, powerful tool for structural analysis of materials obtainable only in the form of very small crystallites. Over the past decade, advanced instrumentation has made ED protocols more accessible and widely applicable, providing an exciting opportunity to characterize the products of mechanochemical reactions that often appear in the form of crystals of sub‐micrometer dimensions. An application of ED to MOFs was reported very recently by the Gemmi group with the mechanosynthesis of an open Cu^II^‐based MOF by milling protocatechuic acid (H_3_ **pa**) with copper(II) acetate.^[^ ^142^ ^]^ Whereas the LAG reaction was complete within 20 min when using water as the liquid additive, the synthesis from aqueous solution only led to partial conversions, which was explained through reversibility of the reaction in water. The ED experiments on four separate sub‐micrometer crystals generated by LAG revealed a consistent unit cell and enabled the high‐quality resolution of the crystal structure, including the hydrogen atoms of the MOF linker and oxygen atoms of a disordered water molecule included in the framework pore. Because of the high vacuum environment used for the ED experiment, the MOF was partially desolvated, as evidenced by structure analysis and Rietveld analysis of the sharp PXRD data.

### 5.3 Mixed‐Metal or High‐Entropy (HE) MOFs

The high degree of stoichiometric control often observed for mechanochemical synthesis has been harnessed to produce MOFs with multiple types of metal nodes, termed mixed‐metal or high‐entropy framework materials. In an early example,^[^ ^143^ ^]^ a mixture of Ni^2+^, Cd^2+^, Co^2+^, Cu^2+^, and Zn^2+^ salts were ball‐milled with H**Meim** to produce a SOD‐topology material isostructural to ZIF‐8, but with the lattice parameter slightly larger than that of ZIF‐8 or its Co^II^ analog ZIF‐67, but smaller than that of the analogous material containing only Cd^II^ nodes. Diffraction analysis of the residues following thermogravimetric analysis (TGA) showed the presence of oxides of all five used metal ions. In contrast, TGA of an analogous material synthesized solvothermally revealed the incorporation of only Zn^II^ and Co^II^ in the framework nodes.^[^ ^143^ ^]^ The fact that Cu^II^, Ni^II^ and Cd^II^ are not incorporated into the high‐entropy ZIF underscores a competition chemistry between the ions in solution upon formation of the solid material. This further highlights the stoichiometric precision associated with mechanosynthesis, where such competition is quelled in the solid state. The mechanochemically made HE‐ZIFs also showed higher catalytic activity than ZIF‐8 for the conversion of CO_2_ to cyclic carbonates, possibly due to increased Lewis acidity arising from synergistic effects between different metal sites. In a related report, Li and co‐workers have reported the synthesis of a nickel‐doped ZIF‐8 material by ball‐milling of ZnO, Ni^II^ acetate and H**Meim** in the presence of small amounts of EtOH. The reported LAG process gave rise to a green Ni^II^‐containing material BIT‐11, isostructural to ZIF‐8, that readily changed colour to violet upon exposure to MeOH, which was explained by changes in coordination geometry of Ni^II^ centres in the structure.^[^ ^144^ ^]^ A potential application of such metal‐doped ZIF materials is as precursor in the synthesis of metal‐and nitrogen‐containing carbon materials with catalytic applications. For example, Kong and co‐workers^[^ ^145^ ^]^ have described the synthesis of manganese‐ or iron‐containing nitrogen‐doped carbons by pyrolysis of ZIF‐8 samples that were obtained by mechanochemical reaction of ZnO and H**Meim** in the presence of corresponding metal salts and water. Zhang and co‐workers reported that ball‐milling of Zn(OAc)_2_·2H_2_O with H_2_MoO_4_ and ZIF‐8 in the presence of an organic surfactant leads to a molybdenum‐containing ZIF‐8 material which, upon calcination in nitrogen, can serve as a precursor to nitrogen‐dopen carbon materials containing MoC.^[^ ^146^ ^]^

Milling a mixture of metal salts in known stoichiometry with an organic linker appears to be a general approach to synthesize MOFs with precise mixed‐metal composition, facilitating the creation of new materials and enabling the fundamental studies of MOF structure and properties. As an example, mixed Co/Zn analogs of ZIF‐62 produced by this method revealed that a higher cobalt ratio in ZIF‐62 resulted in lower melting temperatures.^[^ ^147^ ^]^ Similarly, mixed‐metal and mixed‐ligand ZIFs containing Zn^II^ and Co^II^ nodes in combination with H**Meim** and trifluoromethylated H**CF_3_Im** linkers have been synthesized by ball‐milling.^[^ ^148^ ^]^ The synthesis was guided by in situ PXRD to steer the production of the SOD‐ or the *qtz*‐topology ZIFs selectively. Following carbonization, the ZIFs produced active catalysts for the fuel cell oxygen reduction reaction (ORR), with no notable difference between catalysts derived from the SOD‐ and *qtz*‐topology frameworks. Recently, an analog to HKUST‐1 containing both Ru^III^ and Cu^II^ nodes was produced by LAG of Cu(OAc)_2_, RuCl_3_, and H_3_ **btc** in the presence of a small amount of MeOH.^[^ ^149^ ^]^ The process exhibited a high degree of stoichiometric control, producing MOF materials that acted as precursors to highly active metal oxide/hydroxide catalysts for the oxygen evolution reaction. Ye et al. described the synthesis of a high‐entropy MOF‐74 material containing five different metal nodes (Zn^II^, Mg^II^, Cu^II^, Co^II^, Ni^II^,) by planetary ball‐milling of ZnO, MgO, Cu(OH)_2_, Co(OAc)_2_·4H_2_O, and Ni(OAc)_2_·4H_2_O with H_4_ **dhta**.^[^ ^150^ ^]^ Remarkably, the resulting mixed‐metal material showed a BET surface area higher than the corresponding monometallic MOFs, as well as higher catalytic activity in the cycloaddition of CO_2_ and propylene oxide to form cyclic carbonates.

### 5.4 Target‐Oriented Synthesis of Mixed‐Metal MOFs

**Figure 23:**
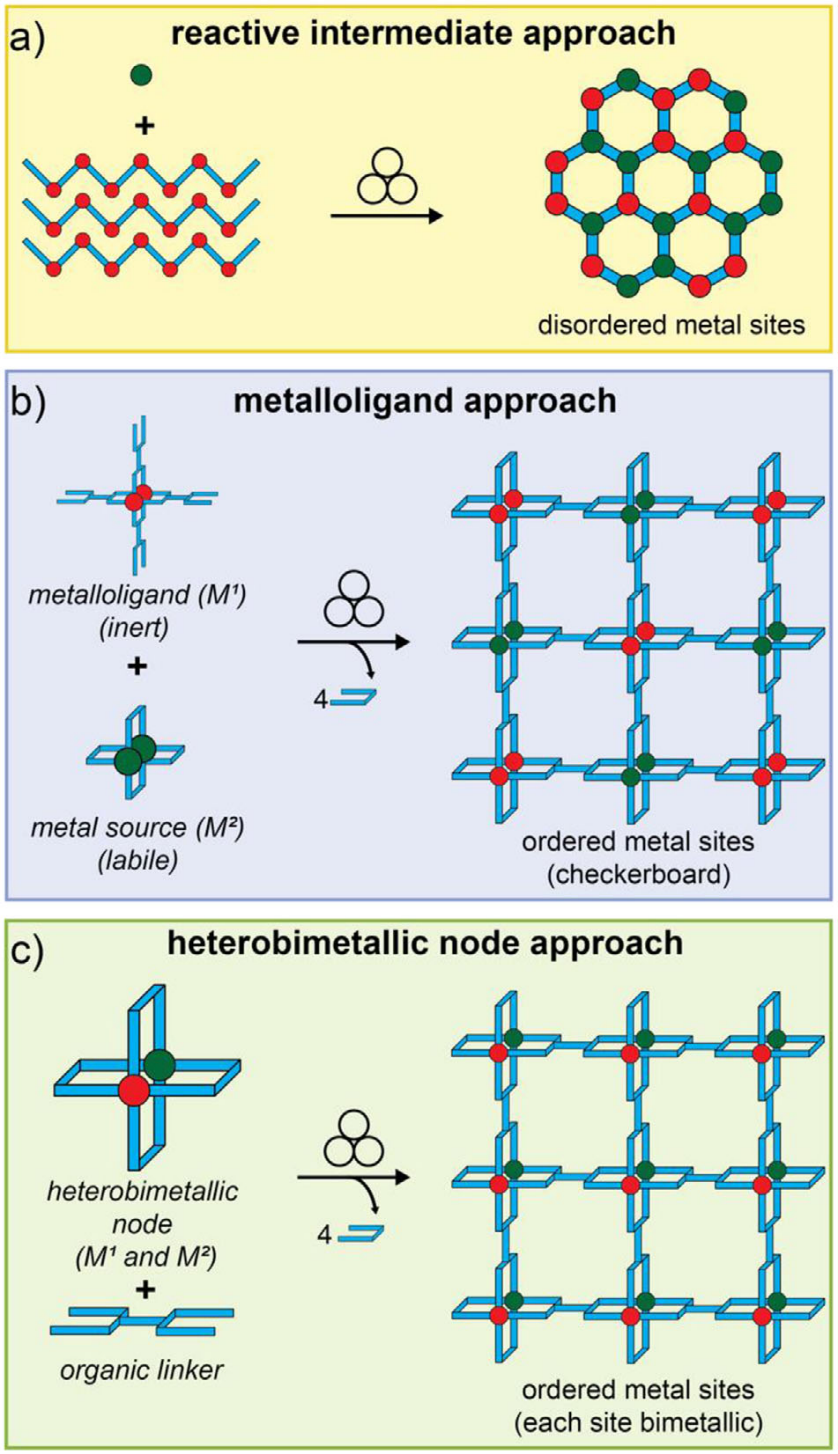
Strategies for the targeted synthesis of mixed‐metal MOFs by mechanochemistry. a) Using a pre‐assembled mono‐metallic coordination polymer as a precursor for the deliberate synthesis of a mixed‐metal MOF by milling with a secondary metal source.^[^ ^151^ ^]^ b) The metalloligand approach, where pre‐complexed assemblies of a metal node and a linker are milled with a secondary metal source to produce metallically ordered MOFs.^[^ ^152^ ^]^ c) The heterobimetallic approach which relies on using pre‐synthesized nodes containing one atom of each metal type as MOF precursors, resulting in metallically ordered MOFs.^[^ ^154^ ^]^

In addition to mechanical alloying or synthesis starting from a mixture of metal precursors, mixed‐metal MOFs can be synthesized in a targeted manner by exploiting crystalline intermediates observed *en route* to mechanochemical MOF formation (**Figure** **23**a). An example of such target‐oriented synthesis of a mixed‐metal MOF was demonstrated by Ayoub et al., who used the coordination polymer [Zn(H_2_O)_2_(H_2_ **dhta**)], observed as an intermediate during LAG mechanosynthesis of Zn‐MOF‐74 from ZnO and H_4_ **dhta**, as a precursor for the synthesis of bimetallic MOF‐74 materials.^[^ ^151^ ^]^ Notably, [Zn(H_2_O)_2_(H_2_ **dhta**)] exhibits a 1:1 metal‐to‐linker stoichiometry and can provide available metal coordination sites upon deprotonation of the H_2_ **dhta^2−^** linker, making it suitable for a reaction with one more equivalent of another metal precursor to complete the 1:2 linker‐to‐metal stoichiometry in the MOF‐74‐type framework. Thus, LAG reaction of [Zn(H_2_O)_2_(H_2_ **dhta**)] with an equimolar amount of MgO, CoCO_3,_ or Cu(OH)_2_ in the presence of MeOH as the liquid additive produced bimetallic ZnM″‐MOF‐74 materials (where M″ = Mg^II^, Co^II^, Cu^II^) in complete conversion and with high BET surface areas.^[^ ^151^ ^]^ The approach was readily expanded toward other combinations of metal nodes, by using analogous metal–organic intermediates based on Mg^II^, Ni^II^, and Co^II^, which were then milled with secondary metal sources including ZnO, MgO, CoCO_3_, or CaO. Most of the resulting bimetallic MOF materials exhibited the targeted 1:1 molar ratio of different metal sites to within 3%, a significant improvement over solution‐based methods where deviations in target compositions were reported to be in the range of 6–10%.

The mechanosyntheses of mixed‐metal MOFs described so far result in a random arrangement of metal sites in the product material. Recent efforts have shown that atomically precise (i.e., ordered) multi‐metallic MOFs can be synthesized mechanochemically if the relationship between the two different metal sites is pre‐defined by the choice of linker, node and the substitution kinetics of the metal ions used. For example,^[^ ^152^ ^]^ [Ru_2_Cu_4_(**btc**)_4_Cl] MOF was made from pre‐synthesized [Ru_2_(H_2_ **btc**)_4_Cl] paddlewheel complexes in which only one of the three carboxylic acid functionalities of the tritopic H_3_ **btc** is deprotonated. This Ru_2_‐unit is inert,^[^ ^153^ ^]^ meaning that the Ru‐**btc** bonds are very difficult to cleave. Thus, upon milling in the presence of MeOH with copper(II) acetate, a more labile^[^ ^153^ ^]^ Cu_2_‐carboxylate paddlewheel complex, the eight carboxylate units at the periphery of the Ru_2_‐based paddlewheel bind exclusively to Cu^II^ ions to assemble the desired material with a 1:2:2 stoichiometric ratio of Ru:Cu:**btc^3−^**. This mechanochemical approach, illustrated in Figure 23b (with a different stoichiometry using a ditopic linker) relies on having one metal species act as a robust metalloligand that can bind to a secondary metal ion in a predictable fashion.

Finally, an alternative approach was also proposed which relies on robust, precisely structured heterobimetallic complexes as pre‐synthesized “nodes” that are incorporated into the target framework via ligand exchange with a suitable linker (Figure 23c). Along these lines, pre‐assembled heterometallic [Pd^II^M^II^(OAc)_4_] (M^II^ = Cu^II^, Zn^II^, or Ni^II^) paddlewheel units were milled with H_3_ **btc** to form materials isostructural to HKUST‐1 but with heterobimetallic nodes.^[^ ^154^ ^]^ The materials were tested as nitrene‐transfer catalysts in allylic amination and olefin aziridination and, despite moderate to good activity of all materials, the choice of the metal M^II^ was found to have a significant effect on the relative effectiveness of the catalysts towards each reaction pathway.

Importantly, the metalloligand and heterobimetallic node strategies outlined above are effective because mechanochemical conditions avoid elevated temperatures and bulk organic solvents, which could lead to the undesired scrambling of metal sites.

## 6 Guest Encapsulation Within MOFs

Solution‐based methods for the encapsulation of target molecular species within MOFs are limited in efficiency, partially due to the competition between bulk solvent and intended guest for framework voids. Mechanochemical methods for the formation of such composite materials address this challenge by reducing, or even eliminating, the presence of solvent molecules during the guest entrapment. In that way, mechanochemical guest encapsulation can achieve higher and better controlled loading, while avoiding the often‐aggressive solvothermal conditions of traditional MOF synthesis that could lead to decomposition of sensitive guest species.

**Figure 24:**
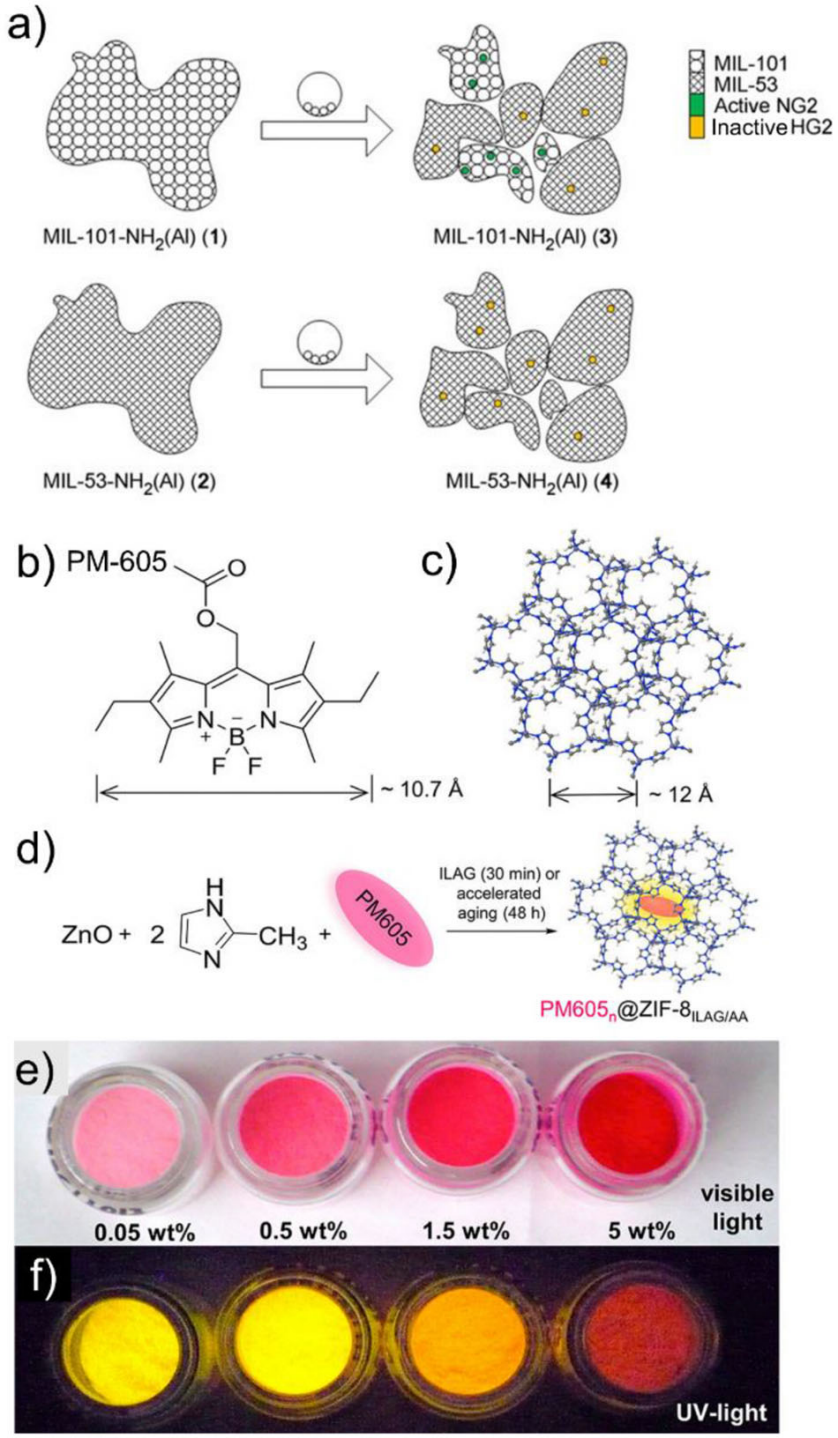
Examples of mechanochemical encapsulation of small‐molecule functional guests. a) Encapsulation of 2nd‐generation Hoveyda‐Grubbs catalyst by milling with pre‐made MIL‐type framework. b‐f) Encapsulation of luminescent BODIPY‐dye into ZIF‐8 using the ship‐around‐a‐bottle strategy: b) size match between PM‐605 dye and c) the pore size of ZIF‐8; d) scheme for the bottle‐around‐a‐ship synthesis of BODIPY@ZIF‐8 materials with resulting samples at different dye loadings under: e) visible light and f) ultraviolet irradiation. Panel a) Reproduced with permission.^[^ ^155^ ^]^ Copyright 2016, Wiley. Panels b) through f) Reproduced with permission.^[^ ^156^ ^]^ Copyright 2018, American Chemical Society.

An early example of mechanochemical encapsulation of guests within a MOF was reported by Spekreijse et al.,^[^ ^155^ ^]^ who initially attempted the solvothermal inclusion of the Hoveyda‐Grubbs 2^nd^‐generation catalyst (HG2) within the pores of MIL‐101‐NH_2_ through a “bottle‐around‐a‐ship” strategy, where the MOF would be assembled around the intended guest. The strategy was unsuccessful due to the degradation of the guest species in the hot, acidic reaction medium. In contrast, a mechanochemical synthesis based on ball‐milling pristine MIL‐101‐NH_2_ with HG2 avoided catalyst degradation and led to a high percentage of guest inclusion. PXRD analysis revealed that mechanochemical guest entrapment also involved the structural rearrangement of initial MIL‐101‐NH_2_ into MIL‐53‐NH_2_, highlighting the dynamic nature of MOF metal‐ligand bonds under mechanochemical conditions. HG2 could be incorporated within MIL‐53‐NH_2_ as the precursor MOF, but this led to a complete loss of catalytic activity. This difference was rationalized by considering that some of the original MIL‐101‐NH_2_ framework structure was retained in the former experiment, providing an immobilized but accessible catalyst species (**Figure** **24**a).^[^ ^155^ ^]^ The resulting HG2@MIL‐101‐NH_2_ MOF‐immobilized catalysts for olefin metathesis were reusable, contrasting with commercial resin‐supported catalysts whose active species are easily washed away in organic solvents.

The mechanochemical “bottle‐around‐a‐ship” guest encapsulation strategy, where a MOF is assembled in the presence of a guest and encapsulates it, was demonstrated by Glembockyte et al. with the entrapment of small luminescent organic dye molecules during MOF mechanosynthesis.^[^ ^156^ ^]^ Specifically, mechanosynthesis of ZIF‐8 by ILAG in the presence of a boron dipyrromethene (BODIPY) fluorophore PM‐605 led to the entrapment of the dye within the MOF pores as they are formed (Figure 24b–d). While crystalline BODIPY dyes are generally non‐emissive due to efficient self‐quenching, the resulting PM‐605@ZIF‐8 materials showed emission quantum yields even higher than those of PM‐605 dissolved in organic solvent (Figure 24e,f). Moreover, the encapsulated PM‐605 showed a tenfold resistance to photobleaching compared with the same dye in solution and could not be washed out of the MOF with organic solvents, presumably due to the portals lining the pores of the SOD‐topology ZIF‐8 structure being too small.^[^ ^156^ ^]^ The applicability of the “bottle‐around‐a‐ship” strategy to aging reactions was demonstrated by Brekalo et al.,^[^ ^86^ ^]^ who achieved the inclusion of a Cram's macrocycle within a novel RHO‐Zn(**Im**)_2_ material by aging ground mixtures of H**Im**, ZnO and the macrocycle as a paste with liquid DMF or DEF. Other examples of bottle‐around‐a‐ship strategy for mechanochemical synthesis of guest@MOF materials were reported for example by the Zheng group,^[^ ^157^ ^]^ who described the encapsulation of polyoxometalate guests, the Bellusci group^[^ ^158^ ^]^ who encapsulated iron oxide nanoparticles to produce magnetic MOF composites, and the Naimi‐Jamal group who encapsulated ibuprofen within copper(II)‐ and zinc‐based pillared MOFs.^[^ ^159^ ^]^

Encapsulation of buckminsterfullerene (C_60_) within the cages of ZIF‐8 also proceeded with a remarkably high degree of stoichiometric control, as reported by Martinez et al.^[^ ^160^ ^]^ Synthesis of the MOF by ILAG of ZnO, H**Meim**, and C_60_ in the presence of EtOH and NH_4_NO_3_ led to near‐complete incorporation of the fullerene into ZIF‐8 pores during milling. The effectiveness of ILAG for synthesis of C_60_@ZIF‐8 composites contrasts with solution‐based approaches which resulted in low loadings even in presence of excess of C_60_. A set of C_60_@ZIF materials with varying guest loadings could be prepared by controlling the amount of the fullerene in the reaction mixture, with a close agreement between the amount of guest added to the reaction mixture and the final amount of incorporated guest.^[^ ^160^ ^]^ Mechanochemical milling also permitted encapsulation of C_60_ within zirconium‐based multivariate MOFs.^[^ ^161^ ^]^ Other reports have shown how small molecule guests can be mechanochemically incorporated into MOFs, leading to improved proton conduction, and properties of value for applications in resonance imaging applications, catalysis, metal ion detection, and more.^[^ ^162^, ^163^, ^164^, ^165^ ^]^

An elegant way of including the API ibuprofen within the HKUST‐1 framework was demonstrated by Nawrocki and co‐workers by grinding H_3_ **btc** with a pre‐synthesized copper(II) ibuprofenato complex based on the copper(II) carboxylate paddlewheel units. Mechanical grinding led to a ligand exchange reaction to form the HKUST‐1 framework, with the released ibuprofen molecules being included as guests.^[^ ^166^ ^]^

Mechanical treatment can also serve as a homogenizing step before the MOF is assembled by other means, such as by heating. As an example, Liang et al. showed that heating of the gel produced by grinding the decamethylcucurbit[5]uril (MC5) ammonium chloride complex (MC5 ·2NH_4_Cl·4H_2_O) with H_3_ **btc** and Fe(NO_3_)_3·_9H_2_O led to the formation of monolithic MC5@Fe‐MIL‐100 composites, with MC5 trapped in the pores of the resulting MOF.^[^ ^167^ ^]^ The composite materials displayed a higher adsorption capacity toward CH_4_ than Fe‐MIL‐100 only, indicating favorable interactions between CH_4_ and the encapsulated macrocycle, and showed faster CH_4_ adsorption than the pure macrocycle, likely due to the nonporous nature of solid MC5.

**Figure 25:**
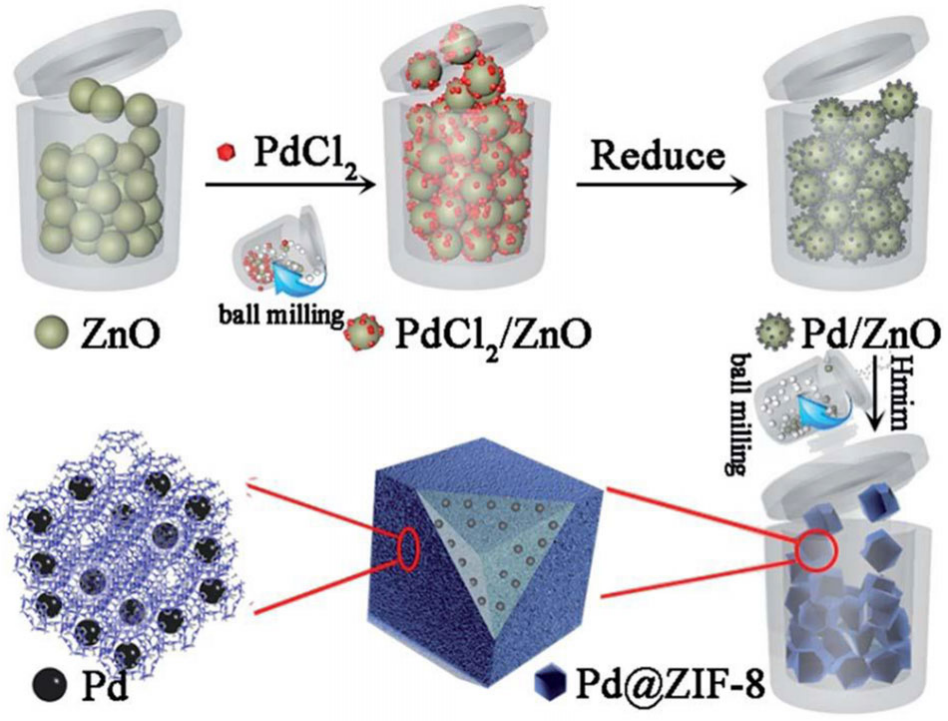
Schematic diagram of the encapsulation of palladium nanoparticles (PdNPs) into ZIF‐8 by a multi‐step ball‐milling approach where ZnO is ground with PdCl_2,_ the mixture reduced, and subsequently milled with H**Meim** to form Pd@ZIF‐8. Reproduced with permission.^[^ ^168^ ^]^ Copyright 2019, The Royal Society of Chemistry.

Metals have also been encapsulated into ZIF materials, as illustrated by the inclusion of Pd nanoparticles (PdNPs) into ZIF‐8 by ILAG synthesis from PdNPs deposited on ZnO and H**Meim** (**Figure** **25**).^[^ ^168^ ^]^ Nitrogen absorption and TEM measurements indicated that the PdNPs were not incorporated into the pores of the SOD‐topology ZIF‐8 framework but were surrounded by bulk ZIF‐8 which also served to stabilize the nanoparticles without a need for additional capping ligands. The sieving and confining effect of the MOF surrounding the catalytic nanoparticle endowed such hybrids with high hydrogenation efficiency and a preference for reaction with smaller substrates and at less‐hindered sites.

Mechanochemistry is particularly well‐suited for enzyme encapsulation into MOFs, as enzymes are more robust in a solid form than they are upon prolonged exposure to solution or solvothermal environments typical of conventional MOF synthesis. In addition, synthetic methods that use metal oxide precursors like ZnO are exceptionally mild toward enzymes because the only reaction byproduct is water. For example, Wei et al. encapsulated a range of carbohydrate‐processing and antioxidant enzymes into ZIF‐8, UiO‐66‐NH_2,_ and Zn‐MOF‐74 MOFs.^[^ ^169^ ^]^ The enzymes retained their function and showed enhanced resistance to deactivation by proteases due to the size‐filtering effect inherent to their MOF coating. The same bottle‐around‐the‐ship approach provided access to biocomposites in which a range of biological entities, including the protein bovine serum albumin (BSA) and the living bacteria *Escherichia coli* (*E. coli*), were mechanochemically encapsulated by ZIF‐90.^[^ ^170^ ^]^ Systematic studies by Vo and co‐workers on preparing composite enzyme@MOF materials using catalase (CAT), hemoglobin (HB), and lysozyme (LYZ) as substrates revealed that enzyme encapsulation can take place by gentle LAG, conducted by ball‐milling at a low frequency of 8 Hz for 10 seconds in the presence of a pH 7 buffer as the liquid additive. Notably, whereas CAT and HB were found to be readily encapsulated at a wide range of loadings, the efficiency of encapsulation was significantly smaller for LYZ. Similar outcomes were observed when encapsulation was attempted by precipitation from aqueous solution. The difference in encapsulation behavior was related to CAT and HB exhibiting lower isoelectric point (pI) values of 5.4 and 6.8, respectively, compared to the significantly higher value for LYZ being significantly higher (11.35).^[^ ^171^ ^]^ More information on mechanochemical enzyme encapsulation within MOFs, as well as within hydrogen‐bonded (HOF) and COFs can be found in a recent review by Chen and co‐workers.^[^ ^172^ ^]^

Finally, the mechanochemical inclusion of multiple guest types within MOFs offers access to composite materials with functionality arising from cooperativity between guests. For example, an efficient catalyst for the asymmetric Mannich reaction was obtained through mechanochemical encapsulation of a Cu‐ or a Ru‐based photosensitizer complex along with a wheat germ lipase within a modified UiO‐67 framework.^[^ ^173^ ^]^ In this functional material, the MOF provided a protective environment to stabilize and prevent the denaturation of the enzyme while enforcing spatial proximity between photosensitizer and enzyme to ensure efficient transport of reaction intermediates between each component.

## 7 Similarities Between Mechanochemistry of MOFs and Organic Molecular Solids

### 7.1 Reactivity Under Mechanochemical Conditions

**Figure 26:**
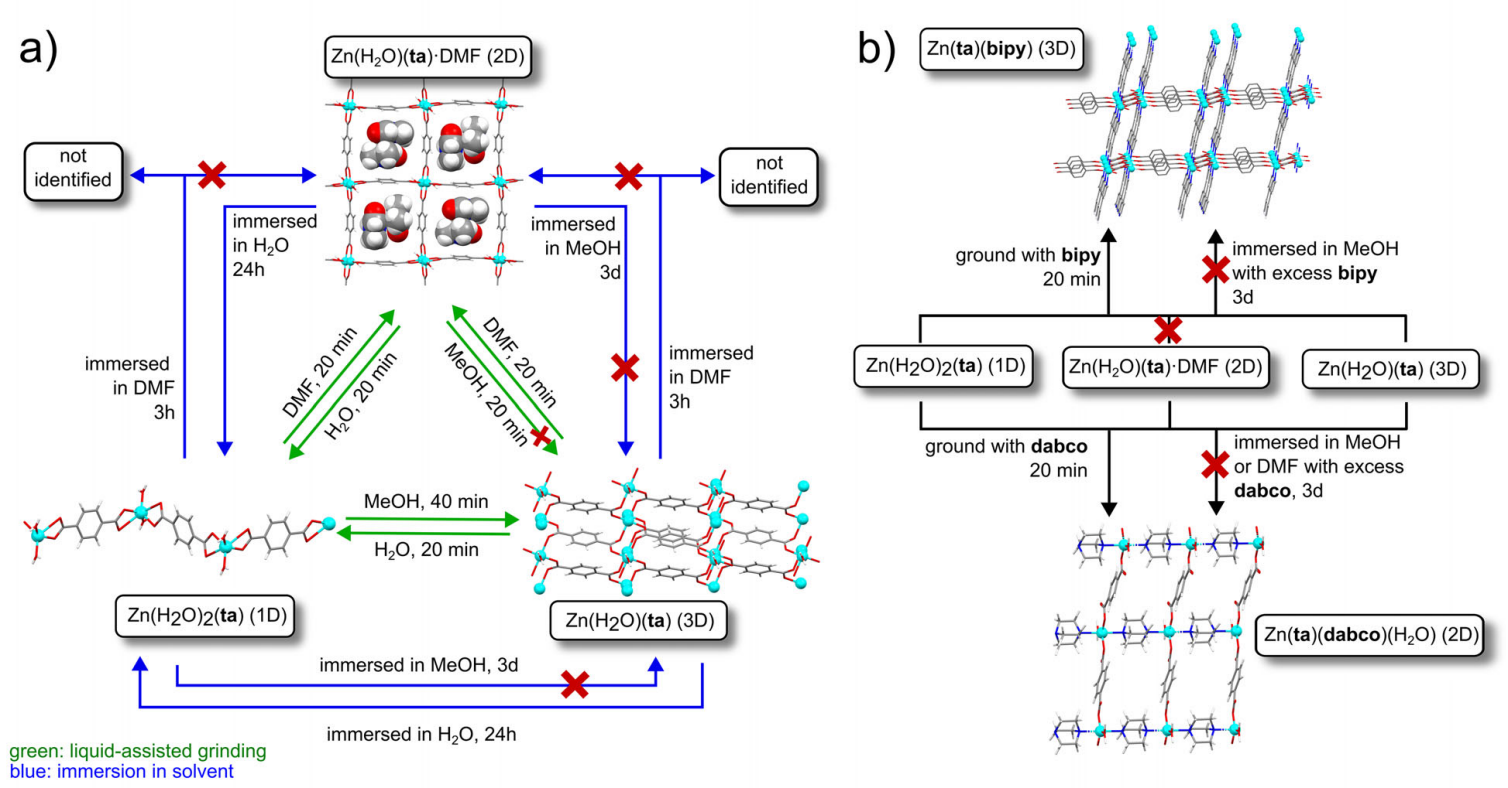
Reactivity of zinc terephthalate‐based MOFs under mechanochemical conditions, as outlined by Yuan and co‐workers.^[^ ^174^ ^]^ a) Interconversion of 1D, 2D and 3D structures of zinc terephthalate. b) Formation of mixed‐ligand MOFs based on milling of pre‐made 1D, 2D and 3D materials with additional bridging **bipy** or **dabco** ligands. Reproduced with permission.^[^ ^174^ ^]^ Copyright 2010, Wiley.

While a wide range of MOF materials can be made or post‐synthetically modified through mechanochemical techniques, the mechanical treatment itself can also introduce changes on already pre‐synthesized MOF materials. An early exploration of such behavior was reported by Yuan et al.^[^ ^174^ ^]^ (**Figure** **26**a) who reported remarkable reactivity of 1D, 2D and 3D materials based on Zn^2+^ ions and **ta^2−^** linkers. Upon grinding with DMF, the 1D coordination polymer and the 3D framework both rearranged rapidly into a 2D square‐grid layered (*sql*) topology framework which, conversely, could be converted to the 1D coordination polymer by LAG with water. The 1D polymer was readily converted to the 3D framework by LAG with MeOH, and the reverse process was readily accomplished by LAG with water. Upon introduction of additional ligands, structure reconstruction leading to more complex mixed‐ligand MOFs was also observed: milling of the 1D, 2D or 3D zinc terephthalate materials with additional **bipy** or **dabco** ligands yielded 3D Zn(**ta**)(**bipy**) or 2D Zn(**ta**)(**dabco**)(H_2_O) framework materials with new Zn─N bonds that did not exist in the precursor metal–organic materials (Figure 26b).

The observations brought to light a similitude between the mechanochemistry of MOFs or coordination polymers and that of organic solids that undergo polymorph interconversion or form higher‐order cocrystals by milling. Furthering this analogy would undoubtedly lead to improvements in the design and mechanistic understanding of mechanochemical processes in one class of materials through investigating chemically different but perhaps more readily accessible model systems. For example, real‐time monitoring of the LAG synthesis of a cocrystal of nicotinamide (**na**) and benzoic acid (H_2_ **ba**) revealed the initial formation of the polymorph Form I of the (**na**)(H_2_ **ba**) cocrystal, followed by conversion to Form II.^[^ ^175^ ^]^ Such polymorphic interconversion of a nascent cocrystal bears similarity to the stepwise formation and transformations of ZIF polymorphs observed by real‐time monitoring (Figure 11).^[^ ^59^, ^104^ ^]^ This analogy can also draw from the mechanochemistry of minerals: by milling zinc metal with sulfur and monitoring by synchrotron PXRD, the Weidenthaler group observed the formation of hexagonal ZnS (wurtzite) and its subsequent transformation into the more stable, cubic polymorph (sphalerite).^[^ ^176^ ^]^ For further discussion of similarities and differences between organic and inorganic mechanochemical reactions, we recommend a review by Boldyreva.^[^ ^177^ ^]^

**Figure 27:**
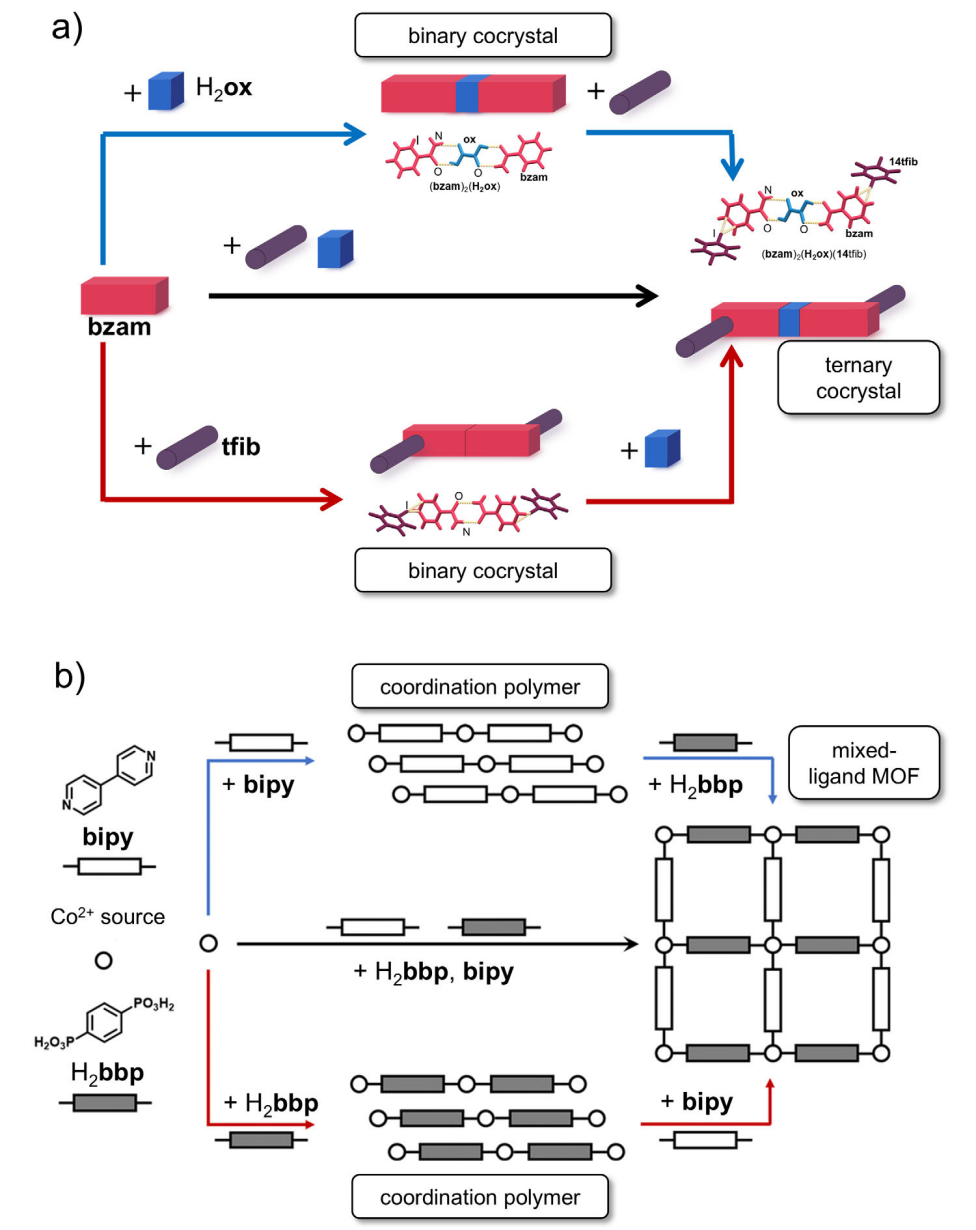
Similarity in mechanochemical synthesis of higher‐order materials by mechanochemistry. a) A ternary cocrystal is accessible by reaction of all three components, or by reaction of pre‐made, simpler binary cocrystals.^[^ ^178^ ^]^ b) A mixed‐ligand MOF is accessible through one‐pot assembly of the metal source with two distinct linkers, or by reaction of simpler coordination polymers with additional linkers.^[^ ^179^ ^]^ Panel a) adapted under the terms of the CC‐BY license.^[^ ^178^ ^]^ Panel b) adapted under the terms of the CC‐BY 3.0 license.^[^ ^179^ ^]^

The analogy further develops in the context of mechanochemical synthesis of higher‐order organic or metal–organic materials. For example, Vainauskas et al. milled a pre‐made two‐component cocrystal of benzamide (**bzam**) and oxalic acid (H_2_ **ox**), of composition (**bzam**)_2_(H_2_ **ox**), held together with O─H···O and N─H···O hydrogen bonds, with the halogen bond donor 1,4‐diiodotetrafluorobenzene (**tfib**) to form a ternary cocrystal phase of composition (**bzam**)_2_(H_2_ **ox**)(**tfib**) with additional C─I···π interactions (**Figure** **27**a).^[^ ^178^ ^]^ The ternary cocrystal was also accessible directly by one‐pot LAG of **bzam**, H_2_ **ox** and **tfib**. Such behavior parallels the behavior of zinc terephthalate MOFs outlined above^[^ ^174^ ^]^ and also resembles the reactivity observed by the Emmerling group through real‐time synchrotron PXRD monitoring of the mechanochemical assembly of a mixed‐ligand MOF composed of divalent cation nodes (Co^2+^ or Fe^2+^) crosslinked by anionic 1,4‐benzenediphosphonate (**bbp** ^2−^) and neutral **bipy** nodes (Figure 27b).^[^ ^179^ ^]^ The three‐component MOF was readily obtained by one‐pot milling, but also by step‐by‐step strategies in which the metal source was first milled with either H_2_ **bbp** or **bipy** to form an intermediate coordination polymer, followed by the addition of and milling with a second linker precursor. Interestingly, the mixed‐linker MOF was not accessible by the stepwise route starting with Fe^2+^ and H_2_ **bbp**, as the target intermediate iron‐phosphonate material did not undergo the second reaction step, and acted as a competing product.

### 7.2 Interconversion of Stoichiometry During Mechanochemical Synthesis

The previously described (Section 4.3, Figure 19) stepwise mechanism of Zn‐MOF‐74 formation^[^ ^136^, ^137^ ^]^ contrasts with the mechanisms observed for ZIF syntheses, which all involved framework polymorphs, i.e., frameworks of identical composition but different structure.^[^ ^59^, ^104^ ^]^ Specifically, in the case of Zn‐MOF‐74, the Zn(H_2_O)_4_(H_2_ **dhta**) intermediate exhibits a different metal‐to‐linker ratio than in the final MOF‐74 framework, of composition Zn_2_(**dhta**), which is rationalized by the initial activation of the most reactive acid sites of the linker. Such behavior is reminiscent of the reported stepwise mechanisms of cocrystal formation, where the stoichiometry of intermediates appears to be determined by the kinetically driven formation of the most stable supramolecular interactions or supramolecular synthon.^[^ ^180^ ^]^ An example is the cocrystallization of nicotinamide (**na**) with suberic acid (H_2_ **sub**), wherein a (**na**)(H_2_ **sub**) intermediate phase was observed *en route* to the targeted (**na**)_2_(H_2_ **sub**) cocrystal by laboratory and synchrotron PXRD, and subsequently also by terahertz Raman spectroscopy monitoring.^[^ ^181^, ^182^, ^183^ ^]^ This observation was rationalized as a kinetically‐driven formation of the product containing the strongest carboxylic acid‐amide supramolecular synthon, which subsequently transforms into the final product containing a greater number of weaker carboxylic acid‐pyridine heterosynthons.^[^ ^181^ ^]^

### 7.3 Mechanical Alloying of MOFs: Analogy to Reactions of Organic Cocrystals

**Figure 28:**
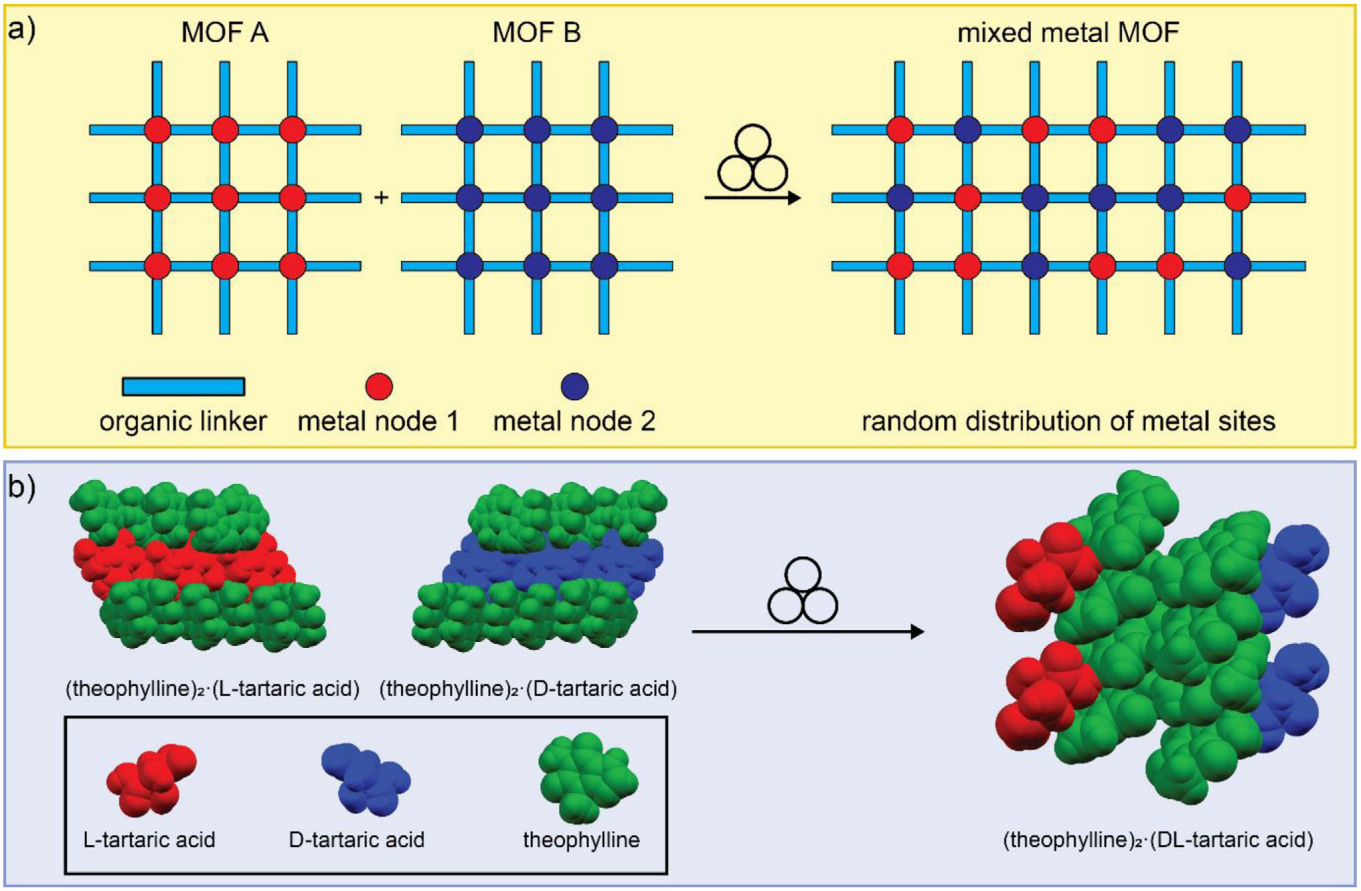
Comparison between the mechanochemical reactivity between: a) two MOFs with different metal nodes, producing a mixed‐metal MOF^[^ ^186^, ^187^ ^]^ and b) two binary cocrystals to form a ternary one, illustrated by the LAG reaction of theophylline cocrystal with D‐ and with L‐tartaric acid, resulting in a centrosymmetric cocrystal containing all three components.^[^ ^185^ ^]^

As an approach to synthesize mixed‐metal MOFs by mechanochemical reaction of pre‐made monometallic MOFs, mechanical alloying^[^ ^184^ ^]^ mirrors the reactivity of pre‐assembled crystals leading to the formation of more complex systems (**Figure** **28**a). An example of such reactivity with organic solids entails the LAG reaction of equimolar amounts of the two enantiomorphic binary cocrystals of theophylline (**tp**) with D‐ and L‐ tartaric acid (H_2_ **ta**), of composition (**tp**)_2_(D‐H_2_ **ta**) and (**tp**)_2_(L‐H_2_ **ta**), respectively, which led to the formation of a new, centrosymmetric ternary cocrystal composed of **tp**, L‐H_2_ **ta** and D‐H_2_ **ta** (Figure 28b).^[^ ^185^ ^]^ In the context of microporous MOFs, pioneering work in mechanical alloying to make new materials was presented by the Kitagawa and Horike groups,^[^ ^186^ ^]^ who synthesized mixed‐metal aluminum‐ and gallium‐based MOFs with 1,4‐naphthalenedicarboxylate (**ndc^2−^**) linkers. Specifically, ball‐milling of the Al(**ndc**)(OH) MOF with varying proportions of its corresponding gallium‐based congener Ga(**ndc**)(OH) yielded amorphous solids with a homogeneous distribution of metallic sites, which upon exposure to water vapor for three days crystallized into materials with unit cell parameters between those of Al(**ndc**)(OH) and Ga(**ndc**)(OH). The dependence of the unit cell dimensions on the Al:Ga ratio followed Vegard's law, indicating a solid solution. In contrast, amorphous solids produced by milling of Al(**ndc**)(OH) or Ga(**ndc**)(OH) with the In^3+^ analog did not crystallize upon exposure to water vapor, potentially due to a large mismatch in unit‐cell dimensions imparted by the larger In^3+^ ions. The same study also reported alloying on a set of ZIF‐8 and MOF‐74 materials, illustrating the generality of the method.^[^ ^186^ ^]^ Further evidence that MOF alloying leads to highly homogeneous solid solutions was provided with MOFs based on Cu^II^ or Fe^II^ and the 1,2,3‐triazolate (**tza^−^**) linker. Ball‐milling of the two binary Cu(**tza**)_2_ and Fe(**tza**)_2_ MOFs gave tunable crystalline bimetallic Cu_x_Fe_1‐x_(**tza**)_2_ MOFs with a highly homogeneous distribution of the two metal nodes, whereas solution synthesis led to heterogenous domains of individual Cu(**tza**)_2_ and Fe(**tza**)_2_.^[^ ^187^ ^]^ When pyrolyzed, the alloys retained bimetallic homogeneity and were highly active ORR catalysts due to the synergy between the Fe‐ and Cu‐based metal nodes.

An approach akin to mechanochemical alloying also works with layered materials. Huskić et al. explored the role of relative amounts of Al^III^ and Fe^III^ nodes (M) on the properties of [Mg(H_2_O)_6_][NaM(**ox**)_3_] materials composed of layered anionic 2D open frameworks based on the oxalate (**ox^2−^**) linker, with Mg(H_2_O)_6_^2+^ ions as counterions. This type of MOFs, first studied for their magnetic properties^[^ ^188^ ^]^ then for their high proton conductivities,^[^ ^189^ ^]^ was also identified in structures of rare Siberian minerals stepanovite (Fe^III^ nodes only, [Mg(H_2_O)_6_][NaFe(**ox**)_3_]) and zhemchuznikovite (Al^III^ and Fe^III^ nodes), which are polytypes based on ABCABC and ABAB stacking of anionic sheets, respectively.^[^ ^82^ ^]^ Whereas milling of synthetic stepanovite and its Al‐only analog [Mg(H_2_O)_6_][NaAl(**ox**)_3_] did not lead to a reaction, LAG in the presence of water yielded mixed‐metal [Mg(H_2_O)_6_][NaFe_x_Al_1‐x_(**ox**)_3_] materials in which the layering (stepanovite‐ or zhemchuzhnikovite‐type) depended upon the relative amounts of Al and Fe.

## 8 Mechanochemical Reactivity of MOFs

### 8.1 Transformation of Pre‐Synthesized MOF Materials

The practical value of rearranging structures of MOFs and coordination polymers by mechanochemical treatment was noted by Sun et al., who used ball‐milling to regenerate HKUST‐1 microporous material that had degraded upon exposure to humidity.^[^ ^190^ ^]^ Similarly, Lee et al. used LAG to restore the structures of representatives of a broader range of MOFs that have been degraded by exposure to humidity or other environmental factors.^[^ ^191^ ^]^ Restoration of degraded samples of MOF‐177, based on the Zn_4_O cluster as a node, and UiO‐67 proceeded readily through LAG protocols. A similar behavior was observed for ZIF‐65, a representative of ZIF class of materials with a Co^II^ node and 2‐nitroimidazolate (**Nim** ^−^) linkers. In contrast, the structure of MOF‐5, which hydrolytically decomposed into a mixture of hydrated zinc terephthalate polymers, could only be regenerated when milling was followed with a 2‐day immersion in DEF, in a manner reminiscent of the milling‐mediated synthesis of MOF‐5 reported by Lv et al.^[^ ^94b^ ^]^

The effect of mechanical treatment on CO_2_ sorption, comparing manual grinding using a mortar and pestle to ball‐milling, was investigated by Zelenka et al. on samples of solution‐synthesized HKUST‐1 and Gd‐based MOF‐76.^[^ ^192^ ^]^ Manual grinding of HKUST‐1, conducted occasionally every working day for 20 days on samples dispersed in MeOH, led to a significant (almost 40%) increase in CO_2_ sorption capacity of the material, which was rationalized by comminution of the particles and opening access to MOF pores. Under similar conditions, Gd‐MOF‐76 did not develop changes in its CO_2_ sorption behavior. The reproducibility of the effect of manual grinding on HKUST‐1 was confirmed by three different researchers over a period of a week, followed by PXRD and spectroscopic analysis, as well as measurement of CO_2_ sorption behavior. Ball‐milling of the MOF samples, on the other hand, consistently led to a significant reduction in the CO_2_ sorption behavior, most likely due to damage to the particle structure.^[^ ^192^ ^]^

In a mechanical post‐synthetic ligand exchange method for MOF synthesis, Jin et al. induced ligand (linker) substitution by ball milling MOFs composed of terephthalate (**ta** ^2−^) or trimesate (**btc** ^3−^) linkers with functionalized derivatives of H_2_ **ta** or H_3_ **btc**.^[^ ^193^ ^]^ This resulted in MOFs with preserved crystallinity and with significantly increased proportions of the functionalized linkers. This work represents a significant improvement on previously reported solvent‐based ligand exchange protocols from an efficiency perspective, as it circumvents long reaction times and the use of bulk solvent. This approach also produced multivariate MOFs, where ligands with different functionalities (Br‐, NO_2_‐, *etc*.) were introduced simultaneously in the framework, leading to the synthesis of mixed‐linker MOFs with a greater degree of stoichiometric control compared to solution‐based protocols. In a similar vein, Jiang and co‐workers have demonstrated ball‐milling exchange of linkers on pre‐synthesized ZIF‐8, as well as UiO‐66 materials.^[^ ^194^ ^]^ A sonication‐based approach to mechanochemically activate a copper(I)‐containing MOF suspended in bulk solvent for click‐coupling catalysis has recently been reported.^[^ ^195^ ^]^

### 8.2 Synthesis of Amorphous MOFs (aMOFs) by Milling

Non‐crystalline, amorphous MOFs, also known as MOF glasses, are expected to exhibit mechanical, electronic, optical, and other properties distinct from those of their crystalline counterparts. Early efforts toward the synthesis of such materials have focused on thermal treatment of pre‐made crystalline MOFs. For example, the Bennett group has reported that *cag*‐Zn(**Im**)_2_ (ZIF‐4) can be transformed into an amorphous material by two heating‐based approaches: 1) heating the crystalline solid to 600K to induce amorphization, followed by cooling,^[^ ^196^ ^]^ or 2) heating the crystalline solid past 863 K to produce a melt, which is then cooled to produce a melt‐quenched amorphous MOF.^[^ ^197^ ^]^ However, heating to high temperatures can result in the decomposition of MOFs, as exemplified with the decomposition of ZIF‐4 and ZIF‐62 without melting in an air atmosphere. Generally, the sensitivity of MOF components to high temperatures has been recognized as a potentially significant barrier to the broad application of such melt‐quench approaches.^[^ ^198^ ^]^

**Figure 29:**
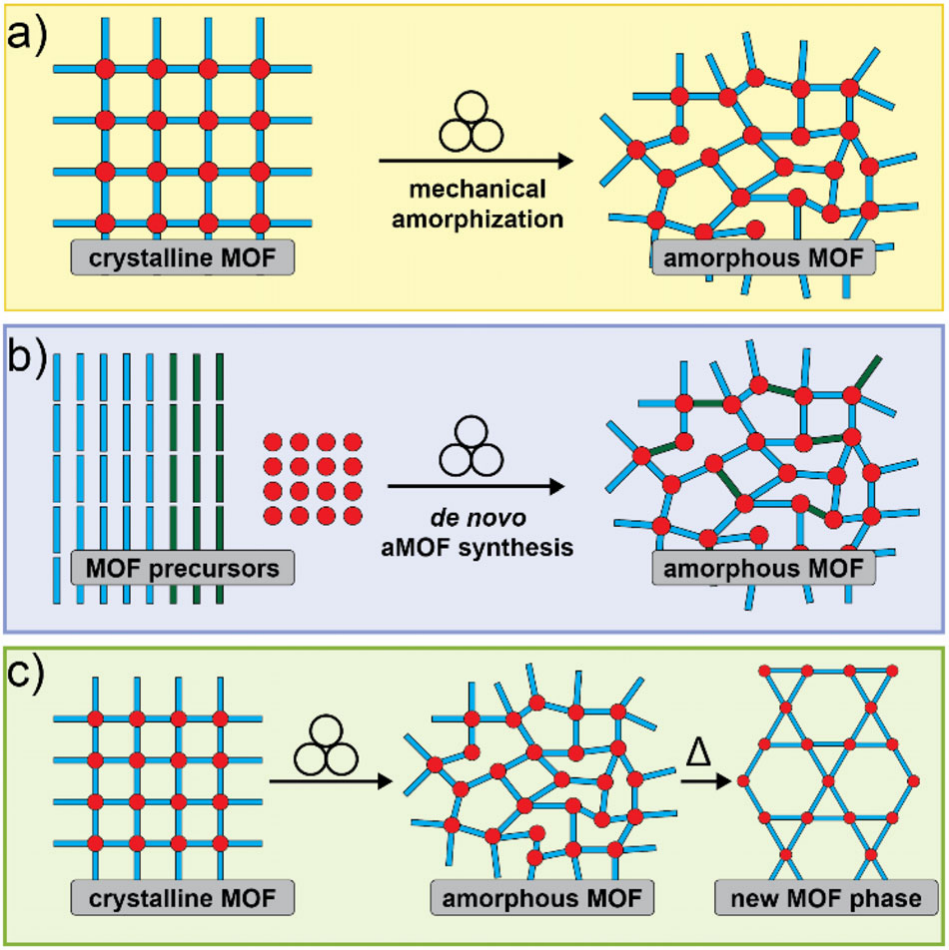
Comparison of thermal and mechanochemical transformations involving amorphous MOFs.^[^ ^199^, ^202^ ^]^ a) Mechanochemical amorphization of pre‐synthesized MOF materials. b) Synthesis of amorphous MOF materials by a mixed‐ligand strategy.^[^ ^200^ ^]^ c) Thermal treatment of mechanochemically amorphized MOFs leading to new polymorphs.^[^ ^115^ ^]^

Mechanochemistry has emerged as an alternative method for the amorphization of MOFs that is both rapid and avoids elevated temperatures. Pioneering work by Bennett et al. showed that the polymorphic Zn(**Im)_2_** frameworks ZIF‐1, ZIF‐3, and ZIF‐4, once evacuated, could be rapidly (ca. 30 min.) amorphized by ball milling (**Figure** **29**a).^[^ ^199^ ^]^ The resulting amorphous ZIFs were each nearly indistinguishable from each other by pycnometric density and X‐ray total scattering measurements, showing that the crystal structure of a parent MOF may not have a strong effect on the structure of the amorphous product it yields when exposed to grinding conditions.

A fascinating mechanochemical approach to MOF amorphization was later described by Thorne et al. who produced the mixed‐linker ZIF‐62 with the formula [Zn(**Im**)_1.70_(**Bim**)_0.30_] as a crystalline material by ball‐milling, but noticed that introducing additional benzimidazole (H**Bim**) led to an entirely amorphous material with the formula [Zn(**Im**)_1.65_(**Bim**)_0.35_] (Figure 29b).^[^ ^200^ ^]^ Mechanochemical synthesis has also been used to obtain mixed‐metal and mixed‐linker MOFs with the aim of elucidating the effect of mixed MOF compositions on melting properties.^[^ ^147^ ^]^ New crystalline materials can be discovered by using the amorphous phase as a precursor. For example, the Bennett group elucidated the structure of the previously computationally anticipated *qtz*‐Zn(**Meim**)_2_ by heating mechanochemically amorphized Zn(**Meim**)_2_ until a marked exothermic event occurred, corresponding to the crystallization of the *qtz*‐phase material whose structure was solved from PXRD data (Figure 29c).^[^ ^115^ ^]^ This result is particularly impressive since this particular phase had, to the best of our knowledge, never been reported despite many years of intense study of the Zn(**Meim**)_2_ system. Recently, a broad survey of ZIF systems revealed that ZIFs that can be melted to form amorphous phases can also be vitrified by ball‐milling, while only some of the ZIFs that do not melt can be vitrified in the same way, e.g. SOD‐Cu(**Im**)_2_.^[^ ^198^, ^201^ ^]^ Mechanochemical amorphization is also possible for other types of MOFs, for example MOF‐74 frameworks, leading to materials with modified magnetic and catalytic behavior.^[^ ^202^ ^]^ Finally, ball‐milling synthesis was reported to lead to ZIFs that melt at lower temperatures compared to solvothermally‐prepared ones, mitigating problems of decomposition in melt‐quenching synthesis of amorphous cadmium‐based ZIFs.^[^ ^28^ ^]^

### 8.3 Responses of MOF Crystals to Mechanical Force

Mechanical responses of MOFs to mechanical force and compression is an area of extensive interest and, while providing a comprehensive overview of the field would be out of the scope of this work, more information can be found in relevant literature, including reviews.^[^ ^203^ ^]^ The microscopic behavior of individual MOF crystals under the action of mechanical force was investigated by the Suslick group,^[^ ^204^ ^]^ who used TEM video recordings to observe different responses of ZIF‐8 microcrystals upon applied force, depending on whether the framework was evacuated or contained included solvent. When the material was evacuated, an individual crystal was found to undergo plastic deformation, followed by amorphization at around ca. 2000 µN. When loading was released, the resulting particle partially rebounded in size. Compression of bulk evacuated ZIF‐8 powder with a piston prompted the loss of long‐range structure (i.e., amorphization), as evidenced by PXRD data, but retention of local structure, as evidenced by X‐ray absorption spectroscopy (XAS). Overall, these observations indicated that amorphization of ZIF‐8 under mechanical treatment did not lead to defects in the immediate surrounding of metal nodes. In contrast, ZIF‐8 microcrystals that included MeOH in their pores were significantly less deformable and shattered rapidly upon application of a force as low as ca. 600 µN. Similar observations were made by other groups upon compression of ZIF‐8 crystals in a diamond anvil cell (DAC). For example, Moggach et al. found that, in the presence of MeOH, compression of a ZIF‐8 microcrystal to *ca*. 1.5 GPa led to a phase transformation that involved twisting of **Meim^−^** linkers to make the 6‐ring pores of the ZIF‐8 sodalite cages more accessible.^[^ ^205^ ^]^ The outcome is inclusion of more solvent molecules and a counter‐intuitive structure expansion upon increased pressure. In contrast, synchrotron PXRD measurements by Chapman et al. showed that treatment of ZIF‐8 crystalline powder above 0.34 GPa in a DAC in the absence of a pressure‐transmitting medium (non‐hydrostatic conditions), or in the presence of a large‐molecule fluid that cannot penetrate the framework pores (Fluorinert, hydrostatic conditions) led to irreversible amorphization.^[^ ^206^ ^]^ High‐pressure amorphization of ZIF‐8 was also found by the Huang group upon pressurization to *ca*. 39 GPa, with infrared spectroscopy indicating retention of framework connectivity.^[^ ^207^ ^]^

Overall, the observation that crystals of the same MOF can exhibit very different responses to mechanical force or applied pressure, depending on whether they contain a guest or not, is likely to be of high significance for understanding the mechanochemical behavior and reactivity of MOFs. Indeed, the outlined TEM observations on ZIF‐8 are consistent with the observation that crystalline ZIF‐8 can be synthesized by ball‐milling in the presence of liquids such as MeOH or DMF, but ball‐milling of pre‐synthesized, evacuated ZIF‐8 will lead to amorphization. It is also important to note that different MOF materials were found to show very different responses to applied force: whereas mechanical treatment of ZIF‐8 does not disrupt the local node structure, mechanical treatment of UiO‐66 led to cleavage of Zr‐linker bonds, as reported by the Suslick group using extended X‐ray absorption fine structure (EXAFS) data.^[^ ^208^ ^]^

## 9 Mechanochemistry of Covalent Organic Frameworks (COFs)

Covalent Organic Frameworks (COFs) represent an emerging class of crystalline porous organic polymers that are characterized by their permanent porosity and highly ordered structures. Since being introduced as predictable and ordered 2D^[^ ^209^ ^]^ and 3D^[^ ^210^ ^]^ porous structures, COFs have gained widespread attention across chemistry and materials science. Building on successes of mechanochemistry for MOFs, mechanochemical approaches to COFs have recently started to emerge and already demonstrated potential for synthetic simplification and accessibility in bulk scale, while avoiding prolonged solvothermal procedures and harmful solvents. Such advances include continuous production of COFs by TSE, multi‐gram scale‐up by RAM, and establishing structure‐templating effects by detection of intermediates via real‐time in situ reaction monitoring. Overall, COF mechanochemistry is off with an excellent, rapid start – just like with MOFs, the continued exploration and optimization of mechanochemical methods is expected to significantly advance COF chemistry, paving the way to new materials, new functionalities, as well as a deeper fundamental understanding and opportunities for industrial development.^[^ ^211^ ^]^

### 9.1 Imine/*β*‐ketoenamine‐Linked COFs

**Figure 30:**
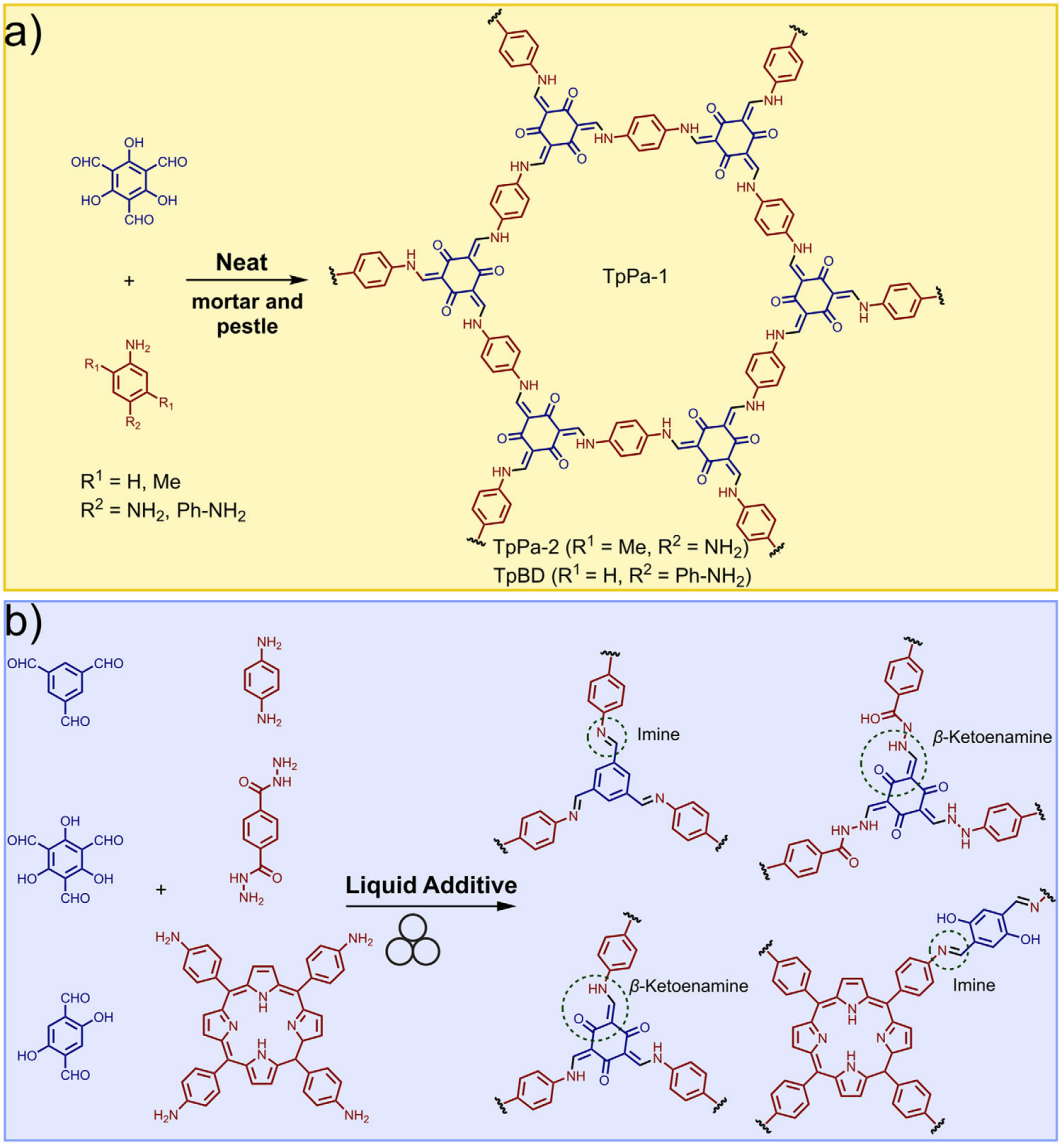
a) Mechanochemical synthesis of COFs by neat grinding^[^ ^212^ ^]^ and b) LAG mechanochemical synthesis of imine‐ and *β*‐ketoenamine‐linked COFs,^[^ ^213^ ^]^ reported by the Banerjee group.

The first mechanochemical COF synthesis was reported in 2013 by the Banerjee group who used mortar‐and‐pestle grinding to achieve room‐temperature, solventless synthesis of imine‐linked materials from amine and aldehyde components.^[^ ^212^ ^]^ Specifically, the approach generated three isoreticular COFs (**Figure** **30**a) which exhibited remarkable stability despite having a lower crystallinity than solvothermally synthesized equivalents. The COFs showed exceptional resilience to boiling water, as well as highly acidic (9 N HCl) and basic (9 N NaOH) environments. Scanning electron microscopy (SEM) and transmission electron microscopy (TEM) revealed a graphene‐like layered morphology due to the exfoliation of COF 2D layers. Since this effect was not observed for solvothermally synthesized materials, it was suggested that the mechanical forces involved during grinding were responsible for facilitating layer exfoliation.

The same group also reported the ball‐milling LAG synthesis of imine/*β*‐ketoenamine COFs, facilitated by the addition of small quantities of solvents and acetic acid, producing materials with enhanced crystallinity and porosity compared to those obtained by neat grinding (Figure 30b).^[^ ^213^ ^]^ Using acetic acid led to higher COF crystallinity, by promoting the reversibility of the imine/β‐ketoenamine linkage formation, indicating that self‐correction of the extended covalent structure is possible under mechanochemical conditions.

**Figure 31:**
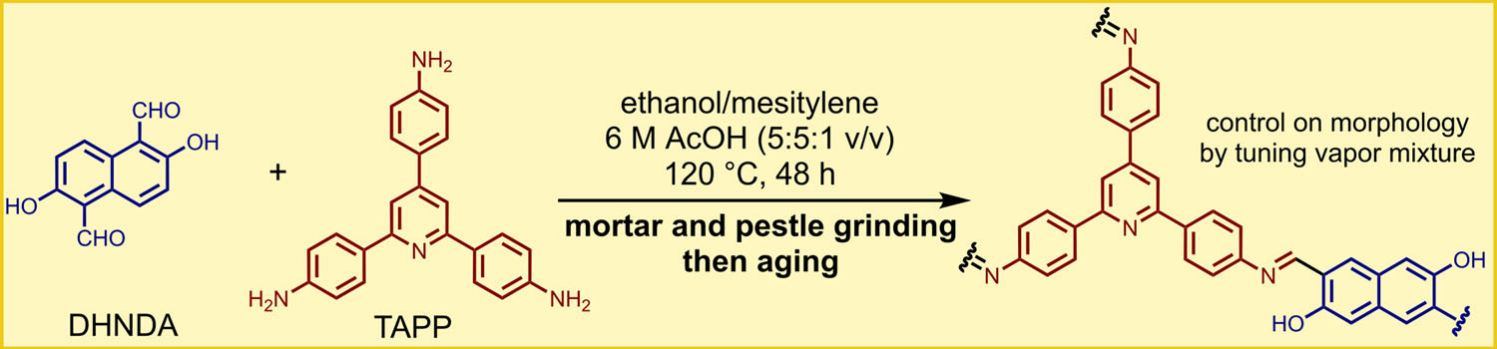
Solvent vapor‐assisted synthesis of imine‐based COF by pre‐grinding and aging in presence of ethanol/mesitylene/6M AcOH (5:5:1 v/v) at 120 °C for 48 h.^[^ ^213^ ^]^

In 2014, the Liu group reported a vapor‐assisted solid‐state approach for synthesizing COFs with controlled nanofibrous morphologies.^[^ ^214^ ^]^ While neat grinding of 2,6‐dihydroxynaphthalene‐1,5‐dicarbaldehyde (**dhnda**) with 2,4,6‐tris(4‐aminophenyl)pyridine (**tapp**) led only to the formation of oligomers, exposing the pre‐ground mixture to solvents (ethanol/mesitylene) and acetic acid vapors under autoclave conditions at 120 °C produced crystalline, porous COFs (**Figure** **31**). The study demonstrated that fine‐tuning the solvent composition could yield uniform COF structures with surface areas comparable to those of solvothermally synthesized materials. The described enhancement of the crystallinity likely stems from the error‐correction mechanism enabled by the presence of liquid additives and, especially, acetic acid facilitating the reversible imine bond formation.

The Zhao and Cheng groups explored neat grinding or kneading using a mortar‐and‐pestle to synthesize sulfonated, proton‐conductive COF materials NUS‐9 and NUS‐10.^[^ ^215^ ^]^ While the COFs after mechanosynthesis initially exhibited poor crystallinity, recrystallization in the presence of acetic acid led to highly crystalline materials. Synthesis by kneading gave COFs of high structural stability and proton conductivity, which was attributed to the successful incorporation of sulfonic acid groups.

**Figure 32:**
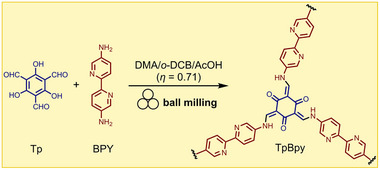
The mechanochemical LAG synthesis of the COF TpBpy for applications in PEMFCs, reported by the Banerjee group.^[^ ^216^ ^]^

Banerjee et al. reported the synthesis of a proton‐rich COF material by LAG of 1,3,5‐triformylphloroglucinol (**Tp**) and 2,2’‐bipyridine‐5,5’‐diamine (**BPY**), using mixtures of organic liquids and acetic acid as liquid additives (**Figure** **32**).^[^ ^216^ ^]^ While of lower crystallinity, the material resulting from LAG was found to outperform the solvothermally made analog as a solid‐state electrolyte in Proton Exchange Membrane Fuel Cells (PEMFCs). In contrast to the solvothermally synthesized COF, which formed a loosely packed pellet prone to breakdown during fuel‐cell operation, the mechano‐synthesized material formed a more compact, robust substrate, thus preventing the mixing of reactant gases (hydrogen and oxygen) in the fuel cell and improving the performance and the longevity of the cell. The mechanochemically synthesized COF exhibited lower crystallinity and porosity while retaining the thermal and chemical stability needed for practical applications. This was explained by the periodic channels and well‐defined pore size distribution promoting efficient proton diffusion, which is a crucial factor for their performance as solid electrolytes.

**Figure 33:**
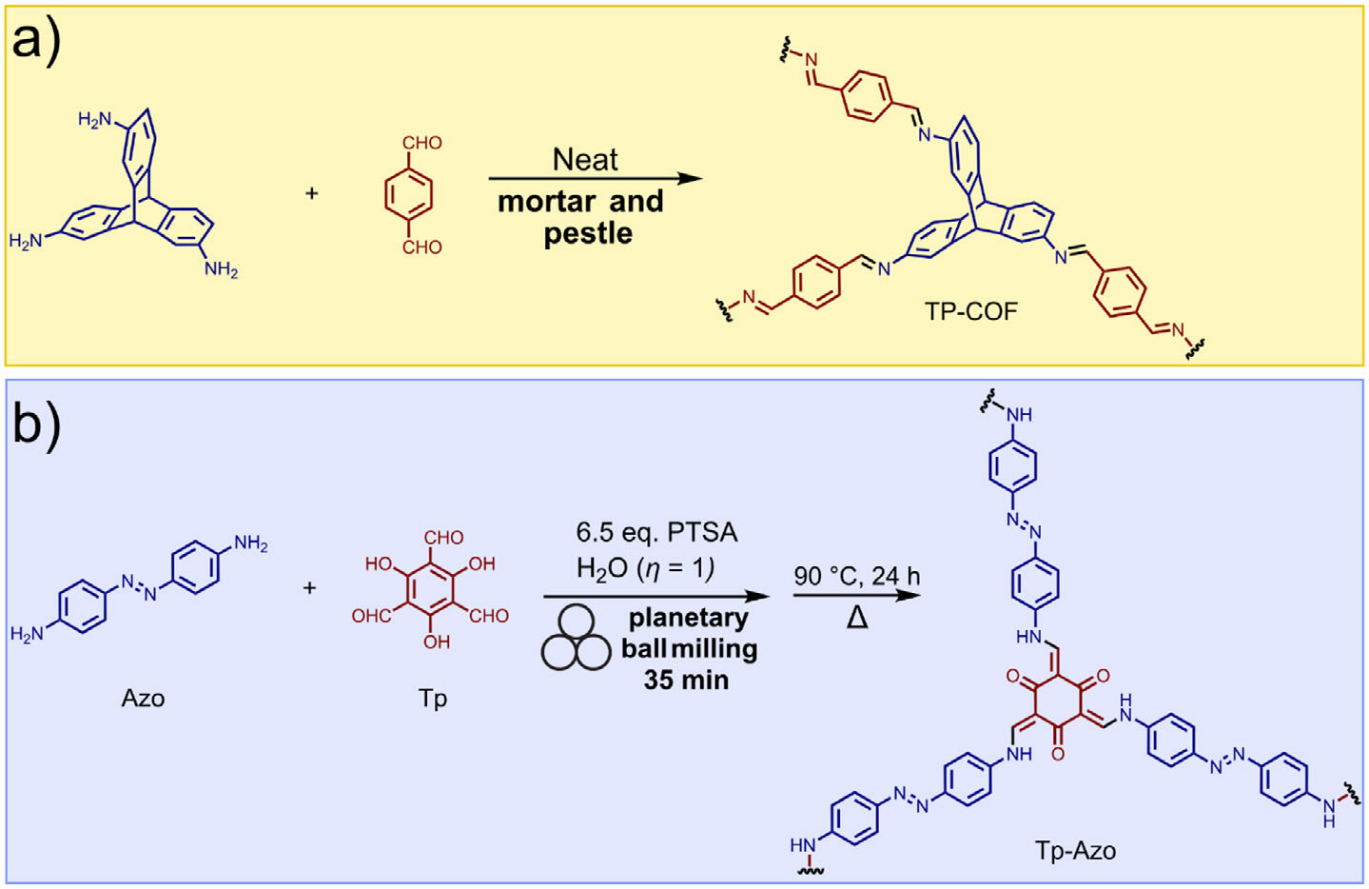
a) The mechanochemical synthesis of a triptycene‐based COF by manual grinding and^[^ ^217^ ^]^ b) the scale‐up of the mechanochemical synthesis of the Tp‐Azo COF by LAG in a planetary ball mill.^[^ ^218^ ^]^

The Roy group used manual mortar‐and‐pestle grinding to develop a mechanochemical route to a triptycene‐based imine‐linked COF (TP‐COF), aimed toward the degradation of organic dyes. The produced TP‐COF exhibited a graphene‐like layered morphology, explained by the exfoliation of the bulk COF material by mechanical shear, as well as semiconducting properties with a band gap of 2.49 eV, making it effective for photocatalysis (**Figure** **33**a).^[^ ^217^ ^]^

The scale‐up of the mechanochemical synthesis of the *β*‐ketoenamine‐linked **Tp‐Azo** COF to 0.1 kg scale was achieved by Asokan et al. through planetary ball‐milling of **Tp**, 4,4′‐azodianiline (**Azo**) and *p*‐toluenesulfonic acid (**ptsa**) (Figure 33b).^[^ ^218^ ^]^ The resulting material made by this large‐scale mechanochemical method showed considerable potential as a commercially available methane storage material.

### 9.2 Boroxine‐Linked COFs

**Figure 34:**
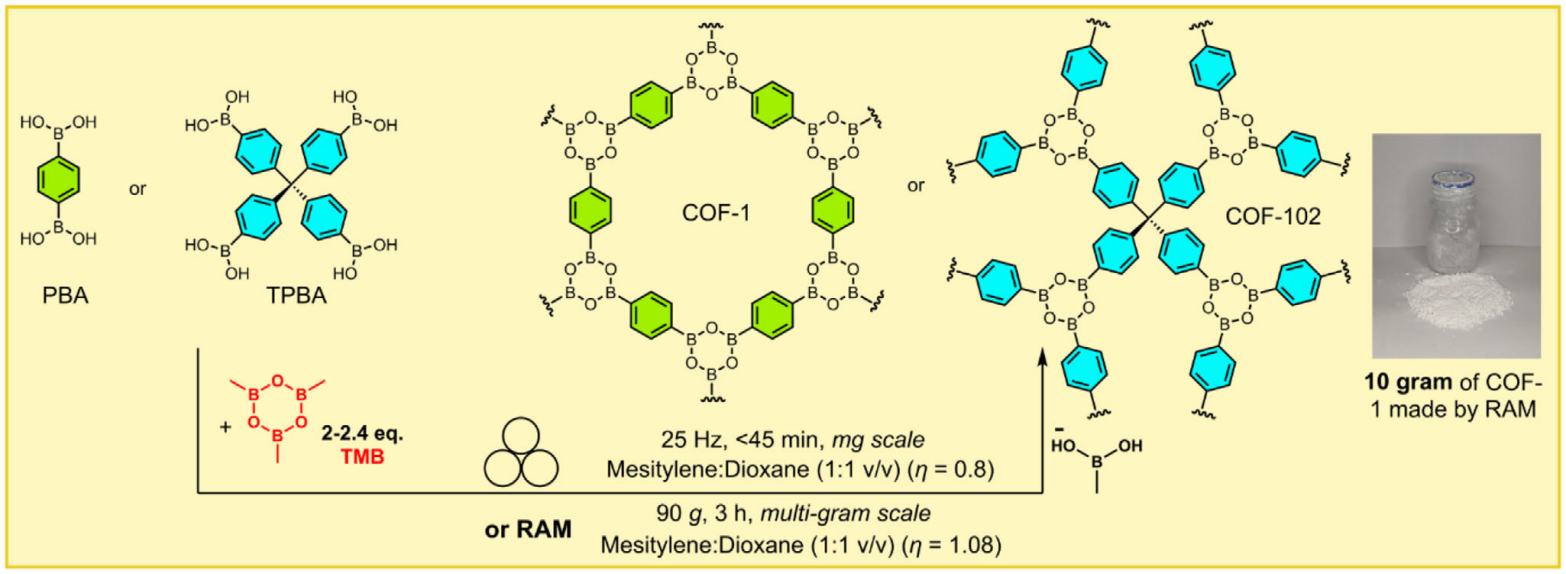
Schematic outline of the mechanochemical synthesis of the 2D (COF‐1) and the 3D (COF‐102) boroxine‐linked COFs using either a ball‐mill or a RAM instrument, in the presence of TMB as a dehydrating and modulating agent.^[^ ^224^ ^]^ Image of the COF‐1 sample courtesy of Dr F. Effaty, Institut Courtois, Université de Montréal.

Among the first and perhaps the most simple building blocks employed in the design of COFs is the boroxine ring, with boroxine‐based COFs attracting interest in the context of gas sorption,^[^ ^219^ ^]^ hydrogen isotope separation,^[^ ^220^ ^]^ photoconduction,^[^ ^221^ ^]^ and room‐temperature phosphorescent sensing.^[^ ^222^ ^]^ The majority of mechanochemical approaches to COFs have focused on materials based on imine and/or ketoenamine condensations because the hydrolytic stability of imine bonds is sufficiently high to allow framework assembly in the presence of the reaction byproduct, water. In contrast, the low hydrolytic stability of boroxine links and a near‐unity equilibrium constant^[^ ^223^ ^]^ make the mechanosynthesis of boroxine COFs challenging. Hamzehpoor et al. have shown, however, that using trimethylboroxine (**TMB**) as a modulating dehydrating reagent can enable the formation of highly crystalline porous 2D and 3D boroxine‐linked COFs via either ball‐milling or RAM (**Figure** **34**).^[^ ^224^ ^]^ These mechanochemical syntheses of boroxine COFs were readily scalable, with application of RAM readily producing the COFs in quantities ranging from hundreds of milligrams to ten grams without the need for re‐optimization. In that sense, the RAM synthesis of COFs illustrates the highly promising potential of RAM for simple synthetic scaling‐up, already noted in small‐molecule organic synthesis,^[^ ^69^ ^]^ cocrystal formation^[^ ^225^ ^]^ and MOF synthesis.^[^ ^12^ ^]^ The synthesized COFs exhibited high surface areas and crystallinity, comparable to those obtained through traditional solvothermal methods, with COF‐102 exhibiting a surface area greater than 2 600 m^2^/g – among the highest reported for mechanochemically made‐framework materials.^[^ ^224^ ^]^

### 9.3 Triazine‐linked COFs

In a recent report, the Borchardt group developed a mechanochemical approach to cyclotrimerization of nitriles and obtained triazine‐linked COFs by ball milling.^[^ ^226^ ^]^ Specifically, mechanochemical grinding of 1,4‐dicyanobenzene in presence of four equivalents of trifluoromethanesulfonic acid (TfOH) in a perfluoroalkoxy alkane (PFA) plastic jar equipped with a zirconia ball enabled a fast, efficient, and solvent‐free strategy for creating triazine‐containing materials. In this synthesis, the mechanical energy input had an influence on the outcome of the COF structure, with higher milling frequencies and longer reaction times favoring a material based on eclipsed (AA) stacking of COF sheets, while lower milling frequencies or shorter reaction times led to lower porosity frameworks with staggered (AB) stacking of 2D open COF sheets. The resulting COFs exhibited good crystallinity and porosity, with specific surface areas up to 650 m^2^/g. Notably, the COFs were also accessible by RAM but did not exhibit a high surface area. Together with the synthesis of boroxine COFs, this report illustrates the potential of both ball‐milling and RAM for COF synthesis beyond imine condensation reactions.

### 9.4 Mechanochemical Exfoliation of COFs

Exfoliation is the process of breaking down bulk materials into thinner layers, especially nanosheets or 2D structures. In the context of COFs, it involves transforming bulk COFs, which are typically conglomerated layered structures, into nanosheets to facilitate processing and integration of the material into membranes. Exfoliation of COFs can be performed mechanochemically. For example, by extending the mortar‐and‐pestle grinding time for the synthesis of *β*‐ketoenamine‐linked **TpPa‐1** COF reported by the Banerjee group (Figure 30a),^[^ ^212^ ^]^ the Jin group observed a transformation from amorphous COF particles to honeycomb‐like COF nanosheets by SEM and TEM analysis.^[^ ^227^ ^]^ The exfoliation process was reported to improve the COF nanosheets surface area. In another report, Wang et al. used ball‐milling to disperse the *β*‐ketoenamine‐linked COF synthesized by Schiff‐base reaction of 1,3,5‐tris(4‐formylphenyl)benzene and 2,6‐diaminoanthraquinone from bulk material into redox‐active nanosheets of several layers thickness.^[^ ^228^ ^]^ The exfoliated nanosheets were then used as cathode materials in lithium ion batteries with significantly improved performance compared to pristine COF materials.

### 9.5 Mechanochemical Encapsulation in COFs

As a class of porous materials, COFs have been investigated as matrices for encapsulating active components like catalysts or enzymes, with the typical aim to protect the active substance or enhance its performance in catalysis or other applications. Gao et al. established a LAG protocol in a planetary ball mill to synthesize enzyme@*β*‐ketoenamine‐linked COF materials by one‐pot encapsulation of enzymes simultaneously with the mechanochemical formation of the framework material.^[^ ^229^ ^]^ The protocol was exemplified with three different COF matrices assembled from **Tp** and either *p*‐phenylenediamine, 2,5‐dimethyl‐*p*‐phenylenediamine or benzidine, with enzyme components being cytochrome C (**Cyt C**), horseradish peroxidase (**HRP**) (both heme enzymes), or lipase PS. Encapsulation enhanced the stability and lifespan of the enzymes, making the resulting enzyme@COF materials potentially suitable for biocatalytic applications. The method demonstrates significant recyclability, with enzymes retaining up to 80% of their activity after multiple reuse cycles.

Brown et al. investigated vibratory ball milling for the rapid, in situ encapsulation of palladium (Pd) within COFs, so that the palladium is uniformly dispersed and securely integrated within the porous framework.^[^ ^230^ ^]^ The use of palladium acetate, Pd(OAc)_2_, improved catalyst stability, efficiency, and reusability for chemical reactions such as the Suzuki‐Miyaura coupling. Three imine‐linked Pd@COFs were investigated in this study, synthesized using specific aldehydes, 2,5‐dimethoxyterephthalaldehyde (**dmtp**) or 4,4′,4″‐(1,3,5‐triazine‐2,4,6‐triyl)tribenzaldehyde (**ttb**), and anilines, 1,3,5‐tris(4‐aminophenyl)benzene (**tpb**) or 4,4′,4′′‐(1,3,5‐triazine‐2,4,6‐triyl)trianiline (**tta**). The **dmtp‐tbp**, **dmtp‐tta** and **ttb‐tta** COFs demonstrated the excellent adaptability of imine‐linked frameworks for encapsulating catalytically active Pd centers.

### 9.6 Mechanisms and Intermediates in the Mechanochemical Formation of COFs

The application of tandem methods for real‐time in situ synchrotron X‐ray powder diffraction and Raman spectroscopy monitoring of COF materials, reported by the Lotsch group,^[^ ^231^ ^]^ revealed the formation of small‐molecule intermediates during the LAG synthesis of imine‐based material LZU‐1 formed by condensation of 1,3,5‐triformylbenzene (**tb**) and *p*‐phenylenediamine. The real‐time measurements highlighted a discrete Schiff‐base compound arising from condensation of **tb** and the diamine in a 1:3 stoichiometric ratio, with included molecules of the liquid additive 1,4‐dioxane engaged in hydrogen‐bonding interactions. Not only do these observations provide a mechanistic snapshot of synthetic intermediates, but the structural templating between the liquid additive and reaction intermediates is an exciting advance toward understanding structure formation and reaction pathways under LAG conditions.

**Figure 35:**
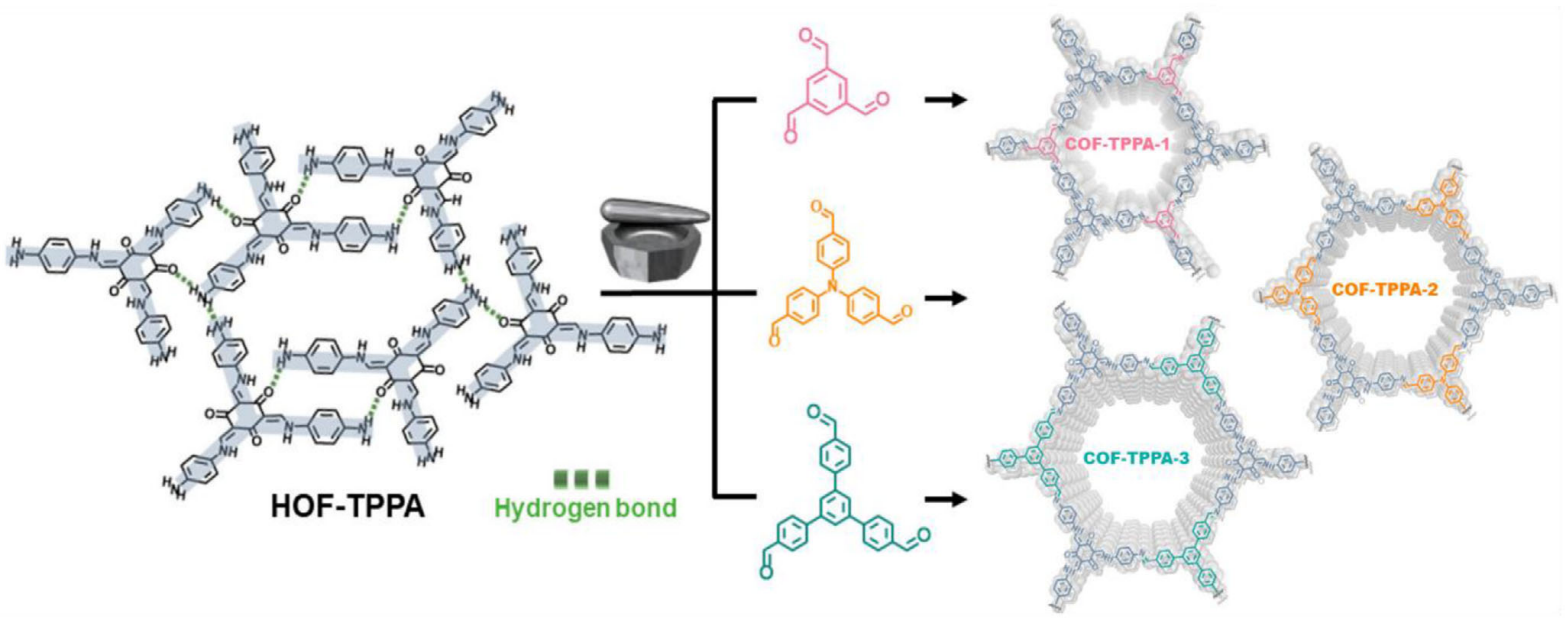
Illustration of the mechanochemical synthesis of open COF materials by imine condensation reaction of diverse aldehydes with a pre‐organized imine‐substituted hydrogen‐bonded framework material, reported by the Gu group. Reproduced with permission.^[^ ^232^ ^]^ Copyright 2023, American Chemical Society.

Besides templating, supramolecular interactions are also key to establishing order at a large scale. The Gu group described the transformation of a preorganized hydrogen‐bonded organic framework (HOF) material, HOF‐**TPPA**, into three different COFs (**Figure** **35**).^[^ ^232^ ^]^ The **TTPA** molecule resulted from the condensation of 2,4,6‐triformylphloroglucinol (**tp**) and a tenfold excess of *p*‐phenylenediamine (**pa**), and displayed a network of hydrogen bonds in the solid state to form the material HOF‐**TPPA**. Kneading the crystalline HOF‐**TPPA** with three different aldehydes in a mortar and pestle, using *n*‐butanol or *o*‐dichlorobenzene liquid additives and tris(pentafluorophenyl)borane as a Lewis acid catalyst, led to three isotopological COFs: COF‐TPPA‐1, COF‐TPPA‐2 and COF‐TPPA‐3, respectively. The transformations were confirmed by time‐dependent PXRD and FT‐IR studies and rationalized by the similarity between HOF‐**TPPA** and its isotopological COF counterparts.^[^ ^232^ ^]^ In contrast, using amorphous **TPPA** led to an amorphous material, indicating that preorganization of supramolecular interactions, such as hydrogen bonding, is essential to the formation of COFs with isotopological structures and high crystallinity.

**Figure 36:**
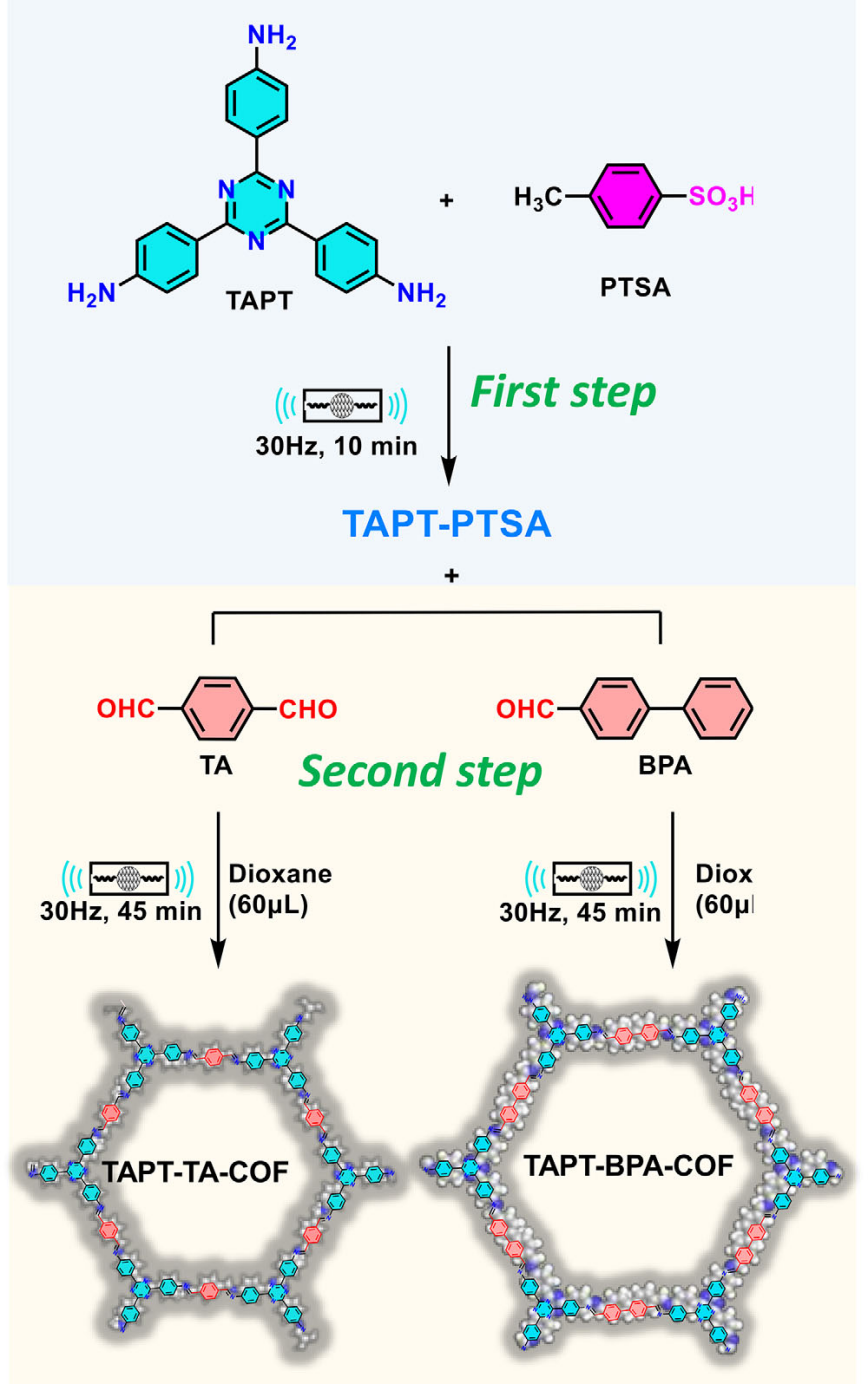
Illustration of the stepwise mechanochemical synthesis of a COFs, involving a PTSA‐based intermediate, described by Chen et al. Reproduced with permission.^[^ ^233^ ^]^ Copyright 2024, Wiley.

Recently, Chen et al. described a stepwise strategy for synthesizing imine‐linked COFs using a hydrogen‐bond‐regulated mechanochemical approach (**Figure** **36**).^[^ ^233^ ^]^ Ball‐milling of 4,4’,4’’‐(1,3,5‐triazine‐2,4,6‐triyl)trianiline (**tapt**) and **ptsa** led to a precursor material containing protonated **tapt**, with a layered structure exhibiting a network of hydrogen‐bonding and interlayer π–π interactions. Further one‐pot reaction with dialdehydes led to the formation of highly crystalline and porous COF materials. Mechanistic investigations revealed that hydrogen bonding within the solid precursor modulated the nucleophilic behavior of **tapt** amine functionalities, promoting sequential and orderly condensation reactions, while preventing the formation of amorphous polymers. This study highlights cocrystallization, or even salt formation, as a means to regulate the stacking and condensation processes relevant for COF assembly.

The kinetic profile of the mechanochemical formation of boroxine‐based COF‐1 reported by Hamzehpoor et al. (Figure 34) was investigated spectroscopically for both ball‐milling and RAM reaction designs.^[^ ^224^ ^]^ Stepwise, ex situ monitoring of the LAG ball‐milling process by Fourier‐transform infrared (FT‐IR) spectroscopy indicated an initial reaction rate of 6.7 × 10^−3^ s^−1^, but the reaction progress could not be fitted to a simple kinetic model. The reaction under RAM conditions was monitored in situ and in real time by Raman spectroscopy, revealing slower reaction kinetics (initial rate of 3.1 × 10^−4^ s^−1^), with COF formation and reactant consumption both following an apparent first‐order kinetic law.^[^ ^224^ ^]^ To the best of our knowledge, this study represents the first comparison of reaction kinetics under ball‐milling and RAM conditions.

### 9.7 Continuous Synthesis of COFs by Twin‐Screw Extrusion (TSE)

**Figure 37:**
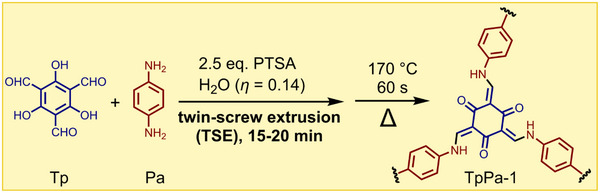
Large‐scale synthesis of the *β*‐ketoenamine‐linked COF TpPa‐1 by TSE.^[^ ^234^ ^]^

The possibility to achieve continuous mechanochemical synthesis of COFs was demonstrated by the Banerjee group with the TSE‐based synthesis of the *β*‐ketoenamine‐linked COF **TpPa‐1** (**Figure** **37**).^[^ ^234^ ^]^ This exciting methodology was based on extruding a mixture of the **Tp** aldehyde and *p*‐phenylenediamine (**Pa**) in the presence of water as a liquid additive and **ptsa** as both an acid catalyst for the Schiff‐base condensation reaction and a structure modulator. The material produced by extrusion was subsequently heated at 170 °C for 60 s, ultimately yielding the crystalline and porous target COF.

## 10 Conclusion

In summary, we have provided an overview of mechanochemistry in the context of synthesis, reactivity, and functionalization of MOF and COF materials. The combination of mechanical activation with a non‐conventional, completely or nearly solventless environment provides opportunities not only for greener syntheses, but also for materials discovery and for understanding and exploration of complex types of reactivity such as alloying of MOFs. The simplicity of mechanochemical approaches to MOF synthesis is sometimes surprising, with many of the popular archetypal classes of MOF materials being amenable to direct and rapid (minutes or hours) synthesis from the simplest and often most economically and environmentally acceptable feedstocks. This is illustrated by making ZIFs and BIFs from metal oxides or carbonates, by direct synthesis of UiO‐ and NU‐type zirconium MOFs directly from simple chloride‐based precursors or easily pre‐assembled acetate clusters, as well as the synthesis of MIL‐type materials directly from hydrated aluminum sulfate. The simple accessibility of MOFs with diverse structures through mechanochemical techniques translates into potential for manufacturing, for example through continuous twin‐screw extrusion, and into opportunities to generate materials suitable for fundamental studies of stability and structure‐property relationships. By being able to readily access homologous MOFs based on, for example, zinc, cadmium or mercury, mechanochemistry under identical conditions provides a chance to systematically investigate the behavior of different metal nodes and discover new structures and structure‐controlling effects.

In almost 20 years since early literature reviews touched upon the potential future role of mechanochemistry in the synthesis of materials such as MOFs,^[^ ^235^ ^]^ the field has grown immensely, with mechanochemistry of MOFs now being a mature, established area, and mechanosynthesis perceived as a rapid, readily accessible, and competitive route to such materials. The benefits of mechanochemistry in the MOF area are now starting to spread in the context of COFs, including advancing the understanding of structure templating and framework formation mechanisms, enabling faster, simpler synthesis without using bulk solvents or harsh solvothermal conditions, as well as the creation of new materials and scale‐up strategies. Although often overlooked, the herein noted analogies between the mechanochemistry of MOFs and organic solids, such as cocrystals and polymorphs, and the emergent similarities between MOF and COF mechanosyntheses are important in several ways. On one hand, they open the door to recognizing the generalities of mechanochemistry for materials synthesis. On the other, they can provide a source of inspiration to innovate in each one of the three areas through cross‐pollination of ideas. Finally, the lessons already learned in the context of manufacturing of organic solids, such as amorphization or the need for control of polymorphism, should provide excellent guidelines for the design of methodologies to manufacture MOFs and COFs in a more effective, low‐risk manner.

We expect that the potential of mechanochemistry to generate innovation in the MOF and COF fields will be augmented by the emergence of new, advanced techniques to predict the structures and properties of organic solids, MOFs, and potentially also COFs, as well as by the creation of increasingly sophisticated methodologies for real‐time studies and control of mechanochemical reactivity. In that context, we see potential in the recently reported methods that can probe mechanochemical reactions in more than one way, for example through acoustic, spectroscopic, or thermal measurements^[^ ^236^, ^237^ ^]^ that can couple process monitoring with chemical kinetics monitoring. Similarly, the combined use of fingerprint‐ and terahertz‐region (i.e., low‐frequency) Raman spectroscopy^[^ ^183^ ^]^ can enable simultaneous monitoring of the changes to covalent and supramolecular structure in a laboratory setting, enabling detailed studies of mechanochemical reactivity without the need for a synchrotron source. Looking into the future, such mechanistic investigations will be essential to rationalize mechanochemical reactions, predict product outcomes, and design increasingly advanced materials, after which the easy scale‐up provided by many mechanochemistry techniques will undoubtedly open paths for commercial applications.

## Conflict of Interest

The authors declare no conflict of interest.
